# Identification of 13 Guanidinobenzoyl- or Aminidinobenzoyl-Containing Drugs to Potentially Inhibit TMPRSS2 for COVID-19 Treatment

**DOI:** 10.3390/ijms22137060

**Published:** 2021-06-30

**Authors:** Xiaoqiang Huang, Robin Pearce, Gilbert S. Omenn, Yang Zhang

**Affiliations:** 1 Department of Computational Medicine and Bioinformatics, University of Michigan, 100 Washtenaw Avenue, Ann Arbor, MI 48109, USA; xiaoqiah@umich.edu (X.H.); robpearc@umich.edu (R.P.); gomenn@med.umich.edu (G.S.O.); 2 Departments of Internal Medicine and Human Genetics and School of Public Health, University of Michigan, 100 Washtenaw Avenue, Ann Arbor, MI 48109, USA; 3 Department of Biological Chemistry, University of Michigan, 100 Washtenaw Avenue, Ann Arbor, MI 48109, USA

**Keywords:** SARS-CoV-2, COVID-19, TMPRSS2, drug, docking, molecular dynamics

## Abstract

Positively charged groups that mimic arginine or lysine in a natural substrate of trypsin are necessary for drugs to inhibit the trypsin-like serine protease TMPRSS2 that is involved in the viral entry and spread of coronaviruses, including SARS-CoV-2. Based on this assumption, we identified a set of 13 approved or clinically investigational drugs with positively charged guanidinobenzoyl and/or aminidinobenzoyl groups, including the experimentally verified TMPRSS2 inhibitors Camostat and Nafamostat. Molecular docking using the C-I-TASSER-predicted TMPRSS2 catalytic domain model suggested that the guanidinobenzoyl or aminidinobenzoyl group in all the drugs could form putative salt bridge interactions with the side-chain carboxyl group of Asp435 located in the S1 pocket of TMPRSS2. Molecular dynamics simulations further revealed the high stability of the putative salt bridge interactions over long-time (100 ns) simulations. The molecular mechanics/generalized Born surface area-binding free energy assessment and per-residue energy decomposition analysis also supported the strong binding interactions between TMPRSS2 and the proposed drugs. These results suggest that the proposed compounds, in addition to Camostat and Nafamostat, could be effective TMPRSS2 inhibitors for COVID-19 treatment by occupying the S1 pocket with the hallmark positively charged groups.

## 1. Introduction

Although several mRNA or protein-based coronavirus disease 2019 (COVID-19) vaccines (Pfizer-BioNTech, Moderna, Johnson & Johnson/Janssen, AstraZeneca, Chinese and Russian COVID-19 vaccines, etc.) have been authorized for emergency use in the United States (https://www.fda.gov/emergency-preparedness-and-response/coronavirus-disease-2019-covid-19/covid-19-vaccines (accessed on 28 March 2021)) or introduced elsewhere, the development of anti-COVID-19 drugs is still of high necessity for COVID-19 disease treatment. Developing and bringing to market a brand new drug for treating a specific disease usually takes more than 10 years. Given that there are many approved or clinically investigational drugs, efforts to repurpose available drugs to target critical proteins involved in the SARS-CoV-2/host interaction pathway are desirable [1,2].

The pathogen that causes COVID-19, severe acute respiratory syndrome coronavirus 2 (SARS-CoV-2), invades hosts by hijacking the angiotensin-converting enzyme 2 (ACE2) using its spike protein, followed by employing a host enzyme, transmembrane protease serine 2 (TMPRSS2), to prime the spike at the S2′ cleavage site to expose its hydrophobic fusion peptide for fusing with the membranes of host cells [3,4,5]. Normally, it would be ideal to develop drugs to target the SARS-CoV-2 proteins (e.g., the spike protein) rather than the host proteins to reduce the side effects. However, SARS-CoV-2 is an RNA virus whose genome is prone to mutation [6]; the alteration of some amino acids on a target viral protein may make a drug ineffective. Thus, a desirable alternative would be anti-SARS-CoV-2 drugs that target key host proteins.

TMPRSS2 is a viable anti-SARS-CoV-2 host protein target for the following four reasons. First, it is not mutation-prone. Second, TMPRSS2 is used by other coronaviruses (e.g., SARS-CoV and MERS-CoV) and by influenza A viruses for the activation of surface glycoproteins; therefore, a specific TMPRSS2 inhibitor may treat a whole class of diseases caused by different pathogens [6], including SARS-CoV-2 variants, during this pandemic and in coming years. Third, TMPRSS2 does not appear to play an essential role in any organ, as other proteases may provide a degree of redundancy; thus, TMPRSS2 inhibition may have few on-target side effects. In TMPRSS2-knockout mice, TMPRSS2 appeared dispensable for normal development, growth, and organ function [7]. Fourth, since TMPRSS2 is a member of the serine protease family for which many inhibitors are available [6], finding a suitable drug to target it should be feasible.

There have been multiple studies aimed at repurposing and screening available drugs to target TMPRSS2 [8,9,10,11,12,13,14,15,16,17]. Soon after the outbreak of COVID-19, Hoffmann et al. demonstrated that SARS-CoV-2′s dependence on TMPRSS2 for cell entry can be blocked by a clinically proven protease inhibitor, Camostat [3]. A metabolite of Camostat, 4-(4-guanidinobenzoyloxy)phenylacetic acid (GBPA, known as FOY-251) also inhibited TMPRSS2 but with reduced efficiency compared to Camostat [8]. Later, numerous research groups proved that Nafamostat has about 10-fold greater potency than Camostat for preventing SARS-CoV-2 infection through in vitro and in vivo studies [9,11,12,18]. Meanwhile, Shrimp et al. suggested that Gabexate was a potential TMPRSS2 inhibitor with an IC_50_ of about 130 nM [11], but other studies reported that Gabexate was not able to effectively inhibit viral infections even at a high concentration of 10 µM [9,12]. Some studies identified the United States Food and Drug Administration (FDA)-approved bromhexine as an inhibitor of TMPRSS2 at a concentration of 750 nM [19], while other in vitro studies reported that bromhexine could not inhibit TMPRSS2 at all [11,20]. Hempel et al. carried out a systematic analysis to compare Nafamostat, Camostat, and GBPA to determine how these compounds could effectively inhibit TMPRSS2 [18]. Their computational studies suggested that the three compounds contain the positively charged guanidinobenzoyl and/or aminidinobenzoyl moiety, which can form stable salt bridge interactions with the negatively charged aspartic acid Asp435 in the S1 pocket of TMPRSS2, occupying the binding site and leading to the inhibition.

**Figure 1:**
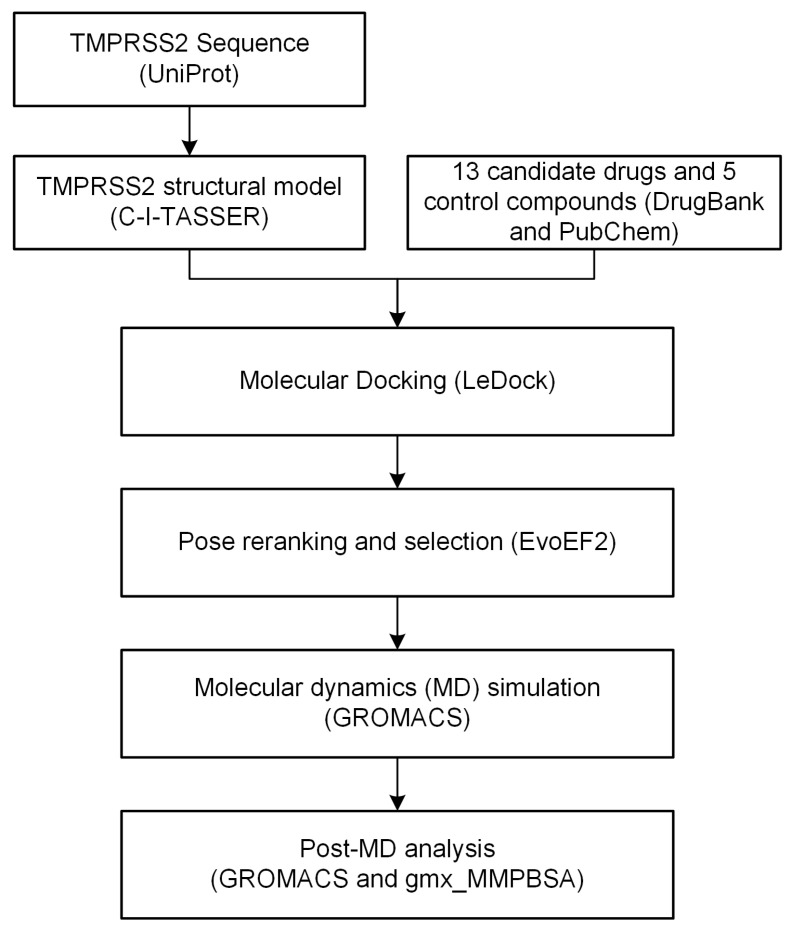
The workflow carried out in this study. The database, software, and/or web server used in each step are shown in parentheses.

Inspired by these studies, we hypothesized that some other guanidinobenzoyl- or aminidinobenzoyl-containing drugs may act as TMPRSS2 inhibitors. We identified from DrugBank [21] a narrowed list of 13 compounds (three FDA-approved drugs and 10 investigational drugs) that contain guanidinobenzoyl or aminidinobenzoyl groups (Figure S1). We computationally evaluated their potency for inhibiting TMPRSS2 through molecular docking, molecular dynamics (MD) simulation, and post-MD analysis, such as molecular mechanics/generalized Born surface area (MM/GBSA) [22]-binding free energy calculations. Figure 1 outlines the workflow carried out in this study. Consistent with previous experiments showing that Camostat and Nafamostat are strong inhibitors of TMPRSS2, our computational data revealed that Camostat and Nafamostat can form stable binding interactions with TMPRSS2. Besides Camostat and Nafamostat, our computational findings suggest that the other 11 drugs may also function as potential TMPRSS2 inhibitors, as their guanidinobenzoyl or aminidinobenzoyl groups formed very stable salt bridges with Asp435 in the S1 pocket, occupying TMPRSS2′s active site and preventing the spike from binding to TMPRSS2. This short list of promising drugs may be of great interest to biochemists and pharmacologists for further experimental tests.

## 2. Results

### 2.1. TMPRSS2 Sequence and Structural Model

**Figure 2:**
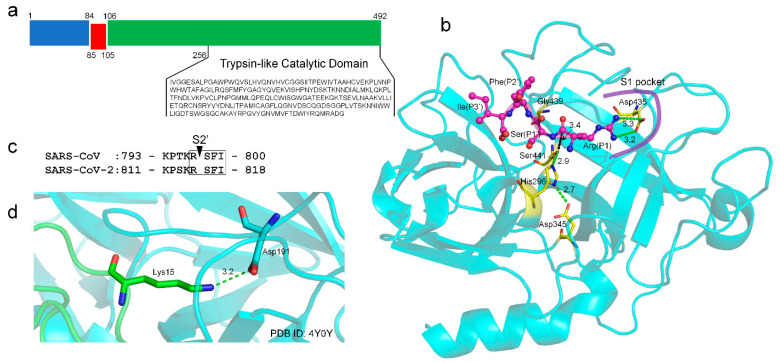
Sequence, topology, structural model, and function of TMPRSS2. (**a**) Sequence and domain topology of TMPRSS2 (amino acids 1-84: cytoplasmic domain, 85-105: transmembrane domain, 106-492: extracellular domain, and 256-492: catalytic domain). (**b**) C-I-TASSER model of the TMPRSS2 catalytic domain (shown in cyan cartoon). The conserved, catalytic triad (Ser441, His296, and Asp345); oxyanion holes (mainchain amide groups of Ser441 and Gly439); and the conserved aspartic acid (Asp435) in the S1 pocket are shown in yellow sticks. A tetrapeptide (RSFI, shown in a magenta ball-and-stick model) extracted from the SARS-CoV-2 S2′ cleavage site is docked into the binding site using HPEPDOCK. Hydrogen bonds and salt bridge interactions are illustrated in dashed green lines. The distance between the atom Oγ of Ser441 and the carbonyl C atom of P1 arginine is illustrated in dashed black lines. The distances are shown around the lines (unit: Å). (**c**) The S2′ cleavage sites of SARS-CoV and SARS-CoV-2. (**d**) An example of the trypsin inhibitor interaction in the S1 pocket.

TMPRSS2 (UniProt ID: O15393) has two isoforms produced by alternative splicing. Isoform 1 (O15393-1) has been chosen as the canonical sequence and was used in this study. The full-length TMPRSS2 isoform 1 is composed of 492 amino acids, containing two topological domains (amino acids 1-84, cytoplasmic domain and 106-492, extracellular domain), one transmembrane domain (amino acids 85-105), and one trypsin-like catalytic domain (amino acids 256-492) (Figure 2a). Isoform 2 does not have a catalytic domain and was excluded from the analysis.

There is no experimental structure available for TMPRSS2 and its domains. We used a deep-learning contact-guided protein structure assembly approach, C-I-TASSER [23], to model the structure of the catalytic domain of TMPRSS2 (Figure 2b). The model had a C-score [24] of 0.45, which corresponded to the estimated TM-score [25] of 0.89. Here, the C-score was a confidence score for estimating the global quality of predicted models by C-I-TASSER; based on large-scale benchmark tests, C-I-TASSER models with a C-score > −2.5 correspond to a correct fold with a TM-score > 0.5. The model also had high local structure quality, with a MolProbity [26] score of 0.91 (Figure S2), which ranked at the 100th percentile. This puts the structure models amongst the best structures of a comparable solution by comparison with a representative set of experimental structures collected from the Protein Data Bank (PDB) [27]. Our model exhibited a very high structural similarity (e.g., TM-score > 0.95) to the reported models [28,29] generated by homology modeling approaches. Compared with the homology models built on a single template, our C-I-TASSER model was constructed by considering the consensus of multiple templates (PDB IDs: 7meq, 3w94, 4dgj, and 6eso) and, thus, avoided the modeling bias toward a single experimental structure.

A typical feature of trypsin or a trypsin-like protease is the deeply buried negatively charged aspartic acid in the S1 pocket, which specifically recognizes the positively charged arginine or lysine at the P1 site of a protein substrate. In TMPRSS2, such an aspartic acid residue is Asp435 (Figure 2b); there is no other aspartic acid residue in the S1 pocket. Besides, the catalytic elements of TMPRSS2 include a well-established catalytic triad (Ser441–His296–Asp345) indicated by the hydrogen-bonding network and two oxyanion holes (i.e., the main-chain amide groups of Ser441 and Gly439) (Figure 2b). The ideal configuration of these catalytic elements also suggested a good quality of the TMPRSS2 model. TMPRSS2 can prime the spike proteins at the S2′ cleavage site for SARS-CoV and SARS-CoV-2 (Figure 2c). We used a protein–peptide docking tool HPEPDOCK [30] to predict the binding mode of the P1-P1′-P2′-P3′ tetrapeptide (RSFI, Figure 2c) extracted from SARS-CoV/SARS-CoV-2 bound to the binding pocket of TMPRSS2. In the top one pose, the P1 arginine was predicted to form bidentate salt bridge interactions with Asp435, while the Oγ atom of the catalytic Ser441 is 3.4 Å to the carbonyl atom of the P1 arginine (Figure 2b) within the van der Waals contact distance (3.5 Å) that is critical for the subsequent bond-breaking catalysis. The predicted binding interactions mimic those made between trypsin and its natural substrate, in which the lysine side-chain amino group interacts with the conserved aspartic acid in the S1 pocket [31] (Figure 2d).

### 2.2. Guanidinobenzoyl- or Aminidinobenzoyl-Containing Drugs

**Figure 3:**
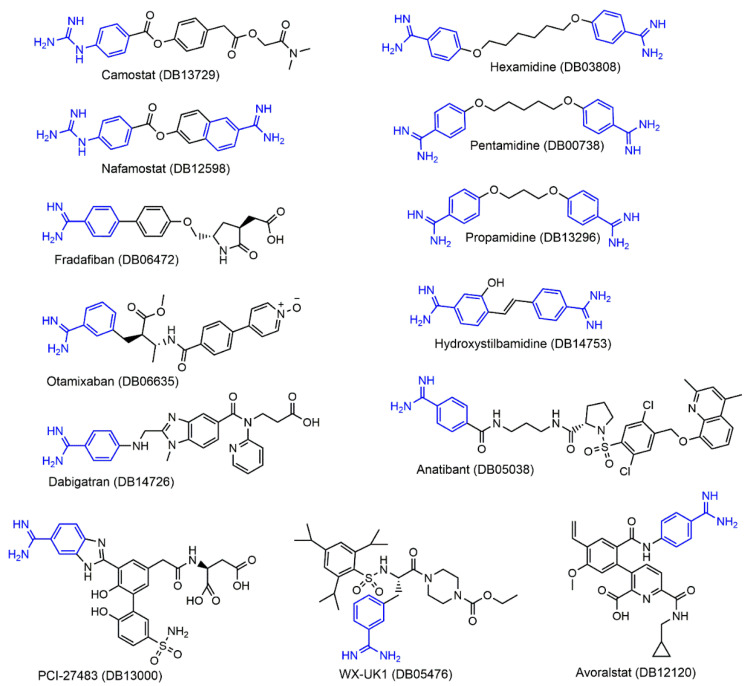
Molecular formulas of the 13 drugs investigated in this work. The guanidinobenzoyl and aminidinobenzoyl groups are highlighted in blue.

Previous studies revealed that a positively charged group that mimics arginine or lysine in a natural substrate of trypsin was important for a drug acting as an inhibitor to the trypsin-like TMPRSS2 [18,28,32]. Specifically, the guanidinobenzoyl and/or aminidinobenzoyl group in Camostat or Nafamostat could form stable binding interactions with the conserved Asp435 in the S1 pocket in TMPRSS2 and lead to inhibition. Compared with Camostat and Nafamostat, Gabexate, which contains an arginine-like side-chain, showed only a weak inhibitory potency [9,11]. Considering that a drug’s rigidity is crucial for high-affinity binding due to its low conformational entropy effect [33,34,35], we preferentially considered drugs with guanidinobenzoyl or aminidinobenzoyl rather than an arginine- or lysine-like side-chain group; the former two groups have far fewer degrees of freedom and are, hence, more rigid. We searched DrugBank for FDA-approved or investigational drugs that contain guanidinobenzoyl or guanidinobenzoyl and obtained a small library of 13 drugs (Figure 3). The drug name, DrugBank ID, regulatory status, and primary indication of these drugs are listed in Table 1.

Among the 13 drugs, Camostat is a serine protease inhibitor approved in Japan for the treatment of chronic pancreatitis and postoperative reflux esophagitis. Nafamostat is a synthetic serine protease inhibitor approved as an anticoagulant therapy for patients undergoing continuous renal replacement therapy due to acute kidney injury and used for the treatment of acute pancreatitis in Japan. Camostat and Nafamostat were demonstrated to be effective TMPRSS2 inhibitors [8,9,12] and are in clinical trials for COVID-19 treatment (ClinicalTrials.gov (accessed on 28 March 2021) Identifier: NCT04321096 for Camostat and NCT04352400 for Nafamostat). It was speculated that Camostat and Nafamostat are covalent TMPRSS2 inhibitors, because their ester bonds can be cleaved by serine proteases [18,32,36]; this speculation was supported by their low nanomolar-level inhibitory behaviors [11,18,28,32]. In contrast, as shown in Figure 3, the other drugs do not contain a cleavable ester bond adjacent to the guanidinobenzoyl or aminidinobenzoyl group and may only function as a noncovalent TMPRSS2 inhibitor. The three FDA-approved drugs Pentamidine, Hexamidine, and Hydroxystilbamidine are primarily used for the treatment of pneumocystis pneumonia, acanthamoebiasis, and nonprogressive blastomycosis, respectively. Except for Camostat and Nafamostat, none of the other drugs have been clinically investigated for COVID-19 treatment.

### 2.3. Molecular Docking Suggests Salt Bridge Interactions between Guanidinobenzoyl or Aminidinobenzoyl and Asp435

Each of the 13 drugs was docked into the putative binding pocket of TMPRSS2 using LeDock [37], as a previous comprehensive evaluation of ten docking programs on a diverse set of protein–ligand complexes suggested that LeDock had the best sampling power [38], which is important for predicting the correct binding mode. The docked poses were clustered with an RMSD cutoff of 2 Å, and a maximum of 20 cluster center poses were saved for analysis. Different numbers of clustered poses were generated for distinct TMPRSS2 drug systems (Table 2 and Table S1).

The binding energy for all the poses was rescored using our physics-based energy function EvoEF2 [39], because the default LeDock score function may tolerate severe inter-molecular steric clashes in a few top-ranked poses, which achieved high EvoEF2-binding scores (Table S1). For instance, for the drug Anatibant, the first and 10th poses had LeDock-binding scores of −8.60 and −7.16 kcal/mol, respectively; however, they were scored at −10.34 and −34.21 EEU (EvoEF2 energy units) and reranked as the 17th and first best poses by the EvoEF2 score. Consistent with the experimental data that Nafamostat acts as a stronger TMPRSS2 inhibitor than Camostat [9,11,12,18], we found that the top poses of Nafamostat obtained more favorable (i.e., lower/more negative) binding scores than those of Camostat by both LeDock and EvoEF2 (Table 2 and Table S1). The LeDock scores for both drugs were not much different: −8.02 kcal/mol for Camostat and −8.61 kcal/mol for Nafamostat. However, their EvoEF2 scores were much-better distinguished: −27.02 EEU for Camostat and −34.70 EEU for Nafamostat. Compared with Nafamostat, some other drugs, such as Pentamidine, Hexamidine, WX-UK1, Otamixaban, Avoralstat, PCI-27483, and Dabigatran, had even more favorable best LeDock or EvoEF2-binding scores (Table 2 and Table S1), indicating that they may also be potent inhibitors of TMPRSS2.

**Figure 4:**
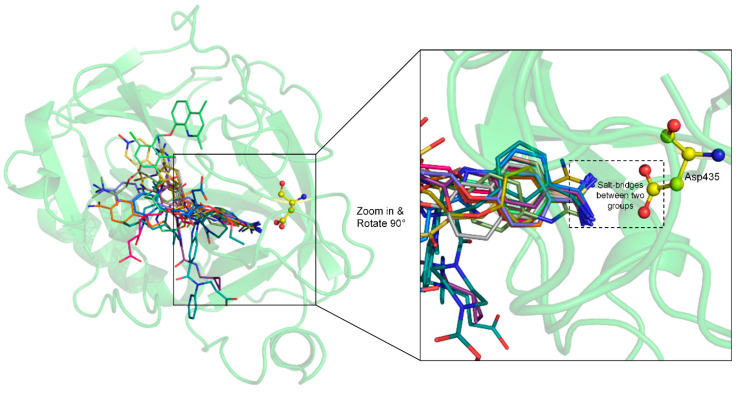
Superposition and comparison of the ligand poses with the lowest EvoEF2 scores for all 13 drugs. TMPRSS2 is shown in the green cartoon model, with residue Asp435 depicted in the yellow ball-and-stick model. The zoom-in inset shows that guanidinobenzoyl or aminidinobenzoyl can form salt bridge interactions with the Asp435 carboxyl group (shown in the dashed box).

The molecular docking results indicated that all the drugs had at least one pose that could form salt bridge interactions with the negatively charged Asp435 (Table S1). Note that Anatibant had only one pose (i.e., pose 10) involved in the putative salt bridge interactions, which was reranked as the best pose by EvoEF2, with the lowest energy score (Table S1). The top one pose with the lowest EvoEF2 score is shown in Figure 4 for each of the drugs. All the drugs are well-docked into the binding pocket of TMPRSS2, with their guanidinobenzoyl or aminidinobenzoyl groups aligning in the S1 pocket and forming salt bridge interactions with the side-chain carboxyl group of Asp435 (see also Table S1), supporting favorable binding scores.

**Figure 5:**
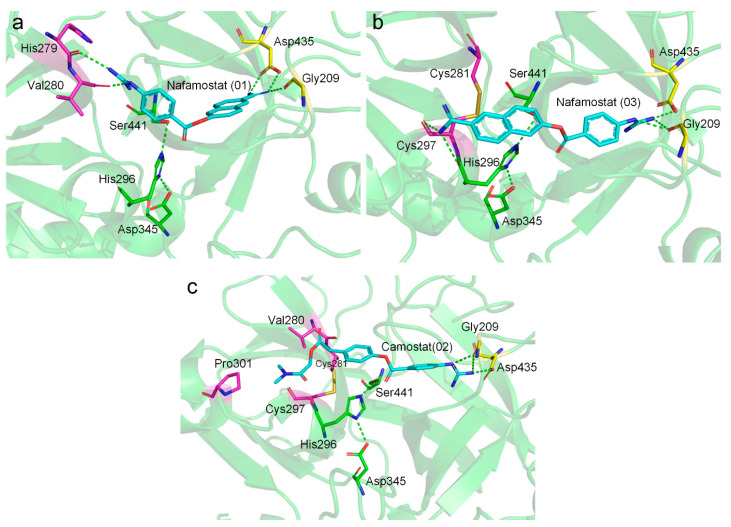
Putative binding modes of Nafamostat (**a**,**b**) and Camostat (**c**). Ligands, catalytic triad residues, binding residues in the S1 pocket, and other important binding residues outside of the S1 pocket are shown in cyan, green, yellow, and magenta sticks, respectively. Salt bridges and hydrogen bonds are shown in green dashed lines. Poses 1 and 3 of Nafamostat represent the “forward” and “reverse” binding modes, respectively.

Among these putative TMPRSS2 inhibitors, the binding modes of Nafamostat and Camostat have been extensively studied. We found that Nafamostat could form salt bridge interactions with Asp435 with either its aminidinobenzoyl or guanidinobenzoyl group (Figure 5a,b), which was consistent with the “reverse” and “forward” binding modes of Nafamostat described by Hempel et al. [18]. In both cases, the other positively charged group could form hydrogen-bonding interactions with the main-chain carbonyl groups (Figure 5a,b). The binding mode of Camostat was similar to the “forward” mode of Nafamostat; the noncharged terminal of Camostat was aligned into a hydrophobic pocket enveloped by Val280, Cys297, and Pro301 (Figure 5c). This binding pattern was similar to those reported in previous studies [18,32].

A previous study reported a few weak TMPRSS2 inhibitors without a guanidinobenzoyl or aminidinobenzoyl group, including Bromohexine (PubChem CID2442), 0591-5329 (CID765269), 4401-0077 (CID2882138), 4554-5138 (CID5395514), and 8008-1235 (CID693919), with an IC_50_ of 0.75, 0.93, 2.68, 1.37, and 2.64 µM, respectively, for inhibiting TMPRSS2 [19]. Therefore, these compounds could be used as control molecules to examine the 13 target drugs. The chemical formulas of these inhibitors are shown in Figure S3. These molecules were also docked to the TMPRSS2 model using LeDock following the same procedure. As shown in Table S1, the best docked poses of these control molecules in general had much higher LeDock and EvoEF2 scores than the 13 proposed drugs.

### 2.4. MD Simulations Reveal High Stability of the Putative Salt Bridge Interactions

Docking revealed potentially strong binding between TMPRSS2 and the investigated drugs, but docking cannot tell to what extent the binding interactions will be stable for a duration of time. Besides, since the ligands were forcefully docked into the binding pocket, a pose may adopt a highly constrained conformation that may not be stable in reality.

To overcome the limitations of molecular docking, we carried out MD simulations to examine the binding stability between TMPRSS2 and the drugs. Before MD, the top ten poses (if they existed) ranked by EvoEF2 for each drug were parameterized using the ACPYPE [40] program with the AM1-BCC [41,42] charge model. Note that pose 4 for drug WX-UK1 and poses 6, 7, 10, 13, and 14 for drug PCI-27483 failed to be parameterized because of severe intramolecular steric clashes. The number of poses that can be successfully applied to MD for the 13 drugs were 10 (DB00738), 10 (DB03808), 10 (DB05038), 9 (DB05476), 8 (DB06472), 10 (DB06635), 7 (DB12120), 6 (DB12598), 5 (DB13000), 10 (DB13296), 10 (DB13729), 10 (DB14726), and 4 (DB14753), respectively (Tables S2 and S3 and Figures S4–S7).

TMPRSS2 in complex with each suitable drug pose was subjected to a long-time (100 ns) MD simulation using GROMACS v2020.4 [43]. We expected that a ligand pose that formed stable binding with TMPRSS2 should have very limited movement most of the time, e.g., within the vicinity of the original position. We observed that, for all the protein–ligand complexes and the apo-form TMPRSS2, the protein approached equilibrium very quickly, with an RMSD of about 2 to 3 Å (Figure S4), indicating that the TMPRSS2 catalytic domain is quite stable with or without a ligand. In most cases, ligand binding could induce a slightly lower protein RMSD (Figure S4), suggesting that protein–ligand interactions may further enhance the protein’s stability. The root mean square fluctuations (RMSFs) were, in general, not more than 3 Å for the nonterminal amino acids (Figure S5), suggesting a high rigidity of the protein. The RMSF profiles did not change much with or without ligand binding (Figure S5). Only a handful of residues were shown to have higher flexibility (e.g., > 3 Å), including Met320, Phe321, Phe357, Lys390, Asn450, and the C-terminal Gly492. All of the flexible residues were located on the loop regions that are distant from the binding pocket. Therefore, we reasoned that their flexibility would not have a great influence on ligand binding.

Each of the 13 ligands starting from different poses had, in general, much larger RMSD fluctuations compared with that of the protein (Figure S6), indicating that the ligands are more mobile. To quantify the mobility of the ligands, we calculated the mean and median RMSD for each ligand pose across the whole MD process (Table S2). Most of the drugs had one or more poses with a relatively low mean and median RMSD, e.g., <3.5 Å in the 100-ns dynamics process (Table 3). Pose 4 of Nafamostat had the lowest median RMSD of 2.1 Å, suggesting that the pose was bound to TMPRSS2 in an extremely stable manner for at least half of the simulation time. Pose 4 of Camostat also achieved a stable binding with a median RMSD of 2.9 Å. Most drugs exhibited a lower mobility than Camostat in terms of the mean and median RMSDs, suggesting a stabilized binding pose (Table 3). Note that pose 3 of Otamixaban and pose 1 of Propamidine also achieved a low median RMSD of 2.1 Å, the same as pose 4 of Nafamostat. Only two drugs, PCI-27483 (DB13000) and Dabigatran (DB14726), exhibited a median RMSD of >3.5 Å for all the poses investigated (Table S2). Surprisingly, four control molecules (CID2442, CID2882138, CID5395514, and CID765269) also had at least one stable pose, with mean and median RMSDs not more than 3.5 Å (Table S2). Therefore, the ligand RMSD alone was not sufficiently reliable to distinguish the strong inhibitors (e.g., Nafamostat and Camostat) and weak binders (e.g., the control compounds).

**Figure 6:**
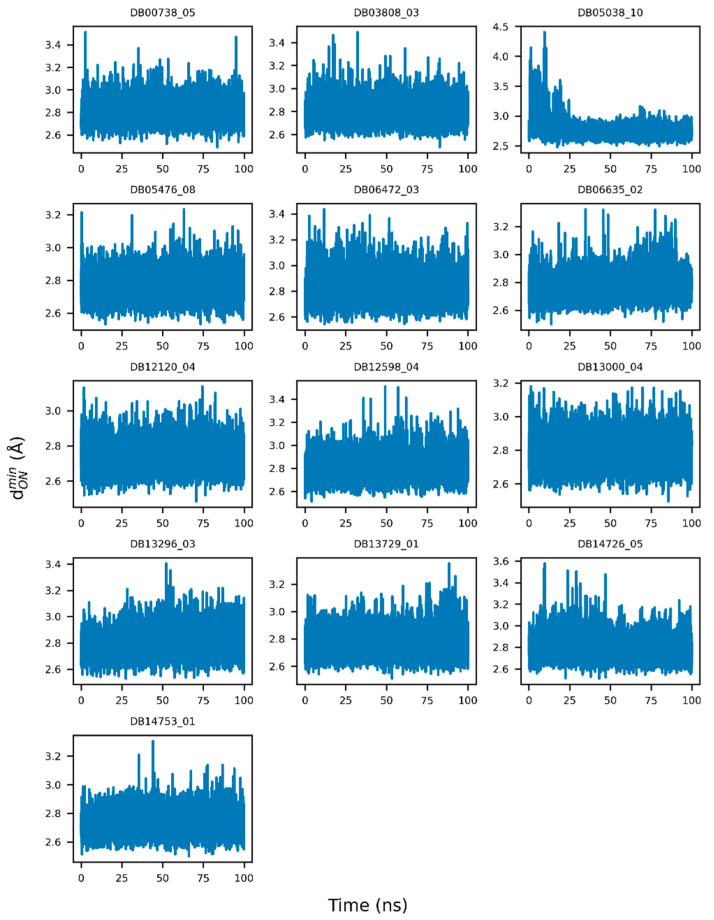
Example illustration of the variations of the minimum distance between guanidinobenzoyl or aminidinobenzoyl and the Asp435 carboxyl ($d_{ON}^{min}$) over a 100-ns simulation time.

According to docking models, the guanidinobenzoyl or aminidinobenzoyl groups were docked into the deep S1 pocket and formed salt bridge interactions with Asp435, while the other parts of the ligands were accessible to the bulk solvent. Therefore, the large ligand RMSDs could be partly due to the swing of the non-buried portion. We further examined the stability of the putative salt bridge interactions by measuring the minimum distance between the positively charged guanidinobenzoyl or aminidinobenzoyl group and the negatively charged carboxyl group of Asp435 (denoted as $d_{ON}^{min}$); only the distances between the nitrogen and oxygen atoms were calculated. We carried out this analysis, because a large RMSD of the whole ligand did not necessarily mean the salt bridges were broken. All the drugs had at least one pose with a mean and median $d_{ON}^{min}$ fluctuating around 2.8 Å, an ideal salt bridge distance, with small deviations (Figure 6 and Figure S7 and Table S3). Therefore, a long-time MD simulation indicated the high stability of the putative salt bridge interactions between the guanidinobenzoyl or aminidinobenzoyl group and Asp435, which should be important for TMPRSS2 inhibition. We also calculated the minimum distance between their nonhydrogen atoms and the carboxyl group of Asp435 (denoted as $d_{OD1/OD2}^{min}$) for the control compounds (Table S3 and Figure S7). As shown in Table S3, almost all the poses of these compounds had a long $d_{OD1/OD2}^{min}$ distance, suggesting that these molecules might not be deeply docked in the S1 pocket, which may partly explain their weak inhibition of TMPRSS2. Only pose 1 of compound CID5395514 exhibited a short mean and median $d_{OD1/OD2}^{min}$ distance (~2.6 Å) to Asp435 (Figure S7), which was because the phenolic hydroxyl group of CID5395514 formed a hydrogen bond with the carboxyl group of Asp435.

It should be noted that the poses with the lowest RMSD values and the smallest deviations of the $d_{ON}^{min}$ distance were not always those with the best LeDock and/or EvoEF2 scores. In fact, only pose 10 of DB05038 and pose 1 of DB05476 satisfied this description. However, most of the poses with the lowest RMSD values and the smallest deviations of the $d_{ON}^{min}$ distance were ranked in the top 3, and all such poses were always ranked in the top 7 by EvoEF2. Therefore, it was reasonable to perform MD simulations with the top 10 poses picked up by EvoEF2 to cover as many good poses as possible.

### 2.5. MM/GBSA-Binding Free Energy Assessment Suggests High Stability of Binding

The docking scores suggested that the proposed drugs had generally higher binding affinity (i.e., lower docking scores) to TMPRSS2 than the control molecules (Table S1). Based on the MD trajectories, we further calculated the binding free energy between TMPRSS2 and the drugs/control compounds using the MM/GBSA approach (Table 4 and Table S4). Technically, given an MD trajectory, a binding free energy ($\Delta G_{bind}$) could be calculated from a few MD frames. Thus, individual binding free energy values could be obtained from the simulations performed on different docked poses. In this regard, the final MM/GBSA-binding free energy was taken as the $\Delta G_{bind}$ calculated for the most stable pose by considering the magnitude of the ligand RMSD and its fluctuations (Table S2 and Figure S6), the stability of the $d_{ON}^{min}$ or $d_{OD1/OD2}^{min}$ distances (Figure S7), and the stability of the MD trajectory in the last 10 ns (Figure S6). The binding free energy for the five control molecules, i.e., CID2442, CID2882138, CID5395514, CID693919, and CID765269, were −20.60, −21.00, −25.61, −25.67, and −12.37 kcal/mol, respectively, which were generally higher than those calculated for the 13 proposed drugs (Table 4). A two-tailed Student’s *t*-test showed that the binding free energies for the proposed drugs and control compounds were significantly different with a *p*-value = 0.02. Therefore, we reasoned that the guanidinobenzoyl- and aminidinobenzoyl-containing drugs may exhibit high stability in binding to TMPRSS2.

To further elucidate the role of specific amino acids in the protein–ligand interactions, we performed a per-residue binding free energy decomposition analysis using the gmx_MMPBSA [44] program. As shown in Table 5, each drug was shown to form extensive binding interactions with the residues in the S1 pocket, including Asp435, Ser436, Cys437, Gln438, Thr459, Ser460, Trp461, Gly462, Ser463, Gly464, Cys465, and/or Tyr474. Most drugs, except DB06635, DB12120, and DB14723, also formed favorable interactions with two out of the three catalytic triad residues (i.e., Ser441 and His296).

**Figure 7:**
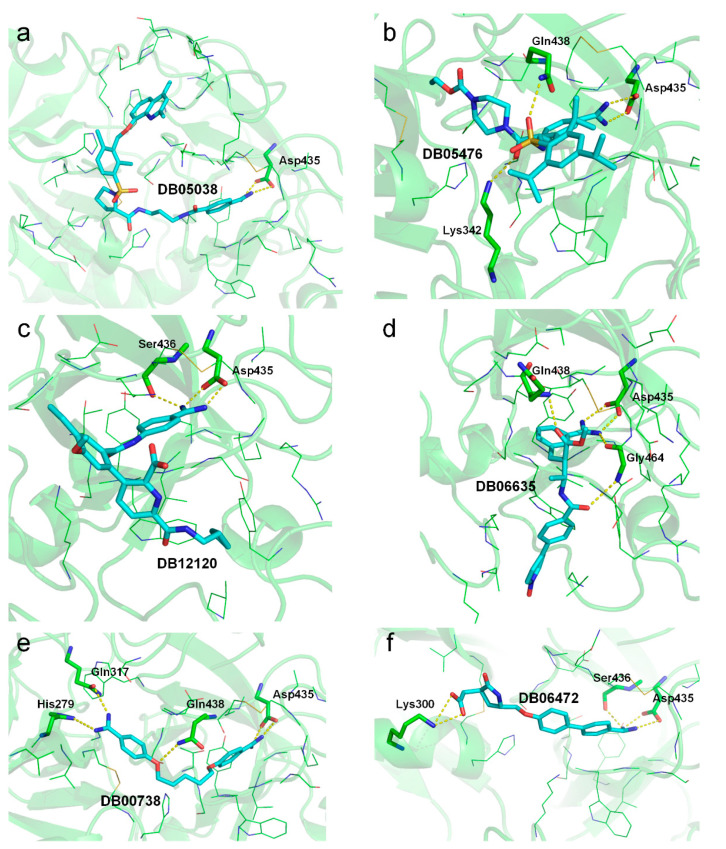
Salt bridge and hydrogen-bonding interactions for representative drugs at the end of the 100-ns MD simulations. (**a**) DB05038. (**b**) DB05476. (**c**) DB12120. (**d**) DB06635. (**e**) DB00738. (**f**) DB06472. Ligands and residues that are involved in hydrogen bonds or salt bridges (marked in yellow dashed lines) are shown in cyan and green sticks, respectively. Other binding residues are shown in lines.

Based on the relative MM/GBSA-binding free energy and decomposed energy terms in Table 4, we were able to give a “prioritization list” among the 11 repurposed drugs, not considering the known inhibitors Nafamosta and Camostat. According to the binding free energy, the top five putative noncovalent binders were Anatibant (DB05038), WX-UK1 (DB05476), Avoralstat (DB12120), Otamixaban (DB06635), and Pentamidine (DB00738). We visualized the binding modes of these drugs at the end of the MD simulations (i.e., the snapshot at 100 ns) in Figure 7; all these drugs still formed bidentate salt bridge interactions with Asp435 (Figure 7a–e). The top binder, DB05038, only formed two salt bridges, and its low binding free energy was probably because it formed abundant van der Waals interactions with the surrounding residues due to its large size and stretched conformation (Figure 7a). The second-best binder, DB05476, formed two extra hydrogen bonds with the side chain of Gln438 and Lys342 each (Figure 7b); this drug also showed good van der Waals interactions (Table 4). Drug DB12120, ranked as third place, formed an extra hydrogen bond with Ser436 in the S1 pocket (Figure 7c). Drugs DB06635 and DB00738, which were ranked fourth and fifth, formed a few extra hydrogen bonds with the surrounding residues (Figure 7d,e). The drug DB06472 might also be a strong binder due to the extra hydrogen bond with Ser436 and salt bridge interactions with Lys300, although it achieved a relatively high binding free energy of −26.20 kcal/mol (Figure 7f). The other drugs, except DB14753, achieved slightly higher but comparable binding free energies to the top five binders. Their binding modes at the 100-ns MD snapshot also retained the salt bridge interactions with Asp435 and might form an extra hydrogen bond with the adjacent Ser436 (Figure S8). The “forward” mode of Nafamostat competed for the catalytic Ser441 with His296, whereas the “reverse” mode was involved in a hydrogen-bonding interaction with Lys342. DB13000 formed a hydrogen bond with Lys342 with its sulfone group.

## 3. Discussion

Trypsin preferentially cleaves substrates with an arginine or lysine at the P1 position, because the positively charged guanidine or amino group of the P1 residue specifically recognizes the negatively charged aspartic acid located in the S1 pocket of trypsin through salt bridge interactions. This feature has also been used to design trypsin inhibitors by introducing a positively charged side chain to mimic arginine or lysine. TMPRSS2 contains a trypsin-like extracellular catalytic domain, and therefore, the salt bridging feature may help screen and repurpose existing drugs against TMPRSS2 for COVID-19 treatment.

Consistent with this finding, previous studies suggested that the guanidinobenzoyl and/or aminidinobenzoyl group in Nafamostat, Camostat, or GBPA, a metabolite of Camostat, could form stable binding interactions with the conserved Asp435 in the S1 pocket in TMPRSS2 [18] and result in inhibition [11,18]. Building on these studies, we hypothesized that some other guanidinobenzoyl- or aminidinobenzoyl-containing drugs may also function as potential TMPRSS2 inhibitors. It should be noted that Camostat, Nafamostat, and GBPA are covalent TMPRSS2 inhibitors [18,32], and they share identical acyl-enzyme complexes, but their activities against TMPRSS2 vary dramatically. Shrimp et al. showed that Camostat, GBPA, and Nafamostat inhibited TMPRSS2 with IC_50_ of 6.2, 33.3, and 0.27 nM, respectively [11]. The differences in the TMPRSS2 inhibitions of these three drugs can only arise from either the populations of their Michaelis complexes preceding the covalent acyl-enzyme or the differences in the catalytic rates of acylation [18]. The acylation rates may depend on their leaving group pKas, which are expected to be comparable, since the three drugs are in a similar reactive environment. Through Markov state modeling following extensive MD simulations, Hempel et al. estimated that the populations of the Michaelis complexes for Nafamostat, Camostat, and GBPA had approximate ratios of 6:2:1 [18], suggesting that Nafamostat more readily forms the covalent acyl-enzyme, explaining its higher potency than Camostat and GBPA. Thus, we reasoned that the population of the Michaelis complex, which depends on the TMPRSS2-ligand-binding capability, determines the potency of both covalent and noncovalent inhibitions. For both kinds of inhibitions, the Michaelis complex could be modeled with the molecular docking approach, and its binding stability could be roughly evaluated by MD simulations, as carried out in this work.

Using five weak inhibitors of TMPRSS2 as negative controls, the molecular docking, MD simulation, and MM/GBSA-binding free energy calculation studies suggested that the 13 proposed guanidinobenzoyl- or aminidinobenzoyl-containing drugs, in general, showed markedly higher binding potency to TMPRSS2 by forming stable salt bridge interactions with Asp435 in the S1 pocket together with other favorable forces, such as hydrogen bonds and van der Waals interactions (Table 4 and Table S4 and Figure 7 and Figure S8). The observation built on in silico experiments that guanidinobenzoyl- or aminidinobenzoyl-containing drugs potently inhibit the trypsin-like TMPRSS2 in this study was consistent with previous reports on virtual screening [10] or drug repurposing [28,29]. For example, Huggins identified three top-scoring serine protease inhibitors against TMPRSS2 from DrugBank, i.e., DB03782, DB03213, and DB04107 [28]. DB03782 contains a guanidinobenzoyl group, while DB03213 and DB04107 contain one or more aminidinobenzoyl groups. It should be noted that these drugs were excluded from our study, because we considered only the approved or investigational drugs. Through virtual screening, Hu et al. identified three aminidinobenzoyl-containing drugs from the database, i.e., NCGC00378763, NCGC00522422, and NCGC00386945, and the fluorogenic biochemical assay suggested that the three drugs were able to inhibit TMPRSS2 with IC_50_ of 0.62, 2.2, and 0.88 µM, respectively [10]. Interestingly, NCGC00378763 and NCGC00522422 are the drugs Otamixaban and WX-UK1 evaluated in this work. NCGC00386945 was not included here, because it is an experimental drug. Collectively, we strongly believe that the 11 drugs, except Camostat and Nafamostat, evaluated in this study are promising noncovalent TMPRSS2 inhibitors, and their potency for COVID-19 treatment could be further investigated. Additionally, it is feasible to optimize these drugs against TMPRSS2 due to their diverse scaffolds.

## 4. Materials and Methods

### 4.1. TMPRSS2 Structural Modeling and Model Quality Assessment

The structure model for the TMPRSS2 catalytic domain (amino acids 256-492) was constructed using the deep-learning contact-guided protein structure prediction approach, C-I-TASSER [23]. The global quality of the model was inherently assessed by C-I-TASSER by reporting the C-score [24] and estimated TM-score [25], a descriptor to tell if the structure was predicted to be in a correct fold. The model’s local quality was evaluated using MolProbity [26]. The catalytic elements, such as the Ser-His-Asp triad and oxyanion holes, were manually inspected after model visualization.

### 4.2. Drug Library Construction

We searched DrugBank [21] for approved or clinically investigational drugs using guanidinobenzoyl or aminidinobenzoyl as a substructure (Figure S1). In total, 13 drugs were identified, including Camostat and Nafamostat.

### 4.3. Molecular Docking and Pose Reranking

The initial 3D drug structures were downloaded from DrugBank [21], PDBe [45], or ChemSpider [46]. We then used Open Babel v3.1.1 [47] to add ligand hydrogen atoms at pH 7.0 and convert the ligand files into the mol2 format with default parameters. The molecular docking tool LeDock [37] was used to dock the drugs into the putative binding pocket of TMPRSS2. The docking box for defining the binding pocket was determined using AutoDockTools v1.5.6 [48]; the lower and upper bounds of the binding pocket in Cartesian coordinates were set as (−10, −16, −4) and (18, 12, 24), respectively. The docking poses were rescored and reranked using a physics- and knowledge-based potential EvoEF2 [39].

### 4.4. MD Simulation

Molecular dynamics simulations were performed using GROMACS v2020.4 [43] with the Amber force field. Specifically, the Amber ff03 force field [49] was used for proteins, while the general Amber force field [50] was used for ligands. The ligand topologies, parameters, and coordinates in GROMACS format were prepared using ACPYPE [40] with the AM1-BCC [41,42] charge model. In each simulation, a cubic box was constructed with a distance of 10 Å from the solute and filled with TIP3P water molecules. The system was neutralized by the addition of an appropriate number of Na^+^ or Cl^−^ ions. After the system was constructed, energy minimization was carried out using the steepest descent minimization with a maximum force of 10 kJ/mol. The system was equilibrated by a 100-ps NVT simulation at 310 K, followed by a 100-ps NPT process at 1 bar with position restraints (1000 kJ/mol) on the heavy atoms of the protein and ligand. Next, unconstrained production MD was carried out at 310 K and 1 bar for 100 ns. The LINCS [51] algorithm was utilized to restrain the hydrogen bonds. Nonbonded interactions were truncated at 12 Å, and the Particle Mesh Ewald [52] method was used for long-range electrostatic interactions. The velocity-rescaling thermostat [53] and Parrinello-Rahman barostat [54] were used for temperature and pressure coupling, respectively. The production MD simulation trajectory was saved with a time step of 10 ps, and thus, 10,001 frames were created. The GROMACS built-in commands *rmsd*, *rmsf*, and *mindist* were used for analyzing the MD simulation trajectories.

### 4.5. MM/GBSA-Binding Free Energy Calculation and Per-Residue Energy Decomposition

The binding free energy ($\Delta G_{bind}$) was calculated by:

(1) $$\Delta G_{bind}=G_{complex}-\left(G_{protein}+G_{ligand}\right)=\Delta H-T\Delta S$$

where ΔH and −TΔS represent the enthalpy and entropy contributions to the binding free energy, respectively. The −TΔS term can be estimated by the interaction entropy approach.

ΔH is calculated as:

(2) $$\Delta H=\Delta E_{MM}+\Delta G_{GB}+\Delta G_{SA}$$

where $\Delta E_{MM}$ represents the gas-phase molecular mechanics energy, $\Delta G_{GB}$ represents the polar solvation free energy, and $\Delta G_{SA}$ represents the nonpolar solvation free energy.

The $\Delta E_{MM}$ term is calculated as:

(3) $$\Delta E_{MM}=\Delta E_{bond}+\Delta E_{angle}+\Delta E_{dihedral}+\Delta E_{vdW}+\Delta E_{ele}$$

where $\Delta E_{bond}$, $\Delta E_{angle},$ and $\Delta E_{dihedral}$ are bonded internal energy terms, and they cancel out for fixed geometries before and after binding, and $\Delta E_{vdW}$ and $\Delta E_{ele}$ are the nonbonded van der Waals and electrostatic interaction energy, respectively.

The binding free energy calculation was performed using the gmx_MMPBSA [44] package. The calculation was carried out on 101 evenly distributed snapshots extracted from the production MD trajectory between 90 and 100 ns. The dielectric constant of the solute, temperature, and salt concentration were set to 2, 310 K, and 0.15 M, respectively, and the other default parameters were used. The per-residue energy decomposition analysis was also carried out using gmx_MMPBSA together with binding free energy calculations with the default parameters.

## 5. Conclusions

Building on the recent finding that the positively charged groups in Camostat and Nafamostat play a critical role in inhibiting TMPRSS2 by stable binding with the conserved aspartic acid Asp435 in the S1 pocket of TMPRSS2, we identified a narrowed set of 13 compounds (three FDA-approved and 10 investigational drugs) with positively charged guanidinobenzoyl or aminidinobenzoyl groups and computationally assessed their potency for inhibiting TMPRSS2. This work differed from virtual screening studies that focus on identifying TMPRSS2 inhibitors from huge drug databases. Usually, a virtual screening study suggests a long list of candidates for experimental tests but, finally, comes up with few positive hits; instead, here, we tried to evaluate and repurpose only a few very promising candidates. The molecular docking studies showed that all the 13 drugs indeed utilized guanidinobenzoyl or aminidinobenzoyl to form favorable salt bridge interactions with the Asp435 carboxyl, and a series of long-time (100 ns) MD simulations revealed the high stability of the salt bridge interactions between each drug and TMPRSS2, although each whole ligand may undergo large conformational changes. The strong binding interactions between TMPRSS2 and the proposed drugs were also supported by the MM/GBSA-binding free energy assessment. Collectively, the computational data supported these drugs as potential TMPRSS2 inhibitors for treating COVID-19.

## Figures and Tables

**Table 1:** The 13 guanidinobenzoyl- or aminidinobenzoyl-containing drugs studied in this work.

DrugBank ID	Drug Name	Status with FDA	Primary Indication
DB00738	Pentamidine	Approved	For the treatment of pneumocystis pneumonia
DB03808	Hexamidine	Approved	For the treatment of acanthamoebiasis
DB05038	Anatibant	Investigational	For the treatment of traumatic brain injuries
DB05476	WX-UK1	Investigational	For the treatment in solid tumors
DB06472	Fradafiban	Investigational	For the treatment in angina
DB06635	Otamixaban	Investigational	For the treatment of thrombosis
DB12120	Avoralstat	Investigational	For the prevention of hereditary angioedema
DB12598	Nafamostat	Investigational	Used as an anticoagulant in patients with disseminative blood vessel coagulation, hemorrhagic lesions, and hemorrhagic tendencies
DB13000	PCI-27483	Investigational	For the treatment of pancreatic cancer, ductal adrenocarcinoma, and exocrine pancreatic cancer
DB13296	Propamidine	Investigational	For the treatment of Acanthamoeba infection
DB13729	Camostat	Investigational	For the treatment of chronic pancreatitis and drug-induced lung injury
DB14726	Dabigatran	Investigational	For the treatment and prevention of blood clots and prevention of stroke in people with atrial fibrillation
DB14753	Hydroxystilbamidine	Approved	For the treatment of nonprogressive blastomycosis of the skin and other mycoses

Note: The table is ranked by DrugBank ID. Camostat and Nafamostat have been approved in Japan but have not been approved by the FDA.

**Table 2:** The best LeDock-binding score and the best EvoEF2 reranking score for each drug.

DrugBank ID	Drug Name	Number of Poses by LeDock	Best Score	
			LeDock (kcal/mol)	EvoEF2 (EEU)
DB00738	Pentamidine	12	−8.73	−37.36
DB03808	Hexamidine	15	−8.47	−35.08
DB05038	Anatibant	18	−8.60	−34.21
DB05476	WX-UK1	17	−8.57	−39.29
DB06472	Fradafiban	8	−7.32	−30.48
DB06635	Otamixaban	17	−8.50	−40.43
DB12120	Avoralstat	7	−9.35	−46.30
DB12598	Nafamostat	6	−8.61	−34.70
DB13000	PCI-27483	16	−9.44	−36.84
DB13296	Propamidine	14	−7.93	−32.48
DB13729	Camostat	13	−8.02	−27.02
DB14726	Dabigatran	15	−9.51	−42.58
DB14753	Hydroxystilbamidine	4	−7.56	−32.56

**Table 3:** The drug poses with both mean and median ligand RMSDs below 3.5 Å.

Drug Name	DrugBank ID_pose	Ligand RMSD (Å)	
		Mean ± std	Median
Pentamidine	DB00738_05	3.2 ± 1.0	3.1
	DB00738_10	2.7 ± 1.6	2.2
Hexamidine	DB03808_03	3.3 ± 1.4	2.7
Anatibant	DB05038_10	3.3 ± 0.6	3.1
WX-UK1	DB05476_01	2.7 ± 0.3	2.6
	DB05476_15	2.8 ± 0.3	2.7
Fradafiban	DB06472_03	3.2 ± 1.4	2.9
Otamixaban	DB06635_02	3.1 ± 0.4	3.1
	DB06635_03	2.5 ± 1.6	2.1
	DB06635_04	2.9 ± 0.7	2.7
	DB06635_05	2.6 ± 0.5	2.7
Avoralstat	DB12120_04	2.9 ± 0.6	2.8
	DB12120_05	2.9 ± 0.7	2.7
Nafamostat	DB12598_01	3.3 ± 0.7	3.2
	DB12598_04	2.1 ± 0.5	2.1
	DB12598_05	2.8 ± 0.7	2.6
	DB12598_06	3.3 ± 0.7	3.4
Propamidine	DB13296_01	2.3 ± 0.7	2.1
	DB13296_06	2.3 ± 0.7	2.2
	DB13296_09	2.5 ± 0.6	2.4
	DB13296_13	3.5 ± 2.4	2.3
Camostat	DB13729_04	3.1 ± 0.6	2.9
Hydroxystilbamidine	DB14753_02	2.9 ± 1.0	3.0
	DB14753_04	2.6 ± 0.7	2.4

**Table 4:** MM/GBSA-binding free energy for the 13 drugs bound to TMPRSS2.

DrugBank ID_pose	$\Delta E_{vdW}$ (kcal/mol)	$\Delta E_{ele}$ (kcal/mol)	$\Delta G_{GB}$ (kcal/mol)	$\Delta G_{SA}$ (kcal/mol)	−TΔS (kcal/mol)	$\Delta G_{bind}$ (kcal/mol)
DB00738_10	−39.95 ± 3.97	−45.66 ± 3.95	43.74 ± 3.42	−5.63 ± 0.34	12.64	−34.86 ± 3.96
DB03808_03	−39.57 ± 3.60	−37.65 ± 4.17	38.21 ± 3.81	−5.25 ± 0.28	13.40	−30.86 ± 3.38
DB05038_10	−56.66 ± 4.21	−25.38 ± 7.13	34.16 ± 6.26	−6.52 ± 0.41	13.62	−40.77 ± 4.06
DB05476_01	−45.72 ± 5.58	−61.61 ± 4.81	64.33 ± 3.68	−5.97 ± 0.60	9.73	−39.23 ± 4.77
DB06472_03	−35.72 ± 5.38	−24.09 ± 6.50	25.59 ± 5.83	−4.21 ± 0.51	12.23	−26.20 ± 5.19
DB06635_05	−44.42 ± 3.83	−53.98 ± 5.26	59.27 ± 4.07	−5.52 ± 0.34	9.35	−35.30 ± 2.95
DB12120_04	−41.16 ± 4.80	−23.86 ± 3.92	25.42 ± 2.90	−5.23 ± 0.36	8.11	−36.71 ± 3.97
DB12598_04	−37.96 ± 3.40	−39.78 ± 4.44	39.03 ± 4.10	−4.72 ± 0.25	12.61	−30.83 ± 3.33
DB13000_04	−43.08 ± 6.34	−35.35 ± 7.21	34.19 ± 6.28	−5.21 ± 0.60	19.63	−29.82 ± 6.29
DB13296_06	−37.30 ± 3.13	−40.16 ± 3.87	39.34 ± 3.42	−5.12 ± 0.21	10.75	−32.49 ± 2.77
DB13729_04	−41.84 ± 3.21	−49.83 ± 6.21	54.96 ± 5.21	−5.27 ± 0.35	11.56	−30.42 ± 3.02
DB14726_08	−37.90 ± 4.14	−31.49 ± 4.79	31.87 ± 3.85	−4.81 ± 0.42	11.68	−30.65 ± 3.61
DB14753_04	−25.85 ± 4.42	−42.78 ± 4.90	39.32 ± 4.67	−3.67 ± 0.35	18.06	−14.92 ± 3.54

**Table 5:** Residues with a binding energy contribution of <−0.5 kcal/mol calculated by per-residue energy decomposition.

DrugBank ID	Residues (Per-Residue Energy in kcal/mol)
DB00738	His279 (−0.95), Val280 (−0.82), Cys281 (−0.58), ***His296 (−1.21)***, Cys297 (−0.77), Thr393 (−0.59), **Asp435 (−1.64)**, **Ser436 (−1.45)**, **Cys437 (−1.77)**, **Gln438 (−2.41)**, Gly439 (−1.60), Asp440 (−0.82), ***Ser441 (−1.22)***, **Thr459 (−0.59)**, **Ser460 (−0.55)**, **Trp461 (−1.83)**, **Gly462 (−0.88)**, **Cys465 (−0.67)**, **Tyr474 (−0.54)**
DB03808	His279 (−0.50), Val280 (−1.62), Cys281 (−0.62), ***His296 (−1.59)***, Cys297 (−0.97), **Asp435 (−1.52)**, **Ser436 (−1.52)**, **Cys437 (−1.72)**, **Gln438 (−2.08)**, Asp440 (−0.57), ***Ser441 (−0.97)***, **Thr459 (−0.69)**, **Trp461 (−1.68)**, **Gly462 (−0.94)**, **Ser463 (−0.54)**, **Cys465 (−0.64)**
DB05038	His279 (−0.55), Val280 (−0.84), Cys281 (−0.73), ***His296 (−1.26)***, Cys297 (−0.70), Lys342 (−0.77), Thr393 (−0.85), **Asp435 (−2.33)**, **Ser436 (−2.87)**, **Cys437 (−1.74)**, **Gln438 (−2.55)**, Gly439 (−1.55), Asp440 (−0.75), ***Ser441 (−0.94)***, **Trp461 (−1.05)**, **Gly462 (−1.19)**, **Ser463 (−0.86)**, **Cys465 (−0.97)**
DB05476	Val280 (−0.61), ***His296 (−1.08)***, **Asp435 (−2.98)**, **Ser436 (−0.78)**, **Cys437 (−1.44)**, **Gln438 (−2.41)**, Gly439 (−0.89), Asp440 (−0.65), ***Ser441 (−1.45)***, **Trp461 (−2.70)**, **Gly462 (−0.97)**, **Ser463 (−2.65)**, **Gly464 (−1.91)**
DB06472	Val280 (−0.80), ***His296 (−1.04)***, Cys297 (−0.54), **Asp435 (−1.31)**, **Ser436 (−1.42)**, **Cys437 (−2.00)**, **Gln438 (−2.35)**, Gly439 (−0.90), Asp440 (−0.85), ***Ser441 (−0.94)***, **Thr459 (−0.60)**, **Trp461 (−1.19)**, **Gly462 (−0.90)**, **Ser463 (−0.64)**, **Cys465 (−0.85)**
DB06635	Thr341 (−1.06), Lys342 (−0.60), Leu419 (−1.52), **Asp435 (−3.64)**, **Cys437 (−1.56)**, **Gln438 (−1.68)**, Asp440 (−0.64), **Thr459 (−0.51)**, **Trp461 (−5.00)**, **Gly462 (−1.53)**, **Ser463 (−1.43)**, **Gly464 (−1.22)**
DB12120	Lys342 (−0.65), Tyr416 (−0.60), **Asp435 (−1.93)**, **Ser436 (−0.86)**, **Cys437 (−1.82)**, **Gln438 (−3.67)**, **Thr459 (−0.58)**, **Trp461 (−3.70)**, **Gly462 (−2.26)**, **Ser463 (−1.89)**, **Gly464 (−0.53)**, **Cys465 (−0.65)**
DB12598	Val280 (−0.90), Cys281 (−0.59), ***His296 (−1.73)***, Cys297 (−0.85), **Asp435 (−1.73)**, **Ser436 (−1.54)**, **Cys437 (−2.11)**, **Gln438 (−2.48)**, Asp440 (−0.96), ***Ser441 (−1.31)***, **Thr459 (−0.60)**, **Trp461 (−1.40)**, **Gly462 (−0.95)**, **Ser463 (−0.91)**, **Cys465 (−0.64)**
DB13000	***His296 (−0.84)***, **Asp435 (−0.71)**, **Ser436 (−1.22)**, **Cys437 (−2.18)**, **Gln438 (−4.36)**, Gly439 (−1.32), Asp440 (−0.55), ***Ser441 (−1.10)***, **Thr459 (−0.58)**, **Trp461 (−1.69)**, **Gly462 (−1.44)**, **Ser463 (−1.58)**, **Gly464 (−0.54)**, **Cys465 (−0.91)**, Pro471 (−0.60)
DB13296	His279 (−0.93), Val280 (−0.82), Cys281 (−0.80), ***His296 (−0.64)***, **Asp435 (−1.65)**, **Ser436 (−1.30)**, **Cys437 (−1.99)**, **Gln438 (−2.04)**, Gly439 (−1.49), Asp440 (−0.99), ***Ser441 (−1.25)***, **Thr459 (−0.64)**, **Trp461 (−1.58)**, **Gly462 (−0.97)**, **Cys465 (−0.56)**
DB13729	Val278 (−0.66), Val280 (−2.04), Cys281 (−0.74), ***His296 (−1.05)***, Cys297 (−0.70), **Asp435 (−2.58)**, **Ser436 (−0.69)**, **Cys437 (−1.62)**, **Gln438 (−1.40)**, Gly439 (−1.04), Asp440 (−0.95), ***Ser441 (−1.50)***, **Thr459 (−0.69)**, **Trp461 (−1.10)**, **Gly462 (−0.72)**, **Gly464 (−0.84)**
DB14726	**Asp435 (−3.07)**, **Ser436 (−0.76)**, **Cys437 (−2.02)**, **Gln438 (−3.05)**, Asp440 (−0.57), **Thr459 (−0.64)**, **Trp461 (−1.44)**, **Gly462 (−1.63)**, **Ser463 (−1.25)**, **Gly464 (−0.52)**, **Cys465 (−1.24)**, Val473 (−0.76), **Tyr474 (−1.94)**
DB14753	***His296 (−0.97)***, **Asp435 (−2.48)**, **Ser436 (−1.25)**, **Cys437 (−2.10)**, **Gln438 (−1.98)**, Gly439 (−0.67), Asp440 (−0.77), ***Ser441 (−0.69)***, **Thr459 (−0.58)**, **Trp461 (−1.44)**, **Gly462 (−1.13)**, **Gly464 (−0.66)**, **Cys465 (−0.79)**

Note: The per-residue energy decomposition analysis was performed for residues within 6 Å to the initially docked poses based on the MD frames used for MM/GBSA-binding free energy calculations. Catalytic triad residues (His296, Asp345, and Ser441) are shown in bold and italic. Binding residues in the S1 pocket are shown in bold.

## Data Availability

The PDB coordinates of the high-quality C-I-TASSER model for the TMPRSS2 catalytic domain, the simplified molecular-input line-entry system (SMILES) [55] files for the 13 drugs, the ligand mol2 files for molecular docking and MD simulations, and the PDB coordinates of the LeDock docked poses are available at https://github.com/tommyhuangthu/tmprss2.

## Supplementary Materials

The following are available online at https://www.mdpi.com/article/10.3390/ijms22137060/s1.

## Abbreviations

ACE2	angiotensin-converting enzyme 2
COVID-19	coronavirus disease 2019
EEU	EvoEF2 energy unit
FDA	United States Food and Drug Administration
GBPA	4-(4-guanidinobenzoyloxy)phenylacetic acid
MD	molecular dynamics
MERS-CoV	Middle East respiratory syndrome coronavirus
MM/GBSA	molecular mechanics/generalized Born surface area
PDB	Protein Data Bank
RMSD	root mean square deviation
RMSF	root mean square fluctuation
SARS-CoV	severe acute respiratory syndrome coronavirus
SARS-CoV-2	severe acute respiratory syndrome coronavirus 2
SMILES	simplified molecular-input line-entry system
TMPRSS2	transmembrane protease serine 2
