# Supplementary material for: Identification of 13 Guanidinobenzoyl- or Aminidinobenzoyl-Containing Drugs to Potentially Inhibit TMPRSS2 for COVID-19 Treatment
Source: Int J Mol Sci. 2021 Jun 30;22(13):7060. doi: 10.3390/ijms22137060 (PMC8269196; doi:10.3390/ijms22137060)
Supplement: Supplementary file 1 [file ijms-22-07060-s001.zip › ijms-1253522-supplementary.pdf]

Supporting Information

Identification of 13 guanidinobenzoyl- or aminidinobenzoyl-containing drugs to potentially inhibit TMPRSS2 for COVID-19 treatment

Xiaoqiang Huang[1], Robin Pearce[1], Gilbert S. Omenn[1,2], and Yang Zhang[1,3]*

[1]Department of Computational Medicine and Bioinformatics, [2]Departments of Internal Medicine and Human Genetics and School of Public Health, [3]Department of Biological Chemistry, University of Michigan, 100 Washtenaw Avenue, Ann Arbor, MI 48109, USA

* To whom correspondence should be addressed. Email: zhng@umich.edu

Table of Contents

Supporting Tables

Table S1. LeDock/EvoEF2 scores and the formation of salt-bridge interactions with Asp435 for all the poses of each compound.

Table S2. Mean and median ligand RMSD calculated from 100 ns molecular dynamics simulations for each compound with different poses.

Table S3. Mean and median d_{ON}^{min} distance calculated from 100 ns molecular dynamics simulations for each compound with different poses.

Table S4. MM/GBSA binding free energy for the control compounds bound to TMPRSS2.

Supporting Figures

Figure S1. The workflow for searching and narrowing down the hit list of guanidinobenzoyl- or aminidinobenzoyl-containing drugs.

Figure S2. Summary of the statistics for the MolProbity assessment of the TMPRSS2 model.

Figure S3. Five weak TMPRSS2 inhibitors used as control compounds in this study.

Figure S4. RMSD of TMPRSS2 calculated from 100 ns molecular dynamics simulations for each compound with different poses.

Figure S5. RMSF of TMPRSS2 residues calculated from 100 ns molecular dynamics simulations for each compound with different poses.

Figure S6. Ligand RMSD calculated from 100 ns molecular dynamics simulations for each compound with different poses.

Figure S7. The d_{ON}^{min} distance calculated from 100 ns molecular dynamics simulation for each compound with different poses.

Figure S8. Salt-bridge and hydrogen-bonding interactions for other repurposed drugs at the end of the 100-ns MD simulations.

Supporting Tables

Table S1. LeDock/EvoEF2 scores and the formation of salt-bridge interactions with Asp435 for all the poses of each compound.

Compound ID	Name	LeDock		EvoEF2		Salt-bridge interactions with Asp435
		Pose index	Score (kcal/mol)	Rank	Score (EEU)	
CID2442	Bromohexine	**1**	**−5.75**	2	−18.28	No
		2	−5.73	3	−14.15	No
		3	−5.68	5	−6.664	No
		4	−5.40	4	−13.07	No
		5	−5.37	**1**	**−21.56**	No
CID2882138	4401-0077	**1**	**−4.77**	**1**	**−17.44**	No
		2	−4.44	2	−11.35	No
CID5395514	4554-5138	**1**	**−5.41**	**1**	**−23.78**	No
		2	−5.06	2	−19.51	No
CID693919	8008-1235	**1**	**−5.11**	3	49.36	No
		2	−4.90	**1**	**−20.33**	No
		3	−4.24	2	−16.06	No
CID765269	0591-5329	**1**	**−4.97**	3	−20.81	No
		2	−4.80	5	−20.01	No
		3	−4.79	7	−18.76	No
		4	−4.76	4	−20.37	No
		5	−4.74	8	−18.75	No
		6	−4.73	**1**	**−21.97**	No
		7	−4.70	2	−21.03	No
		8	−4.62	10	−17.38	No
		9	−4.53	6	−19.20	No
		10	−4.49	9	−18.28	No
DB00738	Pentamidine	**1**	**−8.73**	6	−30.07	Yes
		2	−8.35	12	−23.14	Yes
		3	−8.24	5	−30.57	Yes
		4	−8.03	**1**	**−37.36**	Yes
		5	−8.01	4	−31.15	Yes
		6	−7.72	11	−24.09	Yes
		7	−7.68	3	−31.48	Yes
		8	−7.61	8	−28.07	Yes
		9	−7.57	9	−25.58	Yes
		10	−7.42	2	−32.47	Yes
		11	−7.42	10	−24.40	Yes
		12	−7.36	7	−30.02	Yes
DB03808	Hexamidine	**1**	**−8.47**	2	−34.81	Yes
		2	−8.35	4	−33.87	Yes
		3	−8.20	7	−31.46	Yes
		4	−8.01	8	−30.12	Yes
		5	−7.86	6	−32.97	Yes
		6	−7.77	3	−34.19	Yes
		7	−7.76	9	−29.09	Yes
		8	−7.68	5	−33.44	Yes
		9	−7.66	**1**	**−35.08**	Yes
		10	−7.16	10	−28.29	Yes
		11	−7.08	14	−25.84	Yes
		12	−7.06	11	−27.90	Yes
		13	−7.04	13	−25.92	Yes
		14	−7.03	12	−26.18	No
		15	−6.76	15	−12.06	Yes

DB05038	Anatibant	**1**	**−8.60**	17	−10.34	No
		2	−8.21	12	−22.46	No
		3	−7.87	15	−15.26	No
		4	−7.86	5	−27.57	No
		5	−7.78	2	−33.59	No
		6	−7.74	14	−22.10	No
		7	−7.67	13	−22.28	No
		8	−7.41	9	−24.81	No
		9	−7.34	6	−27.26	No
		10	−7.16	**1**	**−34.21**	Yes
		11	−7.12	16	−13.87	No
		12	−7.07	18	40.79	No
		13	−6.93	4	−30.38	No
		14	−6.90	10	−24.52	No
		15	−6.85	8	−25.17	No
		16	−6.66	7	−25.46	No
		17	−6.33	11	−24.45	No
		18	−6.30	3	−32.04	No
DB05476	WX-UK1	**1**	**−8.57**	3	−35.36	Yes
		2	−8.35	5	−32.92	Yes
		3	−8.30	15	−12.03	No
		4	−8.11	**1**	**−39.29**	Yes
		5	−7.98	8	−26.64	Yes
		6	−7.79	6	−31.35	Yes
		7	−7.64	2	−35.60	Yes
		8	−7.59	4	−33.36	Yes
		9	−7.44	16	−11.77	Yes
		10	−7.30	10	−21.42	No
		11	−7.17	17	0.01	No
		12	−6.88	11	−21.21	Yes
		13	−6.77	13	−16.44	No
		14	−6.32	7	−26.79	No
		15	−6.29	9	−23.68	No
		16	−6.10	14	−13.01	Yes
		17	−5.58	12	−17.88	No
DB06472	Fradafiban	**1**	**−7.32**	4	−29.48	Yes
		2	−7.16	2	−30.45	Yes
		3	−7.07	3	−29.73	Yes
		4	−6.97	6	−25.54	Yes
		5	−6.80	**1**	**−30.48**	Yes
		6	−6.72	7	−21.49	No
		7	−6.45	8	−20.60	No
		8	−6.34	5	−26.78	Yes
DB06635	Otamixaban	**1**	**−8.50**	3	−37.61	Yes
		2	−7.95	2	−37.94	Yes
		3	−7.70	6	−36.26	Yes
		4	−7.62	8	−29.92	Yes
		5	−7.45	5	−36.99	Yes
		6	−7.34	7	−34.06	Yes
		7	−7.29	**1**	**−40.43**	Yes
		8	−7.26	17	−5.02	Yes
		9	−6.93	10	−27.24	Yes
		10	−6.85	15	−6.45	No
		11	−6.82	9	−28.94	Yes
		12	−6.82	4	−37.37	No
		13	−6.36	11	−24.68	No

		14	−6.26	14	−10.07	No
		15	−6.04	12	−22.66	No
		16	−5.74	13	−19.07	No
		17	−5.25	16	−5.78	Yes
DB12120	Avoralstat	**1**	**−9.35**	**1**	**−46.30**	Yes
		2	−8.77	4	−26.41	Yes
		3	−8.62	2	−36.69	Yes
		4	−8.00	3	−33.56	Yes
		5	−7.53	5	−24.87	Yes
		6	−6.96	6	−24.74	Yes
		7	−6.64	7	−19.98	No
DB12598	Nafamostat	**1**	**−8.61**	**1**	**−34.70**	Yes
		2	−8.46	3	−28.96	Yes
		3	−8.21	4	−24.37	Yes
		4	−8.01	2	−31.60	Yes
		5	−7.57	6	−18.78	Yes
		6	−7.37	5	−20.01	Yes
DB13000	PCI-27483	**1**	**−9.44**	5	−33.42	Yes
		2	−9.33	15	4.12	No
		3	−8.91	13	−19.84	Yes
		4	−8.88	7	−30.30	Yes
		5	−8.86	2	−36.29	Yes
		6	−8.56	8	−28.78	Yes
		7	−8.56	3	−36.25	Yes
		8	−8.48	4	−35.11	No
		9	−8.26	6	−31.17	Yes
		10	−8.18	9	−27.96	Yes
		11	−8.14	11	−25.70	No
		12	−8.14	14	−17.84	Yes
		13	−8.02	**1**	**−36.84**	Yes
		14	−7.89	10	−26.37	No
		15	−7.21	16	22.57	No
		16	−7.07	12	−25.04	No
DB13296	Propamidine	**1**	**−7.93**	4	−29.77	Yes
		2	−7.78	13	−25.08	Yes
		3	−7.70	9	−28.08	Yes
		4	−7.63	6	−29.25	Yes
		5	−7.61	12	−25.19	Yes
		6	−7.52	3	−31.90	Yes
		7	−7.43	10	−26.86	Yes
		8	−7.33	11	−26.76	Yes
		9	−7.33	**1**	**−32.48**	Yes
		10	−7.31	2	−31.95	Yes
		11	−7.14	14	−24.59	No
		12	−7.07	8	−28.61	Yes
		13	−7.02	7	−29.03	Yes
		14	−6.96	5	−29.76	Yes
DB13729	Camostat	**1**	**−8.02**	8	−20.88	Yes
		2	−7.96	**1**	**−27.02**	Yes
		3	−7.82	5	−24.27	Yes
		4	−7.75	2	−26.14	Yes
		5	−7.42	11	−17.33	Yes
		6	−7.41	12	−17.00	Yes
		7	−7.18	9	−19.87	Yes
		8	−7.18	10	−17.81	Yes
		9	−7.16	7	−21.16	Yes

		10	−7.07	6	−22.01	Yes
		11	−6.92	3	−24.69	Yes
		12	−6.15	4	−24.53	No
		13	−6.14	13	−13.52	Yes
DB14726	Dabigatran	**1**	**−9.51**	**1**	**−42.58**	Yes
		2	−8.81	12	−16.41	Yes
		3	−8.41	2	−40.81	Yes
		4	−8.26	15	−10.43	Yes
		5	−8.20	5	−31.28	Yes
		6	−8.14	14	−10.77	Yes
		7	−8.08	4	−31.77	No
		8	−7.90	3	−33.29	Yes
		9	−7.70	9	−26.13	Yes
		10	−7.64	11	−21.25	Yes
		11	−7.64	7	−29.85	No
		12	−7.60	10	−23.65	Yes
		13	−7.51	8	−29.10	Yes
		14	−7.40	13	−11.52	Yes
		15	−7.27	6	−30.84	Yes
DB14753	Hydroxystilbamidine	**1**	**−7.56**	2	−32.18	Yes
		2	−7.27	**1**	**−32.56**	Yes
		3	−7.23	3	−30.49	Yes
		4	−7.08	4	−26.93	Yes

Table S2. Mean and median ligand RMSD calculated from 100 ns molecular dynamics simulations for each compound with different poses.

Compound ID_pose	Ligand RMSD (Å)	
	Mean±std	Median
CID2442_01	7.7±6.2	5.2
CID2442_02	4.5±0.6	4.4
CID2442_03	8.3±6.7	4.5
CID2442_04	2.7±0.5	2.6
CID2442_05	11.0±3.3	10.6
CID2882138_01	3.7±0.6	3.7
CID2882138_02	2.1±0.5	2.2
CID5395514_01	3.4±0.5	3.4
CID5395514_02	19.5±8.3	19.2
CID693919_01	6.3±1.2	6.4
CID693919_02	6.5±0.6	6.5
CID693919_03	12.6±1.4	12.7
CID765269_01	7.7±2.4	7.4
CID765269_02	15.4±0.2	15.2
CID765269_03	9.1±7.9	3.9
CID765269_04	6.0±1.8	5.2
CID765269_05	3.4±0.8	3.2
CID765269_06	16.7±9.4	18.5
CID765269_07	5.8±1.1	5.8
CID765269_08	3.5±0.5	3.5
CID765269_09	6.8±1.4	7.1
CID765269_10	4.3±1.0	4.2
DB00738_01	8.0±3.9	8.6
DB00738_03	7.0±2.9	6.8
DB00738_04	4.7±1.2	5.0
DB00738_05	3.2±1.0	3.1
DB00738_07	4.0±3.2	2.7
DB00738_08	5.9±3.6	3.6
DB00738_09	4.0±1.1	3.6
DB00738_10	2.7±1.6	2.2
DB00738_11	9.3±2.5	10.2
DB00738_12	4.8±0.9	4.8
DB03808_01	4.9±1.6	4.9
DB03808_02	6.8±1.6	7.3
DB03808_03	3.3±1.4	2.7
DB03808_04	8.6±2.2	9.4
DB03808_05	6.1±3.0	5.2
DB03808_06	4.0±1.1	4.3
DB03808_07	4.0±1.1	3.7
DB03808_08	7.3±1.3	7.3
DB03808_09	5.2±1.6	4.6
DB03808_10	7.6±0.9	7.6
DB05038_04	4.8±1.5	4.4
DB05038_05	10.8±2.6	11.5
DB05038_08	7.7±0.8	7.8
DB05038_09	4.2±0.8	4.3
DB05038_10	3.3±0.6	3.1
DB05038_13	6.0±1.5	6.4
DB05038_14	8.9±1.0	8.9
DB05038_15	11.1±1.5	11.2
DB05038_16	11.1±2.7	11.9
DB05038_18	5.0±1.4	4.5

DB05476_01	2.7±0.3	2.6
DB05476_02	5.0±0.8	5.0
DB05476_05	5.5±0.9	5.7
DB05476_06	3.9±0.6	3.8
DB05476_07	3.7±1.0	3.6
DB05476_08	6.5±0.5	6.5
DB05476_10	4.4±0.6	4.5
DB05476_14	6.3±1.5	6.5
DB05476_15	2.8±0.3	2.7
DB06472_01	5.7±1.1	5.8
DB06472_02	11.5±1.7	12.2
DB06472_03	3.2±1.4	2.9
DB06472_04	4.0±2.7	2.7
DB06472_05	9.8±1.8	10.2
DB06472_06	4.4±1.5	3.9
DB06472_07	4.4±1.0	4.4
DB06472_08	3.8±1.0	3.8
DB06635_01	10.3±2.6	11.2
DB06635_02	3.1±0.4	3.1
DB06635_03	2.5±1.6	2.1
DB06635_04	2.9±0.7	2.7
DB06635_05	2.6±0.5	2.7
DB06635_06	3.6±0.5	3.5
DB06635_07	5.1±1.0	5.0
DB06635_09	5.3±1.6	4.9
DB06635_11	7.4±0.9	7.5
DB06635_12	6.5±2.2	7.8
DB12120_01	4.2±1.2	4.0
DB12120_02	5.8±1.1	6.2
DB12120_03	3.8±0.7	4.0
DB12120_04	2.9±0.6	2.8
DB12120_05	2.9±0.7	2.7
DB12120_06	3.7±0.6	3.7
DB12120_07	5.6±1.4	5.3
DB12598_01	3.3±0.7	3.2
DB12598_02	3.9±1.1	4.0
DB12598_03	5.8±2.9	6.5
DB12598_04	2.1±0.5	2.1
DB12598_05	2.8±0.7	2.6
DB12598_06	3.3±0.7	3.4
DB13000_01	5.6±1.6	5.5
DB13000_04	4.3±0.6	4.3
DB13000_05	6.4±1.5	7.1
DB13000_08	7.8±1.2	7.9
DB13000_09	7.8±0.6	7.9
DB13296_01	2.3±0.7	2.1
DB13296_03	7.0±1.8	7.6
DB13296_04	5.0±2.4	4.1
DB13296_06	2.3±0.7	2.2
DB13296_07	6.8±2.4	8.1
DB13296_09	2.5±0.6	2.4
DB13296_10	5.0±2.9	3.7
DB13296_12	5.0±2.5	4.1
DB13296_13	3.5±2.4	2.3
DB13296_14	6.6±1.5	7.2
DB13729_01	4.9±1.3	5.3

DB13729_02	4.2±1.0	3.8
DB13729_03	4.3±1.0	4.3
DB13729_04	3.1±0.6	2.9
DB13729_07	12.1±1.6	12.5
DB13729_08	9.2±1.5	9.4
DB13729_09	15.3±3.6	16.8
DB13729_10	7.2±1.2	7.6
DB13729_11	7.8±3.1	8.9
DB13729_12	5.0±0.6	5.1
DB14726_01	7.0±2.3	7.4
DB14726_03	10.0±2.4	10.5
DB14726_05	4.9±0.7	4.9
DB14726_07	12.5±3.5	13.9
DB14726_08	3.6±1.1	3.6
DB14726_09	8.2±1.0	8.4
DB14726_11	10.9±1.4	11.2
DB14726_12	5.9±2.7	5.0
DB14726_13	5.0±2.0	3.8
DB14726_15	4.0±0.7	3.9
DB14753_01	3.8±0.7	3.7
DB14753_02	2.9±1.0	3.0
DB14753_03	5.2±1.7	5.3
DB14753_04	2.6±0.7	2.4

Table S3. Mean and median d_{ON}^{min} distance calculated from 100 ns molecular dynamics simulations for each compound with different poses.

Compound ID_pose	d_{ON}^{min} (Å)	
	Mean±std	Median
CID2442_01	8.3±3.2	5.7
CID2442_02	5.3±1.3	5.2
CID2442_03	9.8±7.3	3.6
CID2442_04	5.7±0.6	5.8
CID2442_05	13.6±2.6	13.5
CID2882138_01	5.9±0.4	5.9
CID2882138_02	5.4±1.0	5.7
CID5395514_01	2.6±0.1	2.6
CID5395514_02	11.5±7.2	7.7
CID693919_01	4.1±0.9	3.7
CID693919_02	3.8±0.4	3.7
CID693919_03	9.6±1.1	8.9
CID765269_01	6.6±1.2	6.7
CID765269_02	15.7±9.3	28.9
CID765269_03	9.0±5.1	5.7
CID765269_04	7.1±1.0	7.3
CID765269_05	4.9±1.2	4.6
CID765269_06	12.5±8.0	17.8
CID765269_07	4.5±0.8	4.3
CID765269_08	4.2±1.0	3.8
CID765269_09	5.7±0.8	5.9
CID765269_10	4.3±1.1	3.8
DB00738_01	4.5±2.0	3.1
DB00738_03	8.4±5.0	2.8
DB00738_04	2.8±0.1	2.8
DB00738_05	2.8±0.1	2.8
DB00738_07	2.8±0.1	2.8
DB00738_08	2.8±0.1	2.8
DB00738_09	2.8±0.1	2.8
DB00738_10	2.8±0.1	2.8
DB00738_11	2.8±0.1	2.8
DB00738_12	2.8±0.1	2.8
DB03808_01	2.9±0.5	2.8
DB03808_02	2.8±0.1	2.8
DB03808_03	2.8±0.1	2.8
DB03808_04	2.8±0.1	2.8
DB03808_05	2.8±0.1	2.8
DB03808_06	2.8±0.1	2.8
DB03808_07	2.8±0.1	2.8
DB03808_08	2.8±0.1	2.8
DB03808_09	2.8±0.1	2.8
DB03808_10	2.8±0.1	2.8
DB05038_04	8.3±0.4	8.3
DB05038_05	15.1±1.6	15.0
DB05038_08	9.8±1.5	11.9
DB05038_09	7.6±1.1	7.4
DB05038_10	2.8±0.2	2.7
DB05038_13	11.3±1.0	11.5
DB05038_14	7.5±0.5	7.4
DB05038_15	13.2±1.4	13.0
DB05038_16	14.9±1.7	15.1

DB05038_18	8.4±0.4	8.4
DB05476_01	2.9±0.2	2.9
DB05476_02	2.9±0.2	2.9
DB05476_05	2.8±0.1	2.8
DB05476_06	2.8±0.1	2.8
DB05476_07	2.8±0.1	2.8
DB05476_08	2.8±0.1	2.8
DB05476_10	8.6±0.4	8.6
DB05476_14	3.4±1.1	2.9
DB05476_15	3.2±0.7	3.0
DB06472_01	2.7±0.1	2.7
DB06472_02	7.2±2.8	8.3
DB06472_03	2.8±0.1	2.8
DB06472_04	2.8±0.1	2.8
DB06472_05	5.3±1.1	5.5
DB06472_06	2.8±0.1	2.8
DB06472_07	2.8±0.1	2.8
DB06472_08	2.8±0.1	2.8
DB06635_01	2.8±0.1	2.8
DB06635_02	2.8±0.1	2.8
DB06635_03	3.4±1.0	2.9
DB06635_04	2.8±0.1	2.8
DB06635_05	2.8±0.1	2.8
DB06635_06	2.8±0.1	2.8
DB06635_07	2.8±0.1	2.8
DB06635_09	2.8±0.1	2.8
DB06635_11	2.8±0.1	2.8
DB06635_12	5.4±0.6	5.5
DB12120_01	2.7±0.1	2.7
DB12120_02	4.4±0.9	4.7
DB12120_03	2.8±0.1	2.8
DB12120_04	2.7±0.1	2.7
DB12120_05	2.8±0.1	2.7
DB12120_06	2.8±0.2	2.7
DB12120_07	10.4±1.3	11.6
DB12598_01	2.8±0.1	2.8
DB12598_02	2.8±0.1	2.8
DB12598_03	2.8±0.1	2.8
DB12598_04	2.8±0.1	2.8
DB12598_05	3.2±0.8	2.8
DB12598_06	4.2±1.1	4.7
DB13000_01	3.3±0.5	3.1
DB13000_04	2.8±0.1	2.8
DB13000_05	2.8±0.1	2.8
DB13000_08	8.8±2.1	5.4
DB13000_09	2.8±0.1	2.8
DB13296_01	2.8±0.1	2.8
DB13296_03	2.8±0.1	2.8
DB13296_04	2.8±0.1	2.8
DB13296_06	2.8±0.1	2.8
DB13296_07	2.8±0.1	2.8
DB13296_09	2.8±0.1	2.8
DB13296_10	2.8±0.1	2.8
DB13296_12	2.8±0.1	2.8
DB13296_13	3.3±1.2	2.8
DB13296_14	2.8±0.1	2.8

DB13729_01	2.8±0.1	2.8
DB13729_02	3.8±1.0	3.2
DB13729_03	2.8±0.1	2.8
DB13729_04	2.8±0.1	2.8
DB13729_07	2.9±0.2	2.8
DB13729_08	3.0±0.4	2.9
DB13729_09	10.8±4.5	12.9
DB13729_10	2.8±0.1	2.8
DB13729_11	2.8±0.1	2.8
DB13729_12	9.2±1.3	9.0
DB14726_01	3.0±0.5	2.8
DB14726_03	3.0±0.5	2.9
DB14726_05	2.8±0.1	2.8
DB14726_07	9.6±1.8	7.8
DB14726_08	2.7±0.1	2.7
DB14726_09	2.7±0.1	2.7
DB14726_11	2.8±0.1	2.7
DB14726_12	2.8±0.1	2.8
DB14726_13	2.8±0.1	2.8
DB14726_15	2.7±0.1	2.7
DB14753_01	2.7±0.1	2.7
DB14753_02	2.7±0.1	2.7
DB14753_03	2.8±0.2	2.7
DB14753_04	2.7±0.1	2.7

Table S4. MM/GBSA binding free energy for the control compounds bound to TMPRSS2.

Compound ID_pose	ΔE_{vdW} (kcal/mol)	ΔE_{ele} (kcal/mol)	ΔG_{GB} (kcal/mol)	ΔG_{SA} (kcal/mol)	$-T\Delta S$ (kcal/mol)	ΔG_{bind} (kcal/mol)
CID2442_04	−36.46±2.79	−16.51±6.30	20.94±5.60	−3.65±0.18	15.09	−20.60±3.05
CID2882138_02	−34.28±2.12	−40.56±5.20	44.14±4.82	−3.29±0.16	13.00	−21.00±2.01
CID5395514_01	−30.48±2.96	−12.27±1.97	14.60±1.79	−3.19±0.19	5.73	−25.61±2.36
CID693919_01	−31.70±2.62	−5.85±1.50	10.78±1.29	−3.25±0.26	4.36	−25.67±2.71
CID765269_05	−23.91±3.09	−4.57±1.70	9.94±1.68	−2.95±0.38	9.12	−12.37±3.03

Supporting Figures

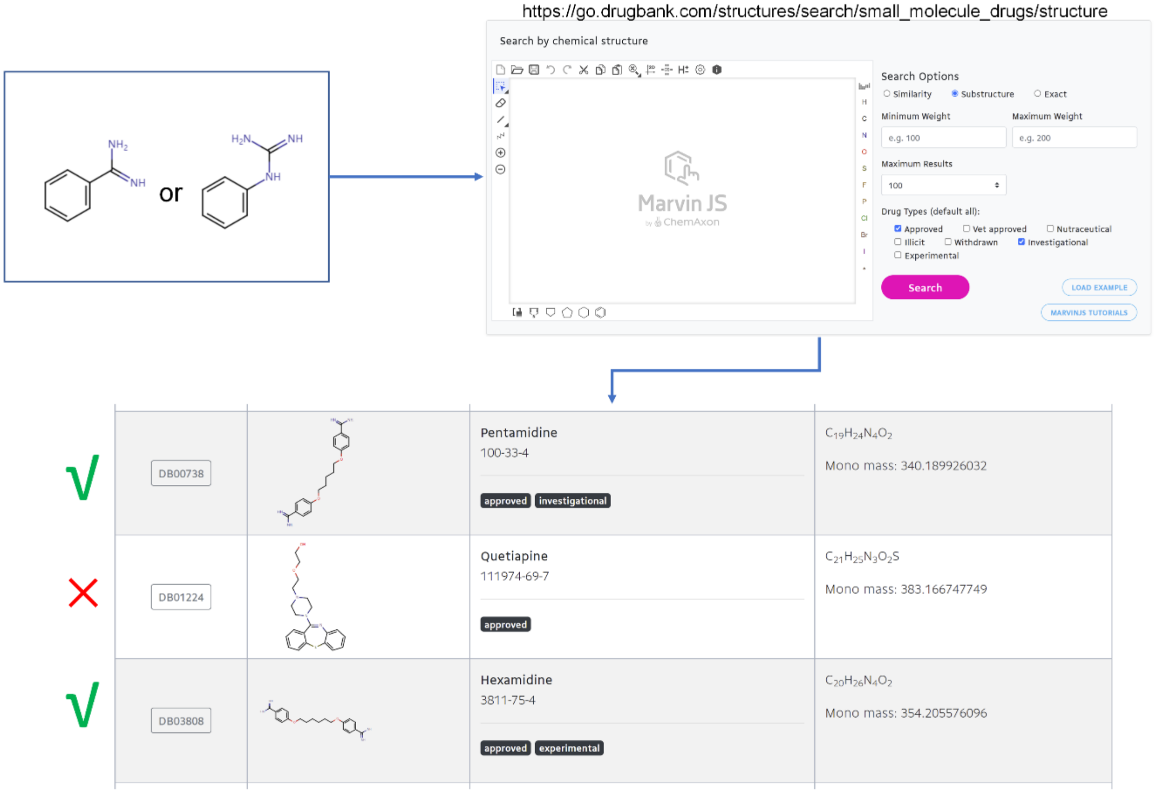

Figure S1. The workflow for searching and narrowing down the hit list of guanidinobenzoyl- or aminidinobenzoyl-containing drugs.

Summary statistics

All-Atom Contacts	Clashscore, all atoms:	1.65		99th percentile* (N=1784, all resolutions)
	Clashscore is the number of serious steric overlaps (> 0.4 Å) per 1000 atoms.			
Protein Geometry	Poor rotamers	0	0.00%	Goal: <0.3%
	Favored rotamers	198	100.00%	Goal: >98%
	Ramachandran outliers	1	0.43%	Goal: <0.05%
	Ramachandran favored	231	98.30%	Goal: >98%
	Rama distribution Z-score	0.50 ± 0.55		Goal: abs(Z score) < 2
	MolProbity score^	0.91		100th percentile* (N=27675, 0Å - 99Å)
	Cβ deviations >0.25Å	0	0.00%	Goal: 0
	Bad bonds:	0 / 1898	0.00%	Goal: 0%
	Bad angles:	5 / 2590	0.19%	Goal: <0.1%
Peptide Omegas	Cis Prolines:	0 / 14	0.00%	Expected: ≤1 per chain, or ≤5%
Low-resolution Criteria	CaBLAM outliers	5	2.1%	Goal: <1.0%
	CA Geometry outliers	4	1.72%	Goal: <0.5%
Additional validations	Chiral volume outliers	0/277		
	Waters with clashes	0/0	0.00%	See UnDowser table for details

In the two column results, the left column gives the raw count, right column gives the percentage.

* 100th percentile is the best among structures of comparable resolution; 0th percentile is the worst. For clashscore the comparative set of structures was selected in 2004, for MolProbity score in 2006.

^ MolProbity score combines the clashscore, rotamer, and Ramachandran evaluations into a single score, normalized to be on the same scale as X-ray resolution.

Key to table colors and cutoffs here:

Figure S2. Summary of the statistics for the MolProbity assessment of the TMPRSS2 model.

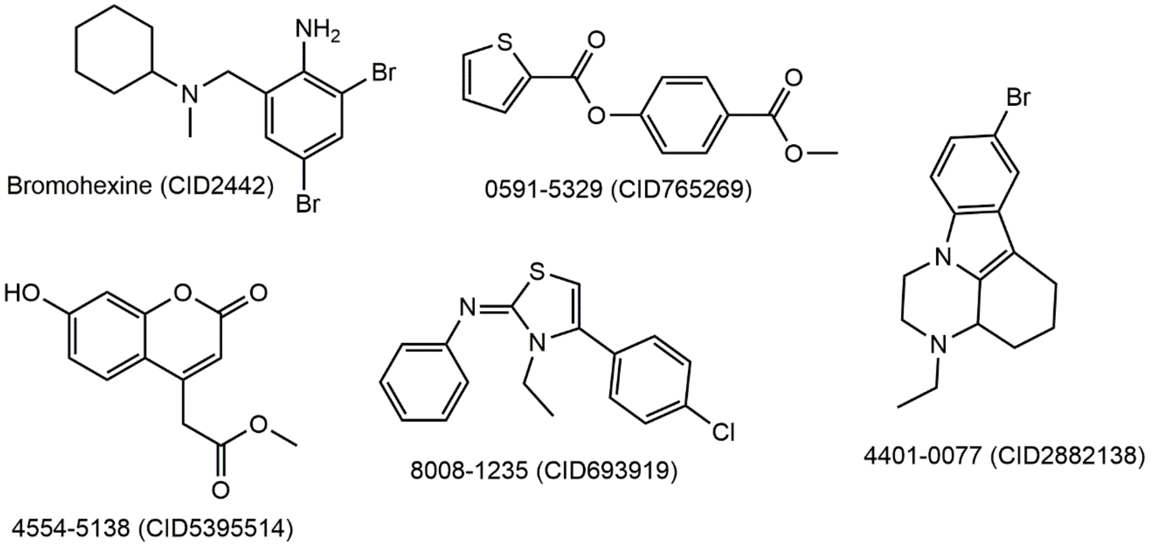

Figure S3. Five weak TMPRSS2 inhibitors used as control compounds in this study.

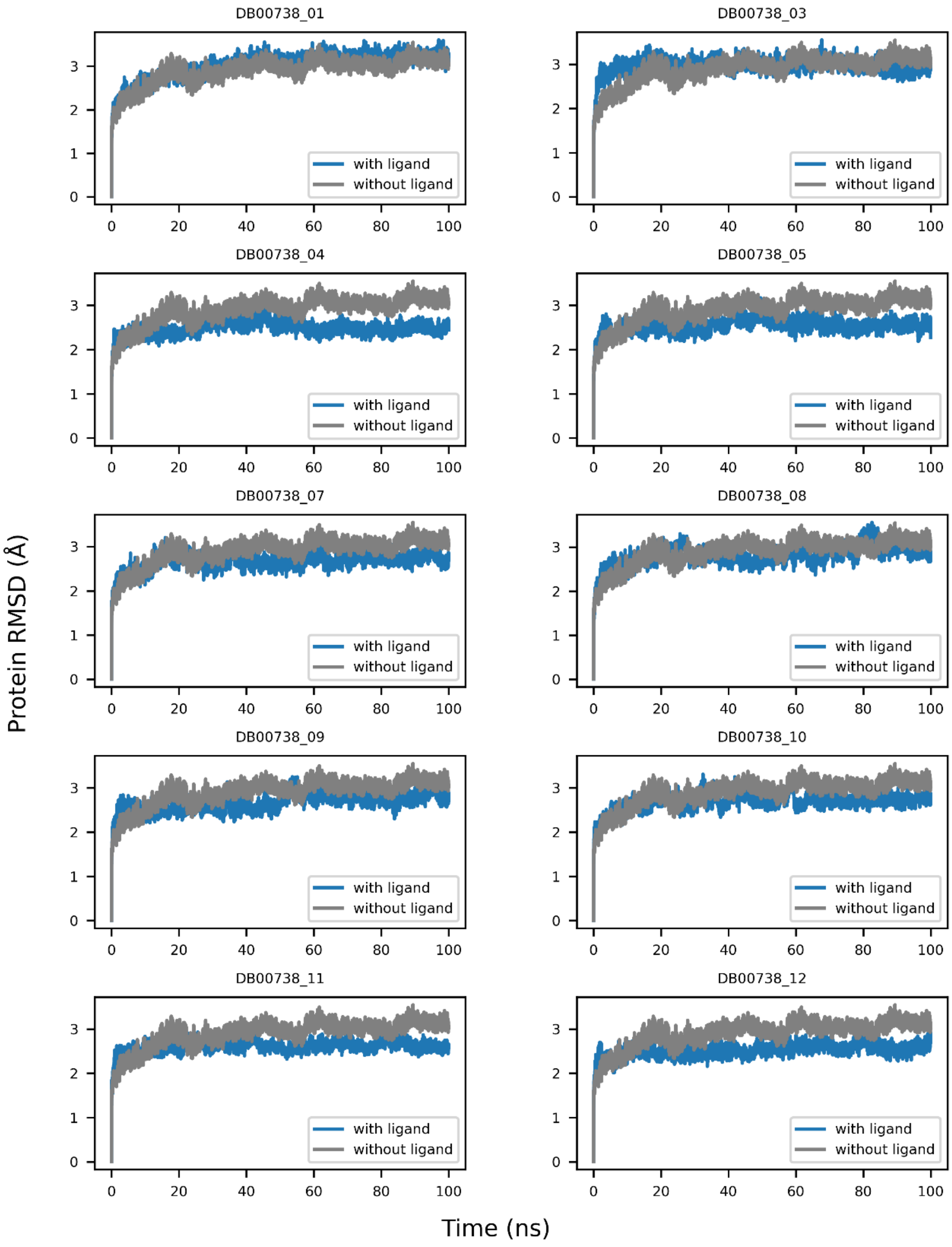

Figure S4. RMSD of TMPRSS2 calculated from 100 ns molecular dynamics simulations for each compound with different poses. The apo-form TMPRSS2 was simulated only once and its trajectory was used as a baseline for comparison.

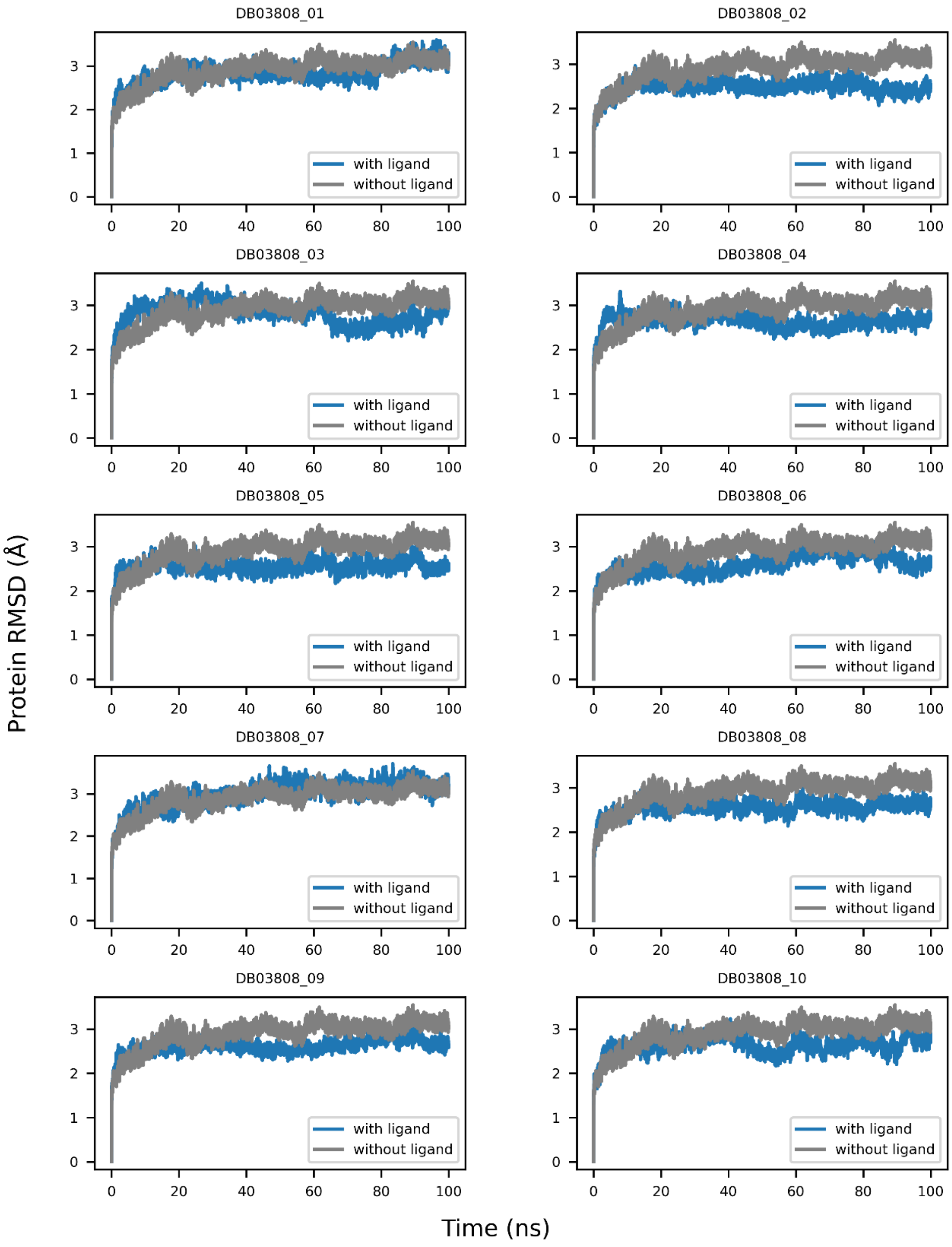

(Figure S4 continued)

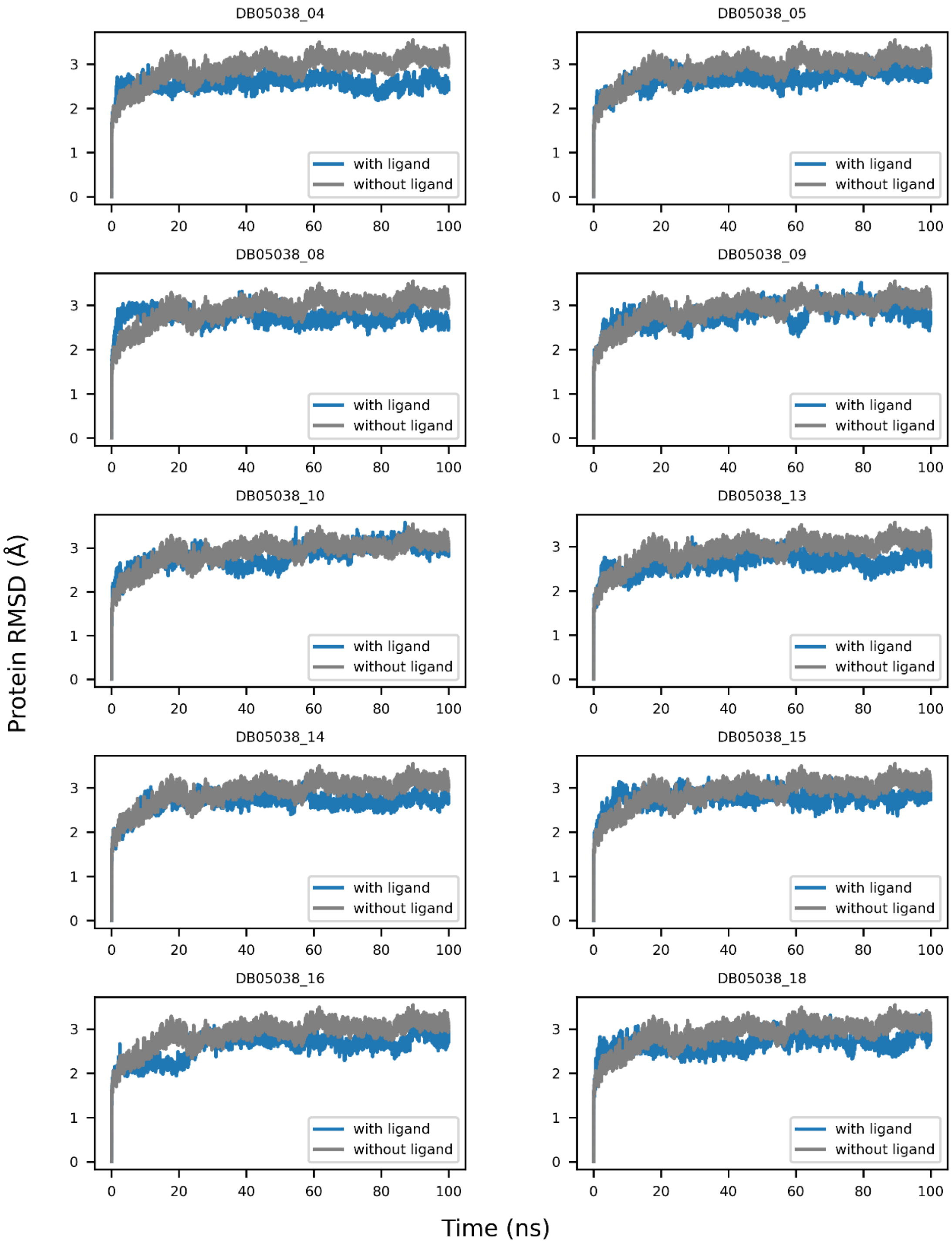

(Figure S4 continued)

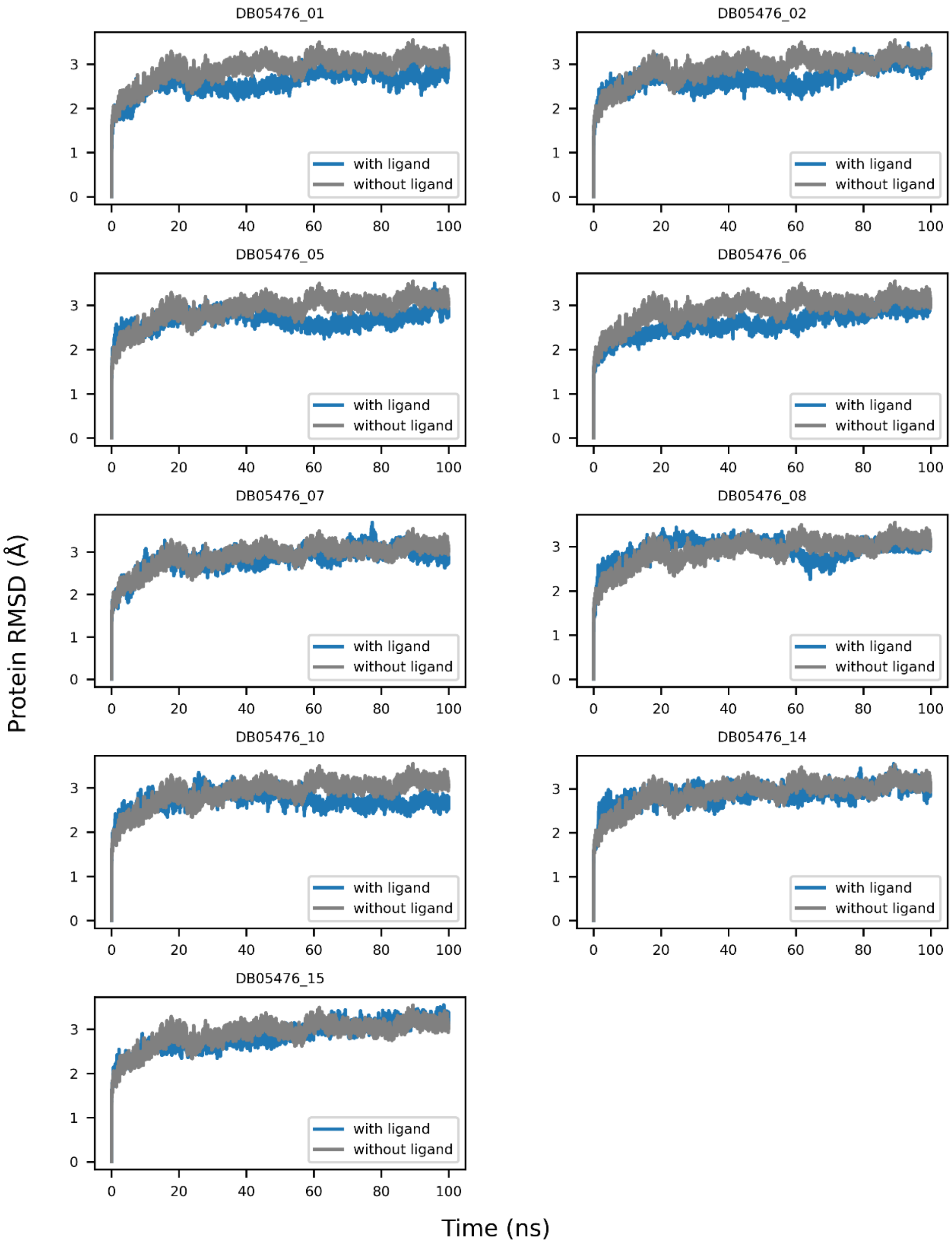

(Figure S4 continued)

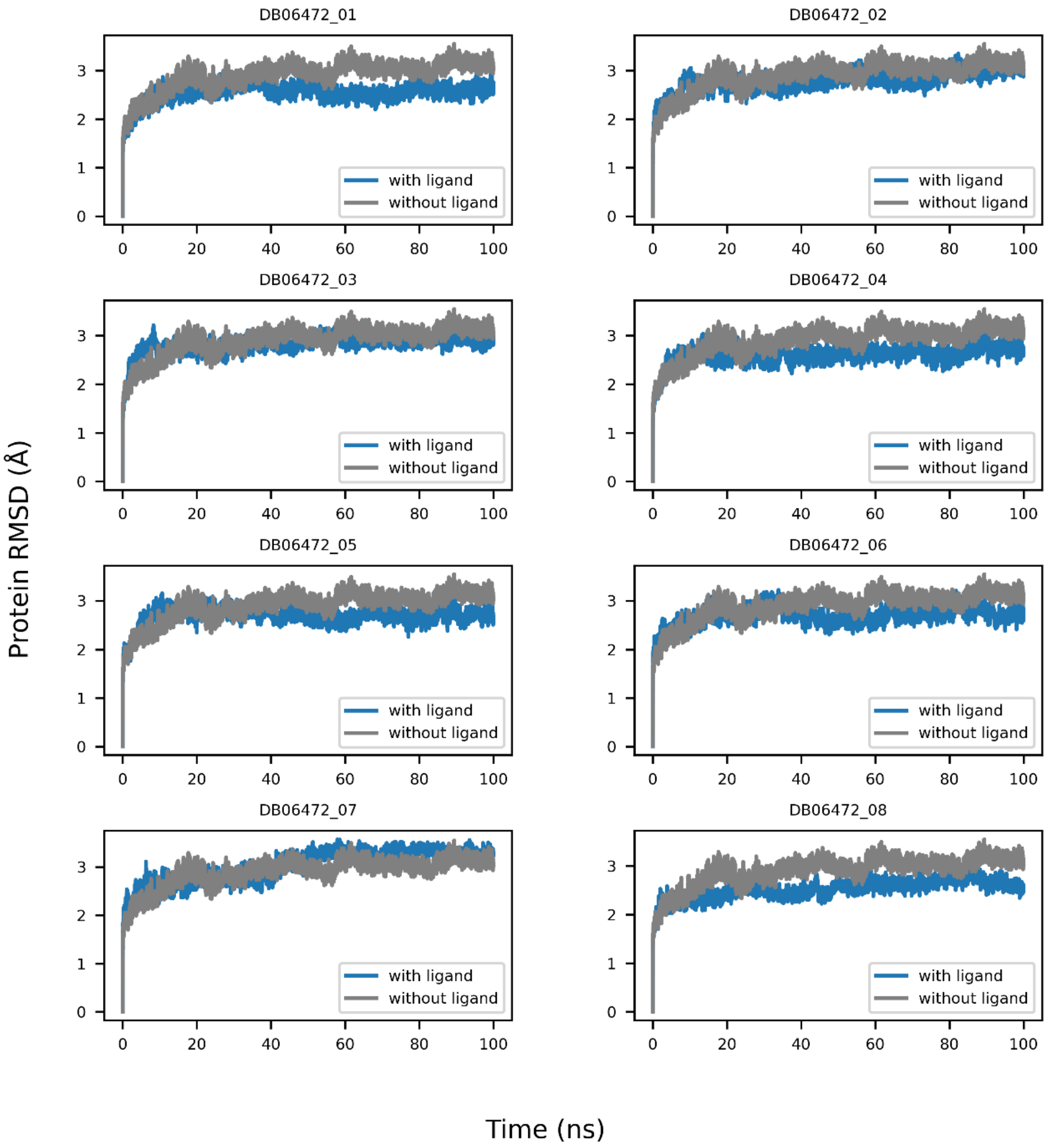

(Figure S4 continued)

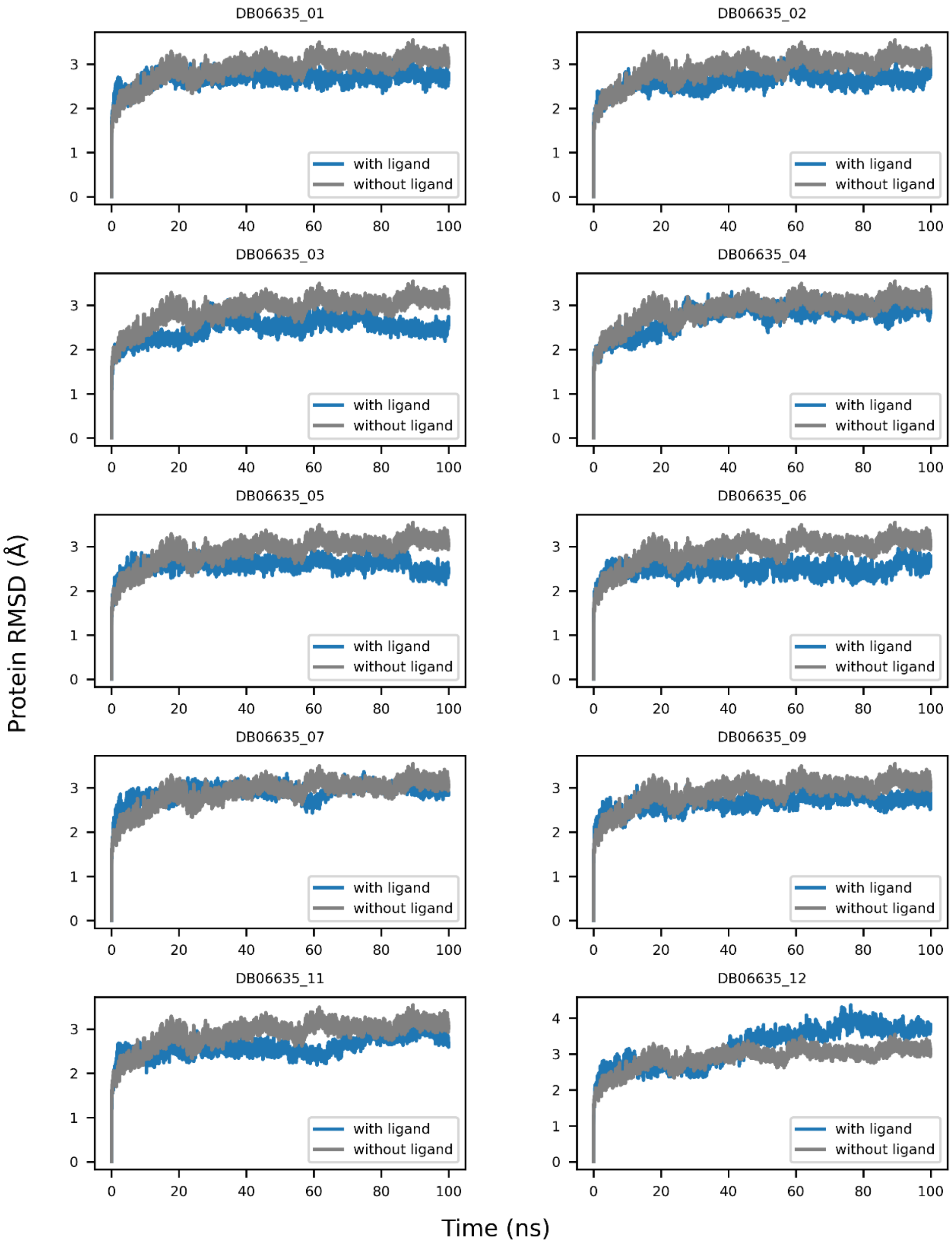

(Figure S4 continued)

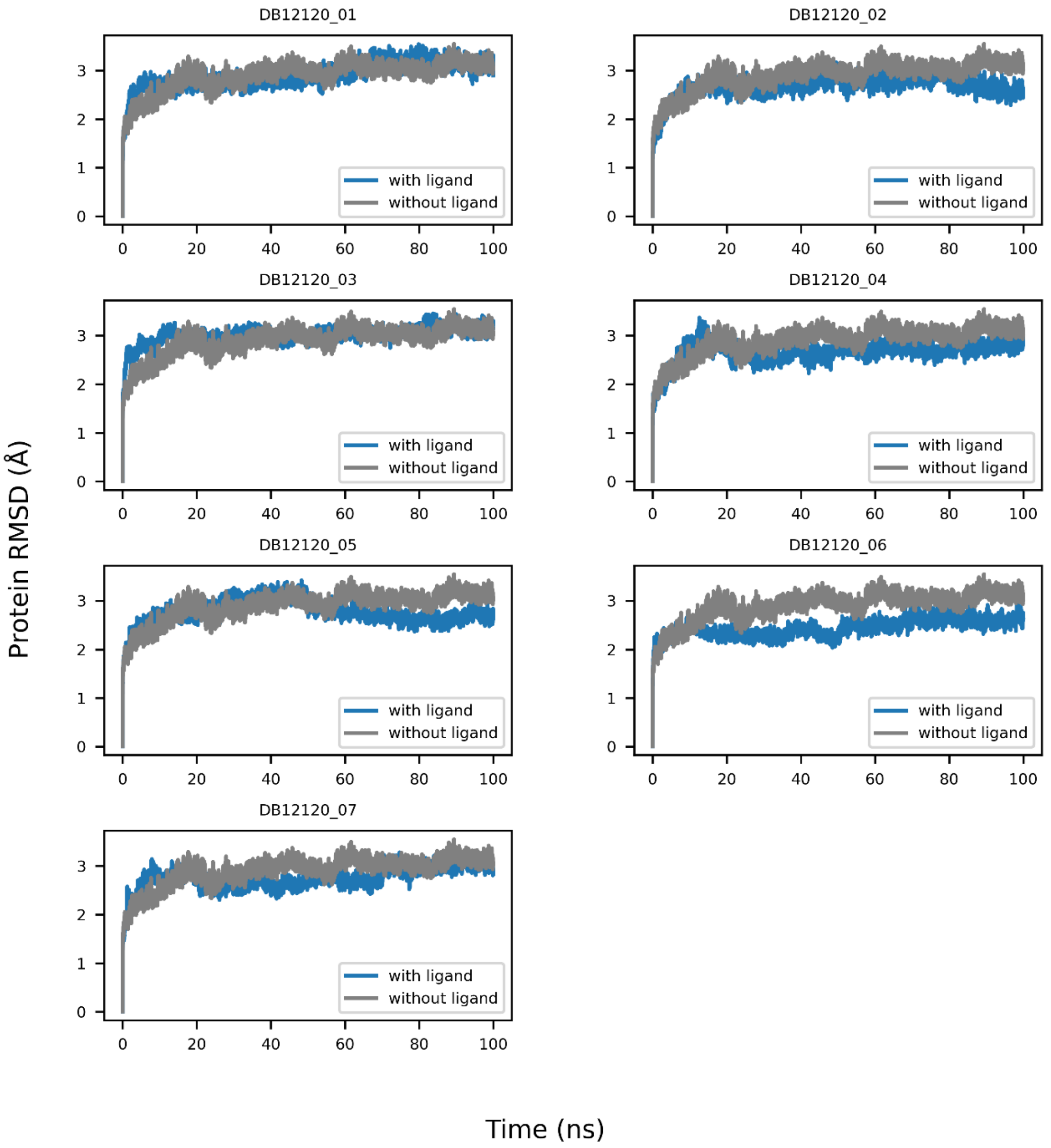

(Figure S4 continued)

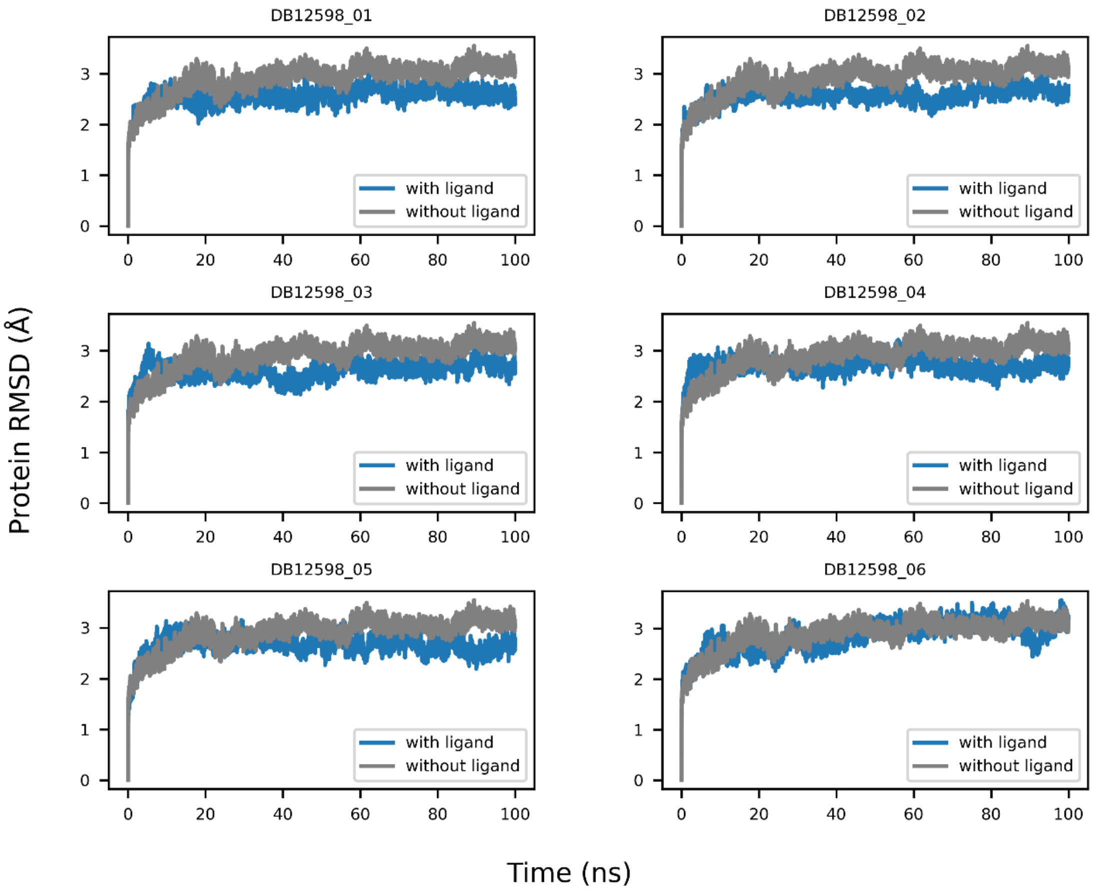

(Figure S4 continued)

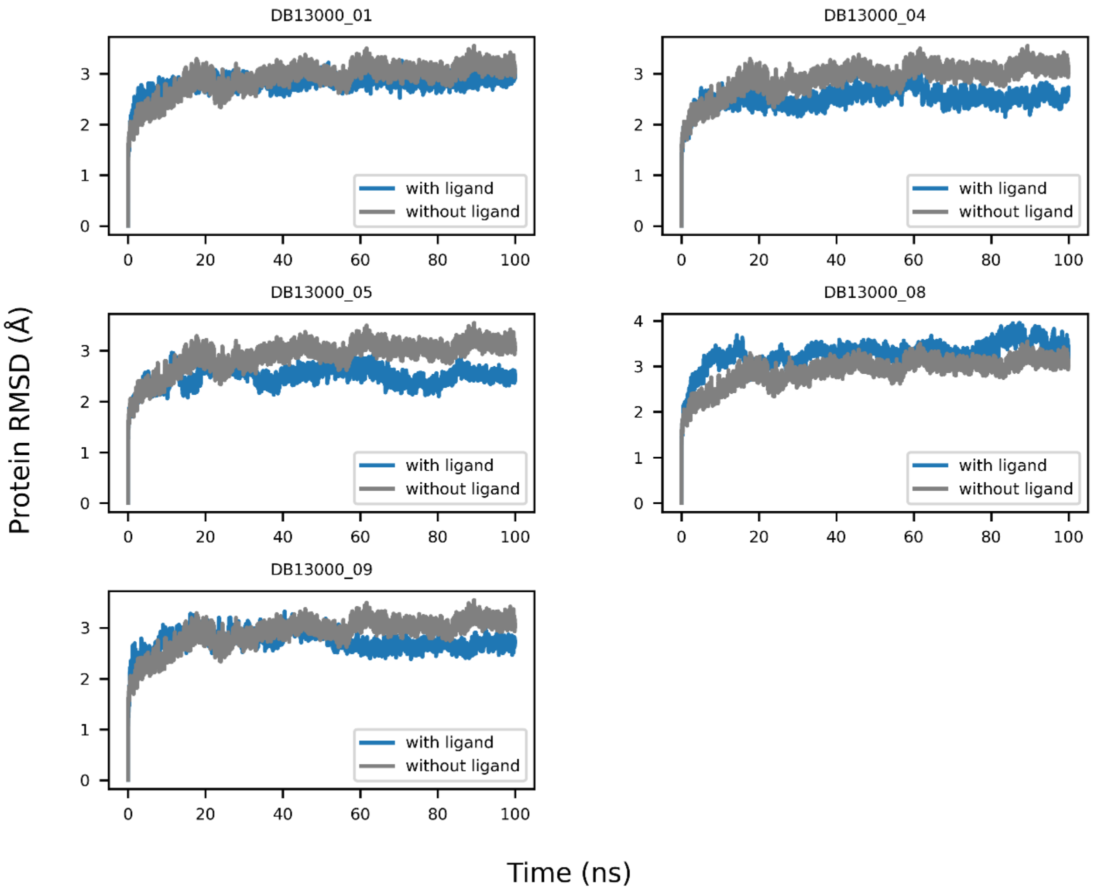

(Figure S4 continued)

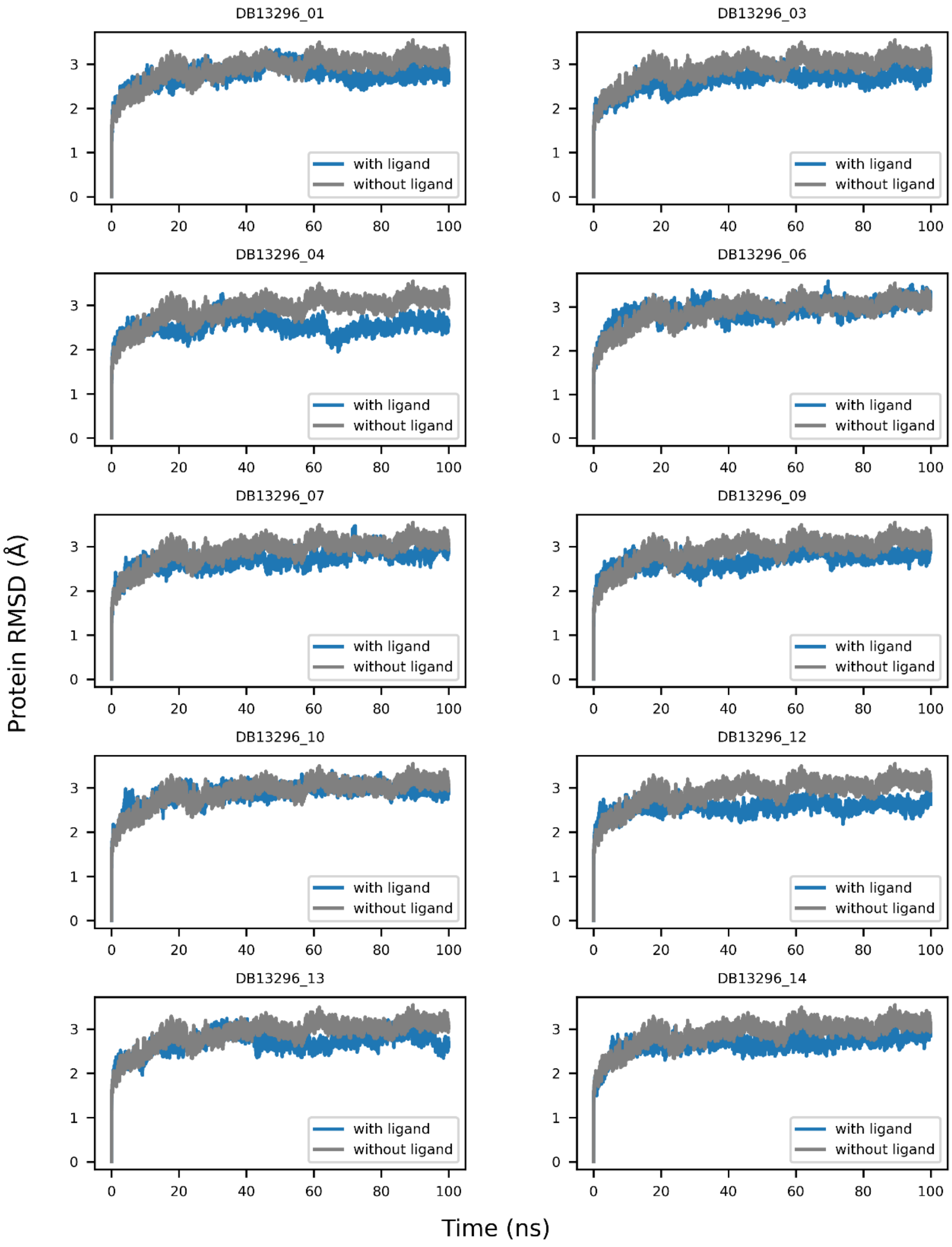

(Figure S4 continued)

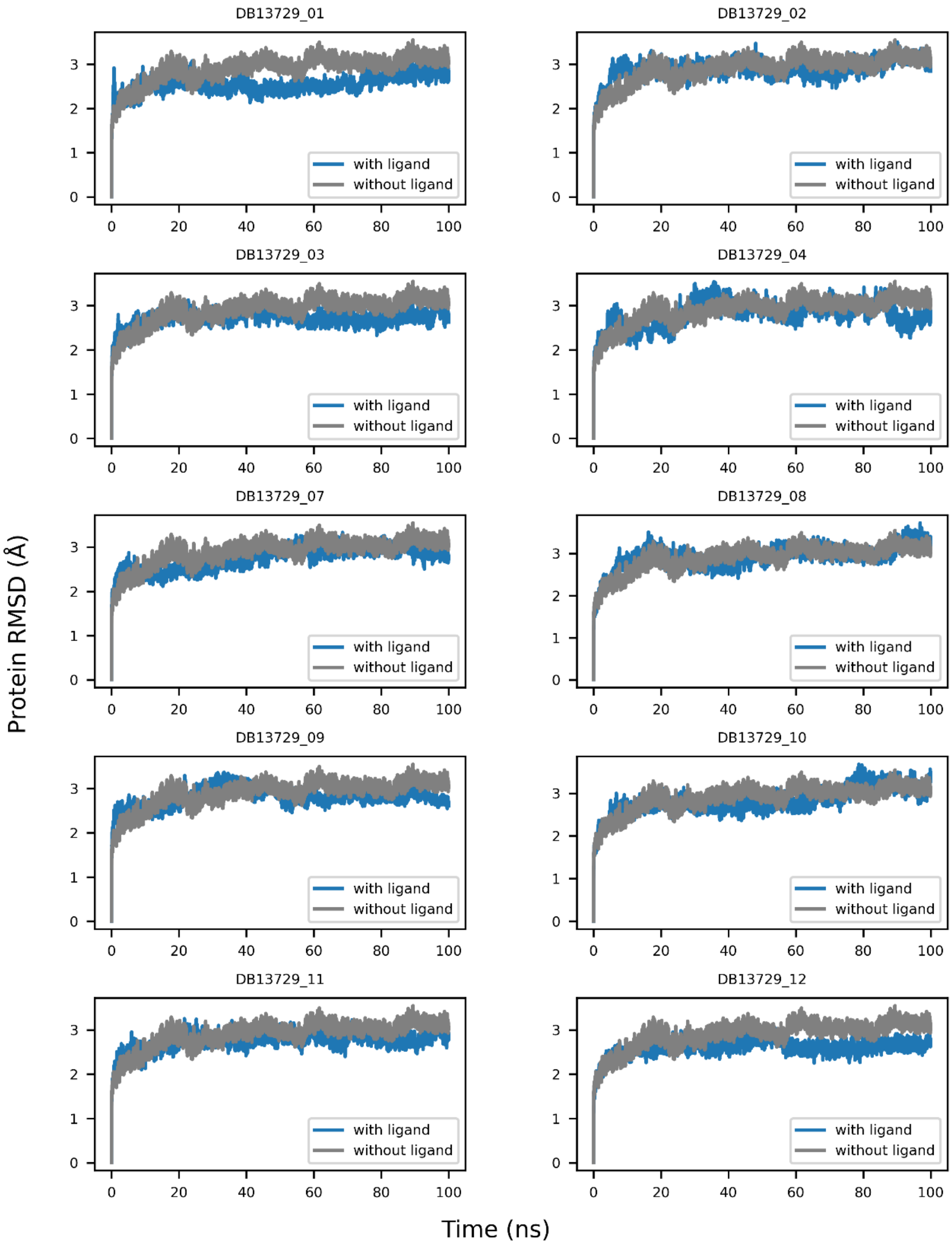

(Figure S4 continued)

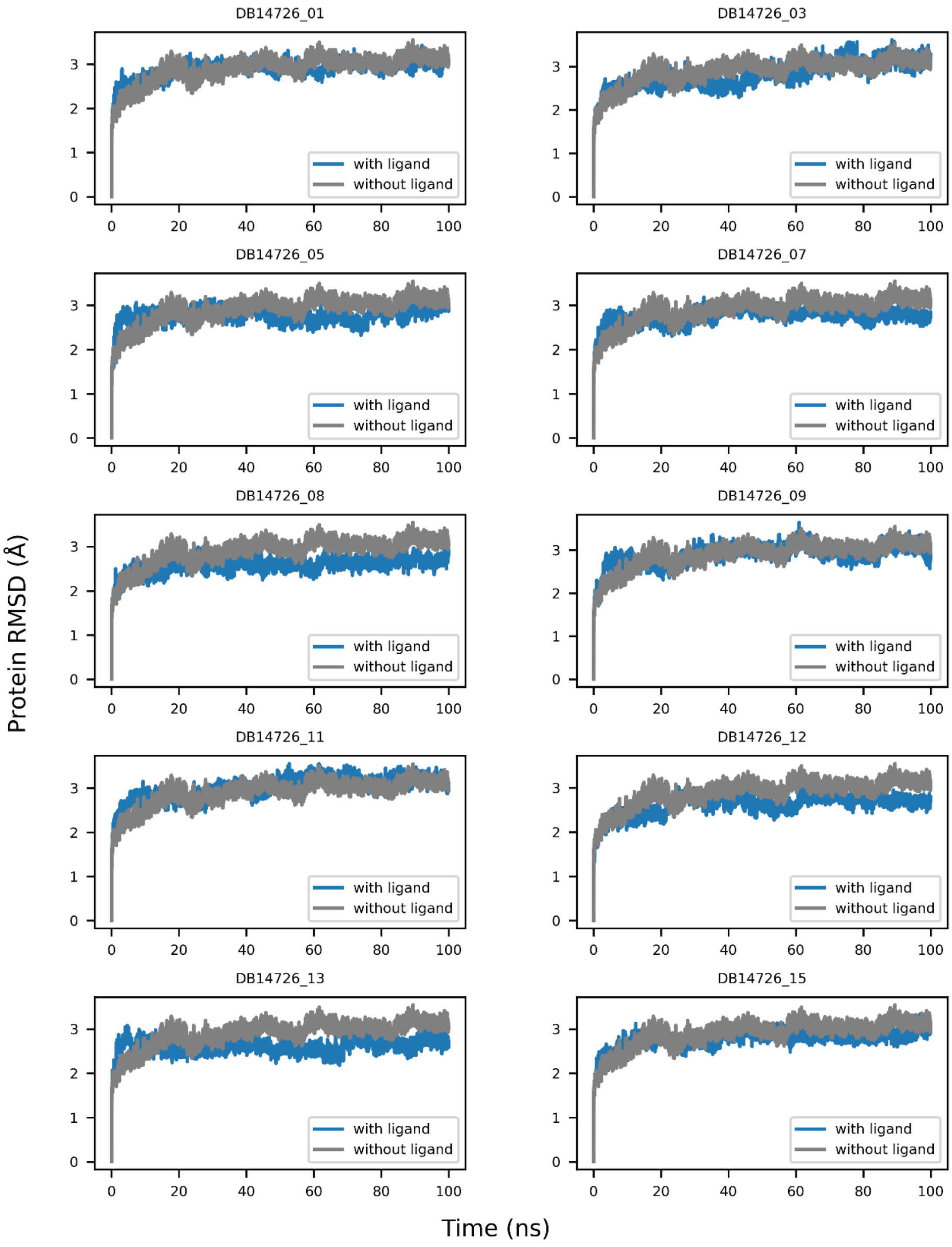

(Figure S4 continued)

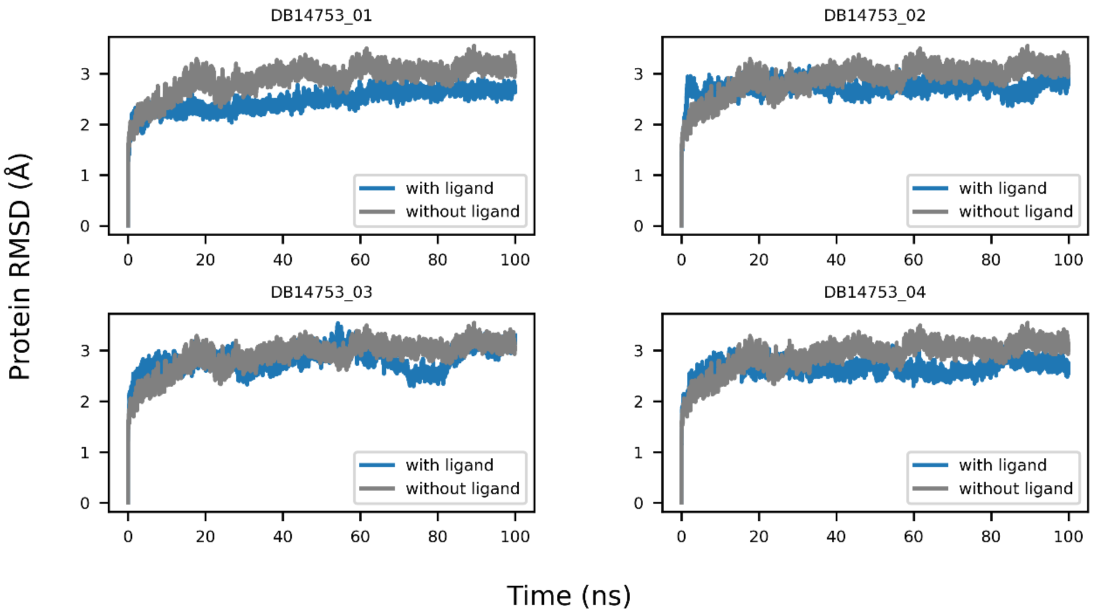

(Figure S4 continued)

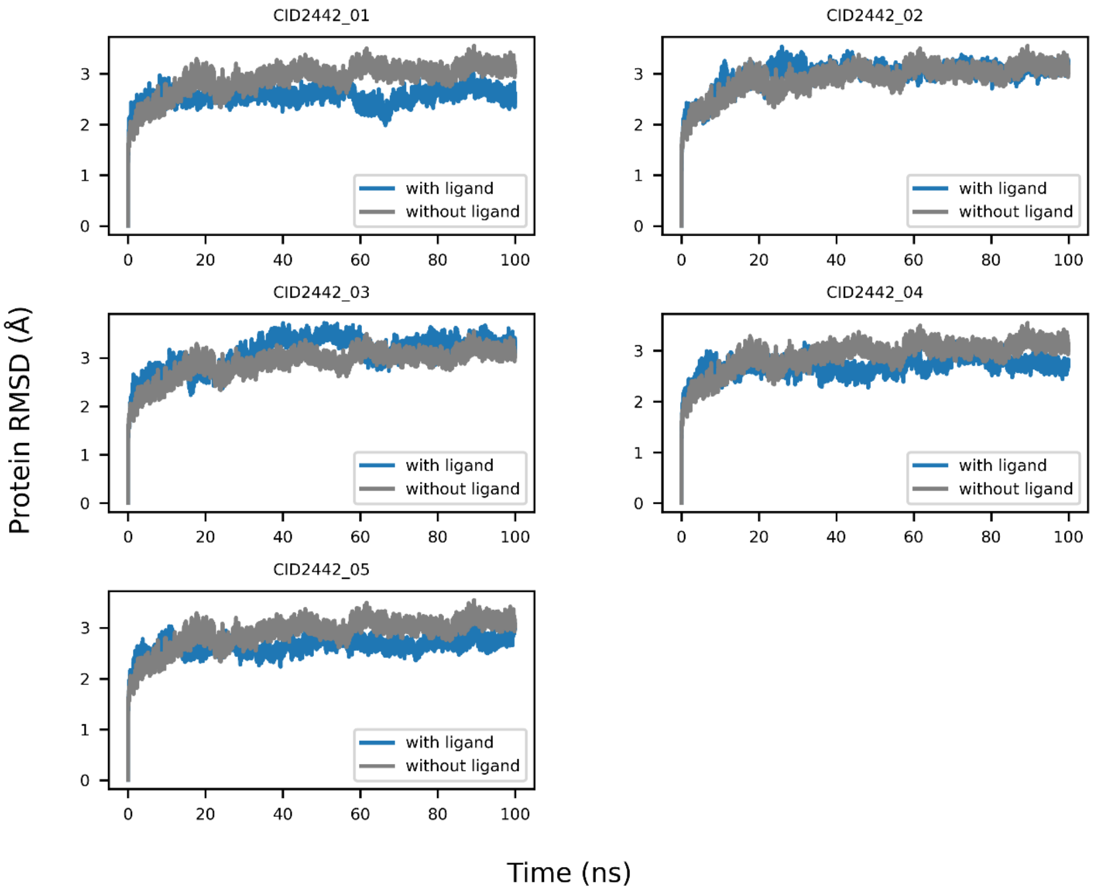

(Figure S4 continued)

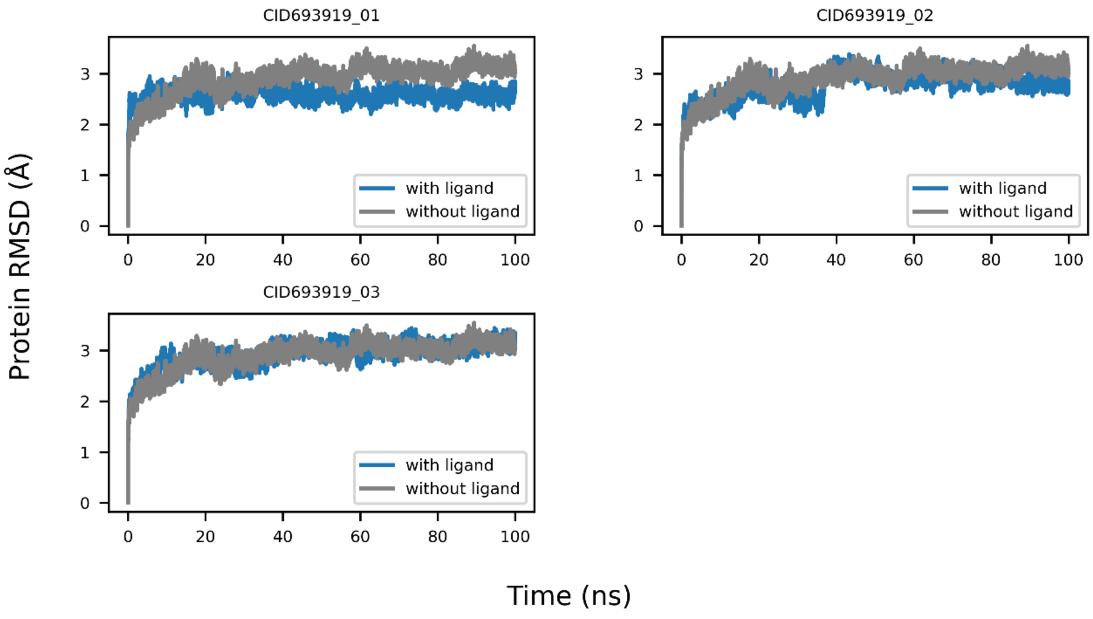

(Figure S4 continued)

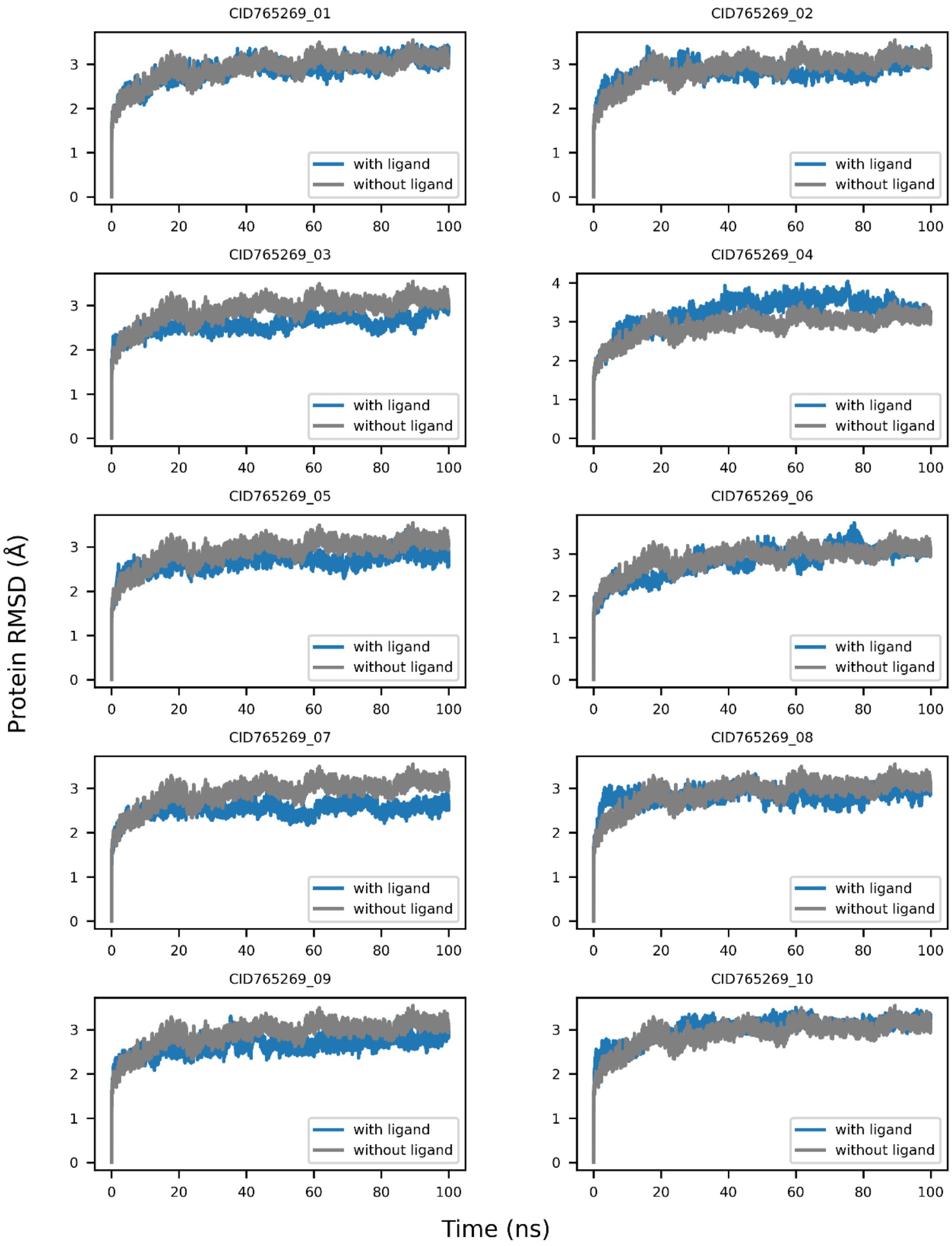

(Figure S4 continued)

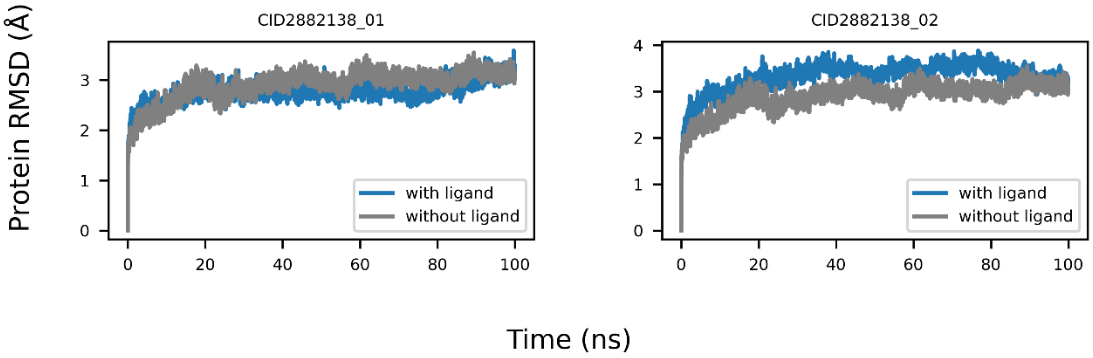

(Figure S4 continued)

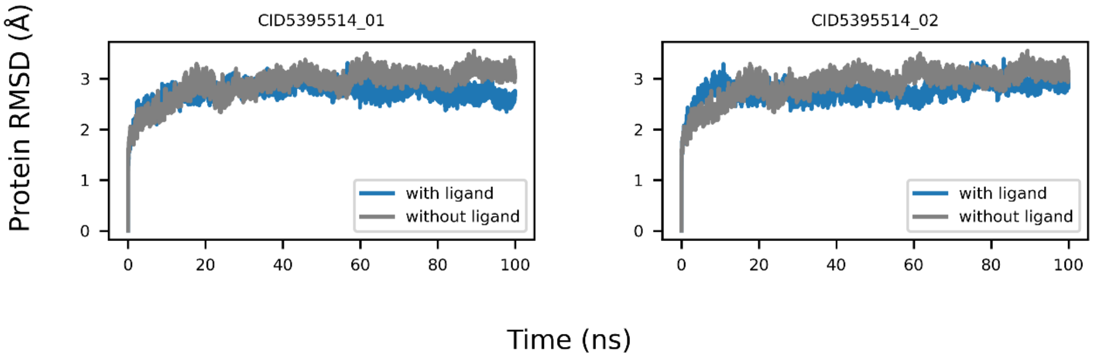

(Figure S4 continued)

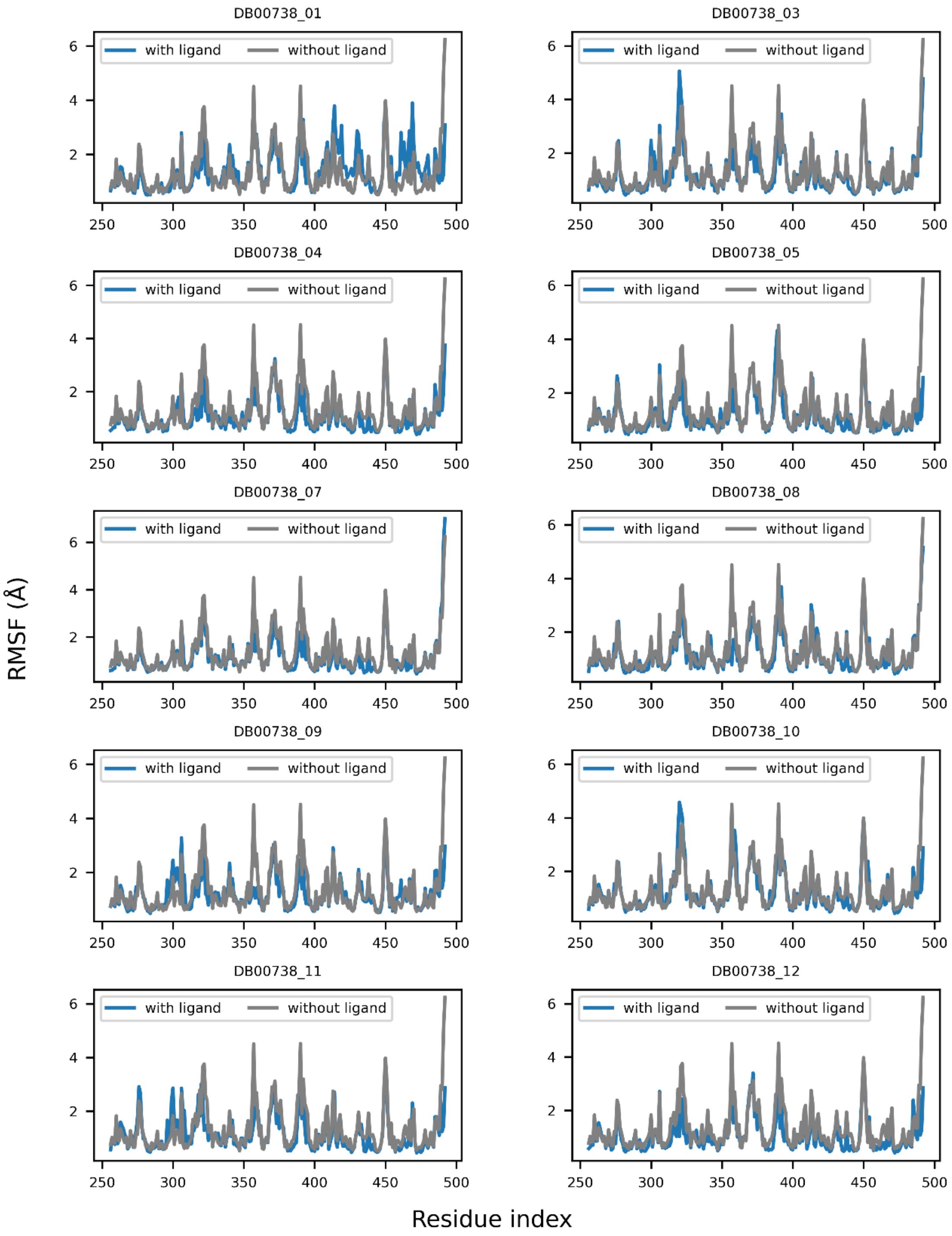

Figure S5. RMSF of TMPRSS2 residues calculated from 100 ns molecular dynamics simulations for each compound with different poses. The apo-form TMPRSS2 was simulated only once and its trajectory was used as a baseline for comparison. The residues with high flexibility (> 3 Å), Met320, Phe321, Phe357, Lys390, Asn450, and Gly492, are distant from the binding pocket.

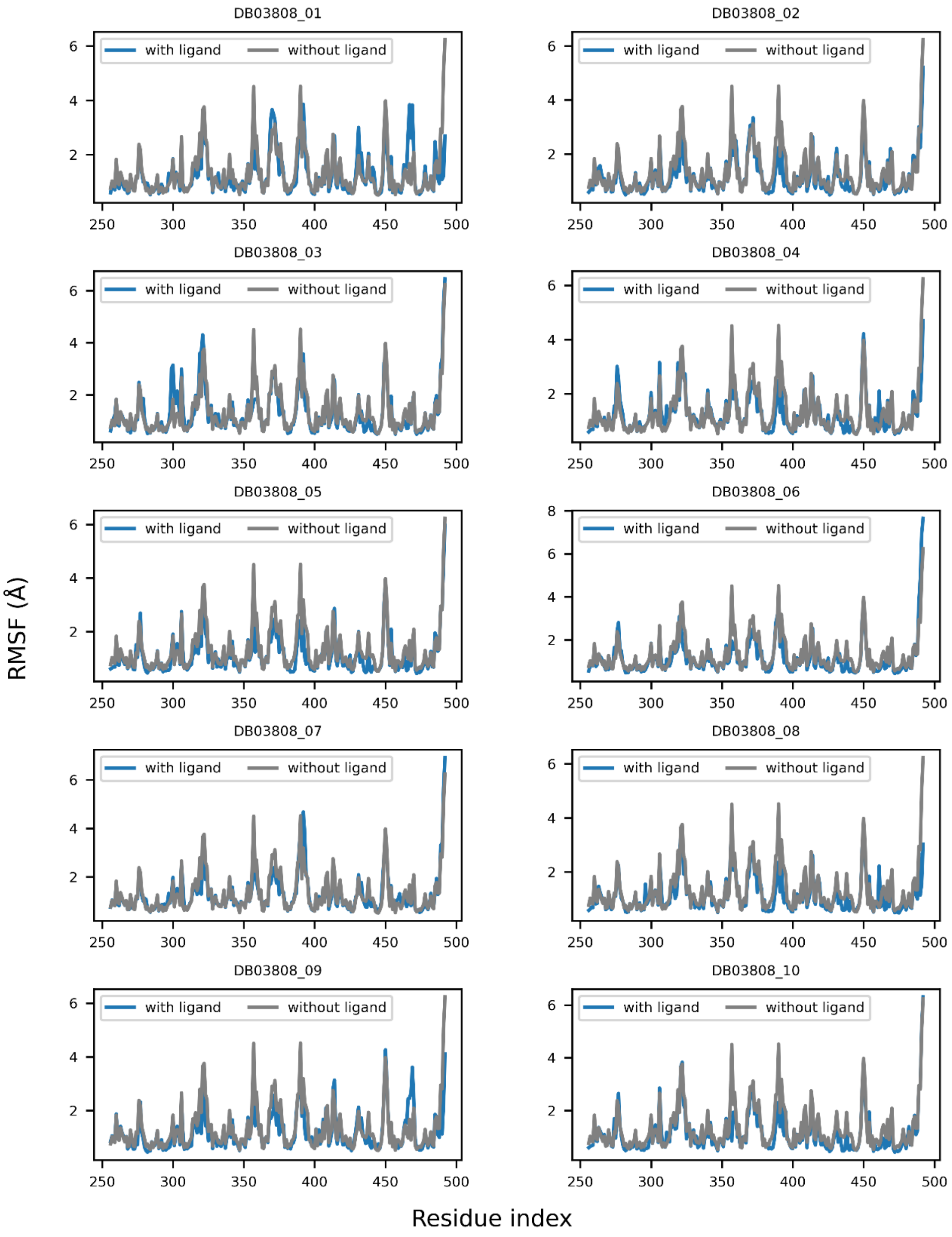

(Figure S5 continued)

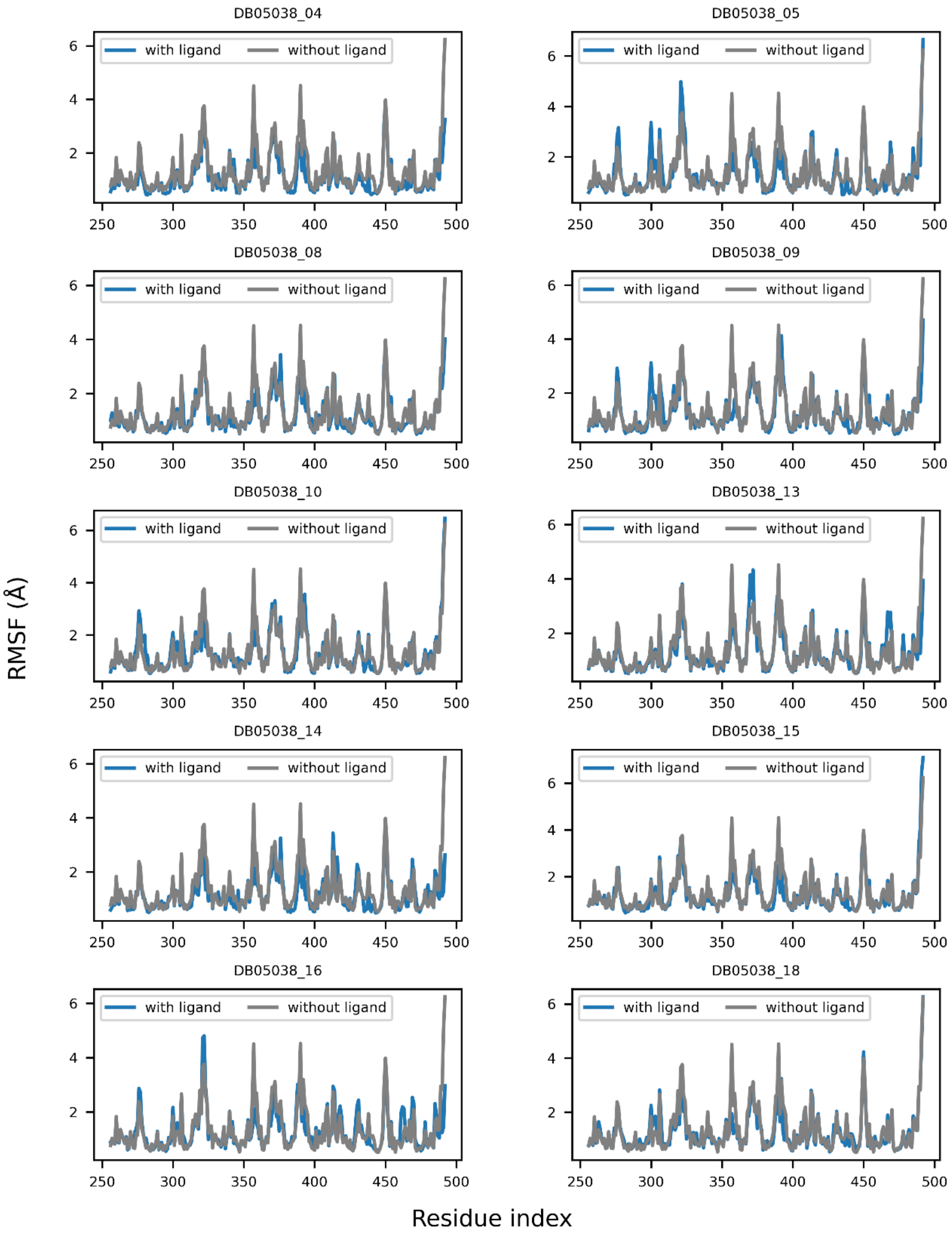

(Figure S5 continued)

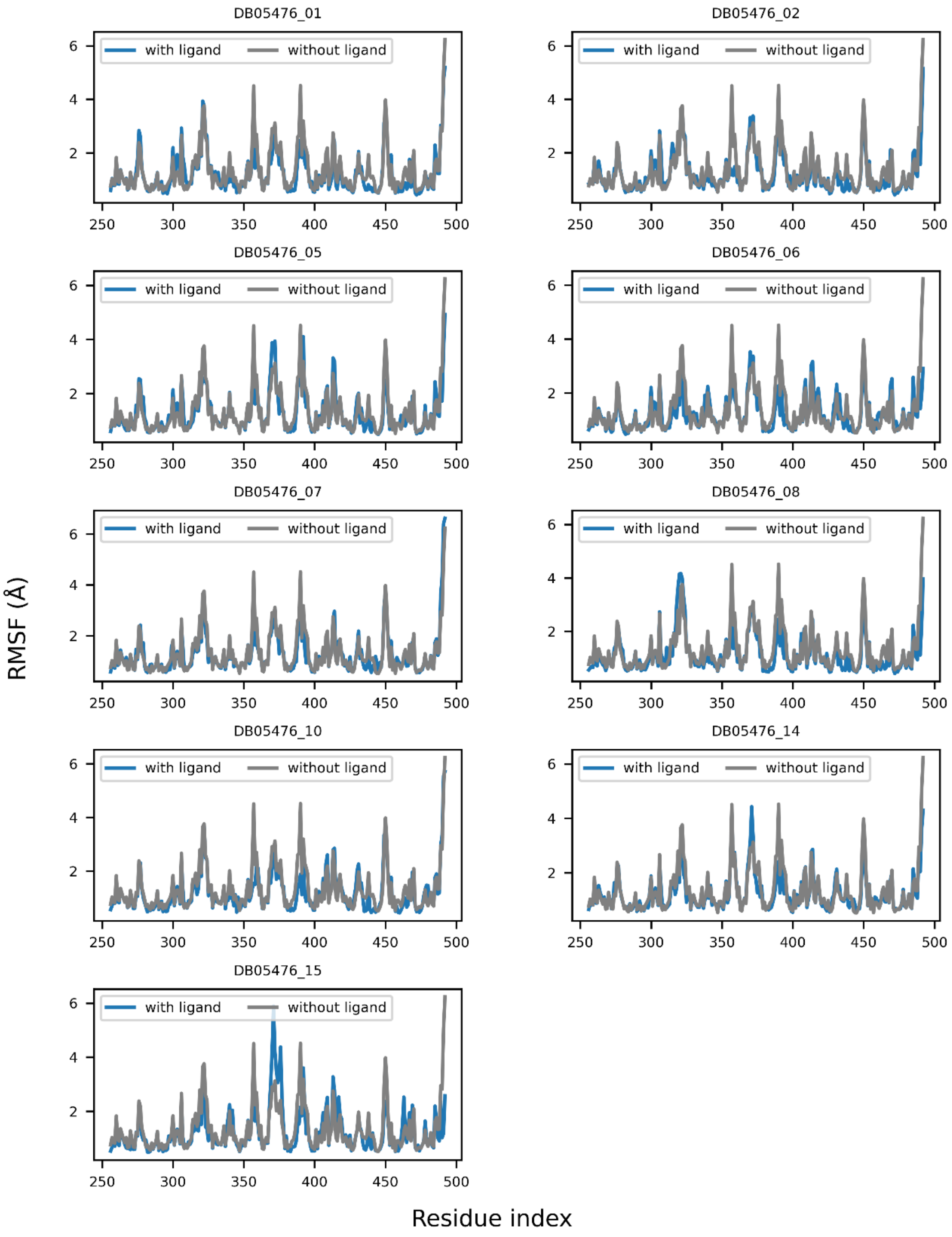

(Figure S5 continued)

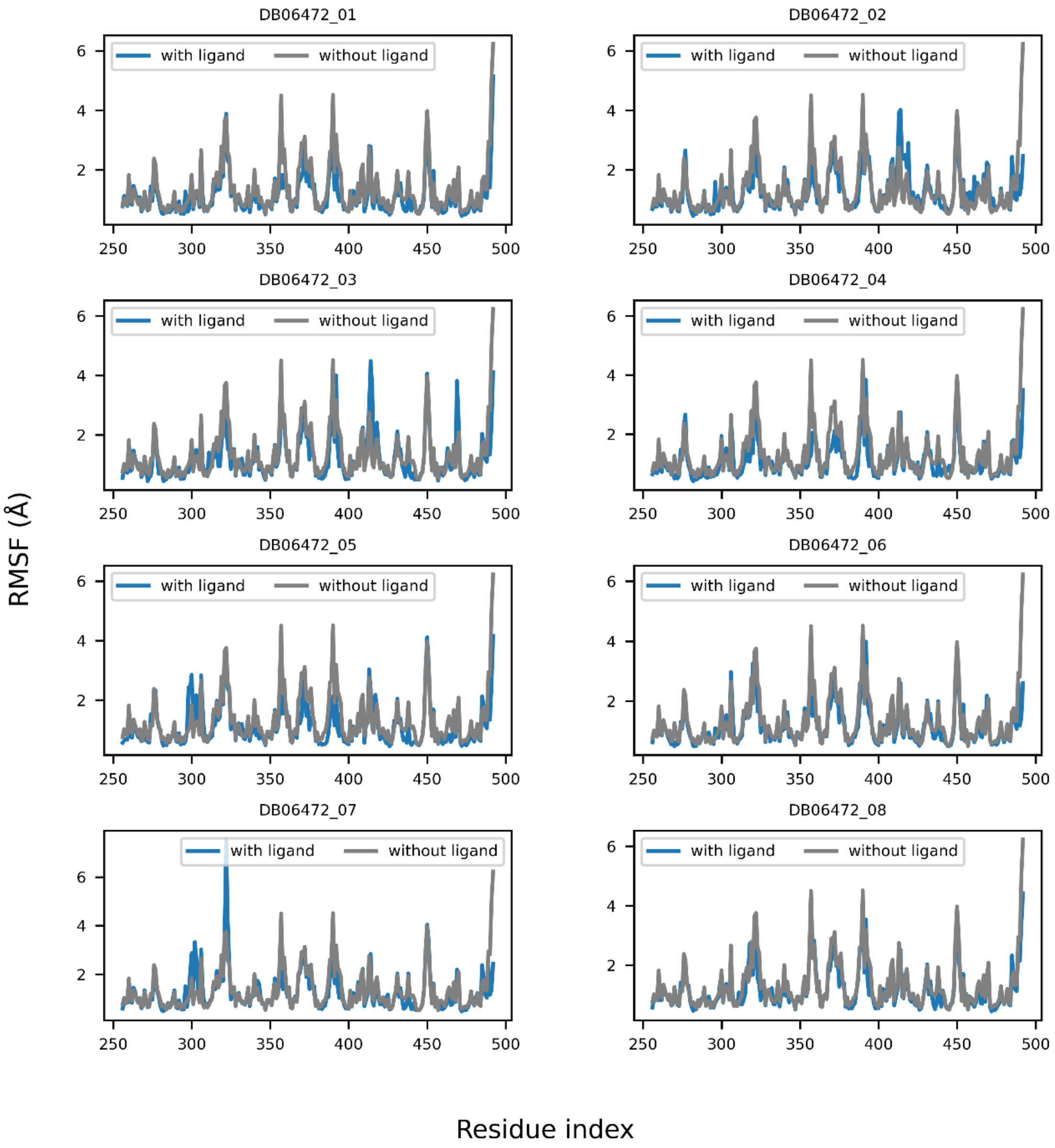

(Figure S5 continued)

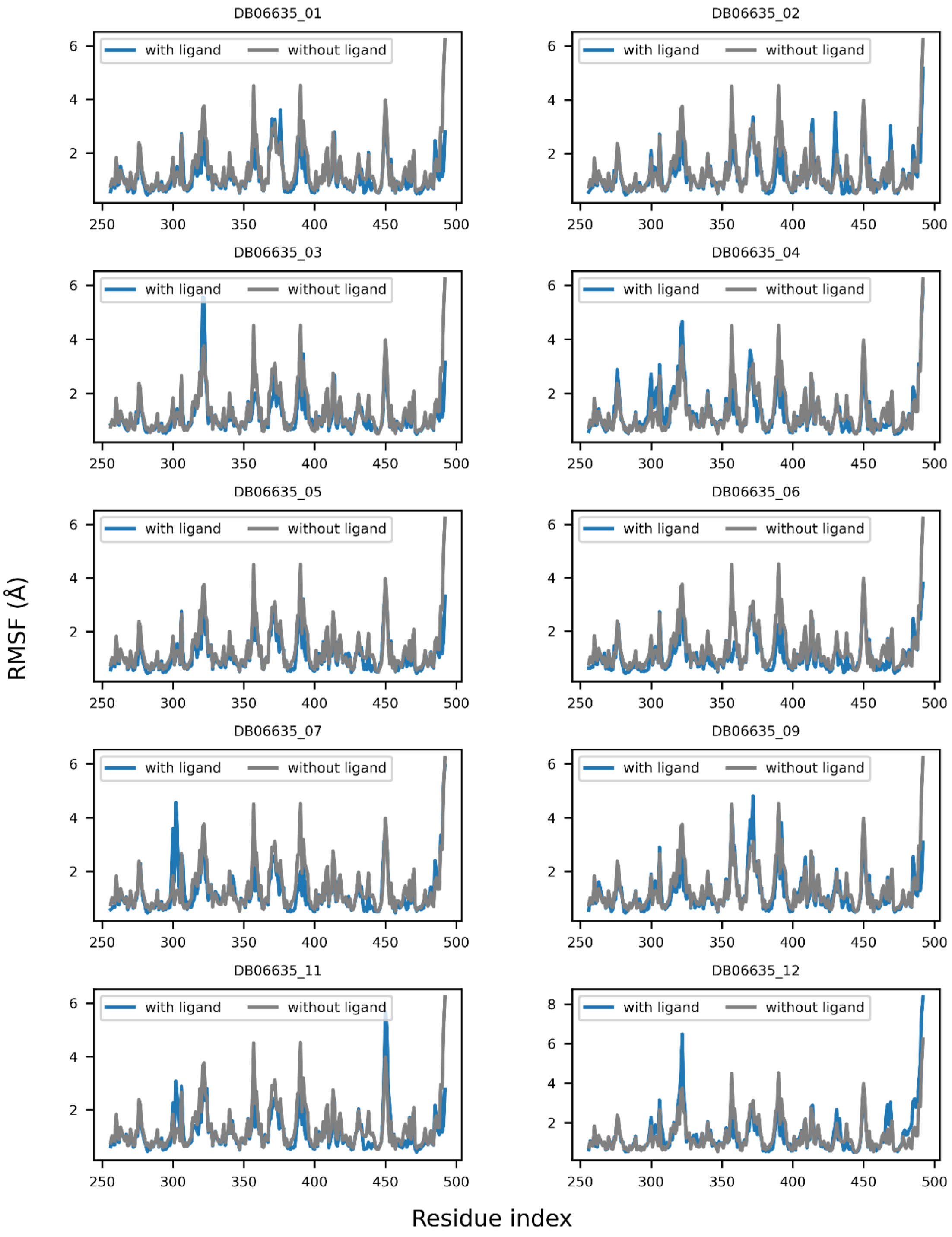

(Figure S5 continued)

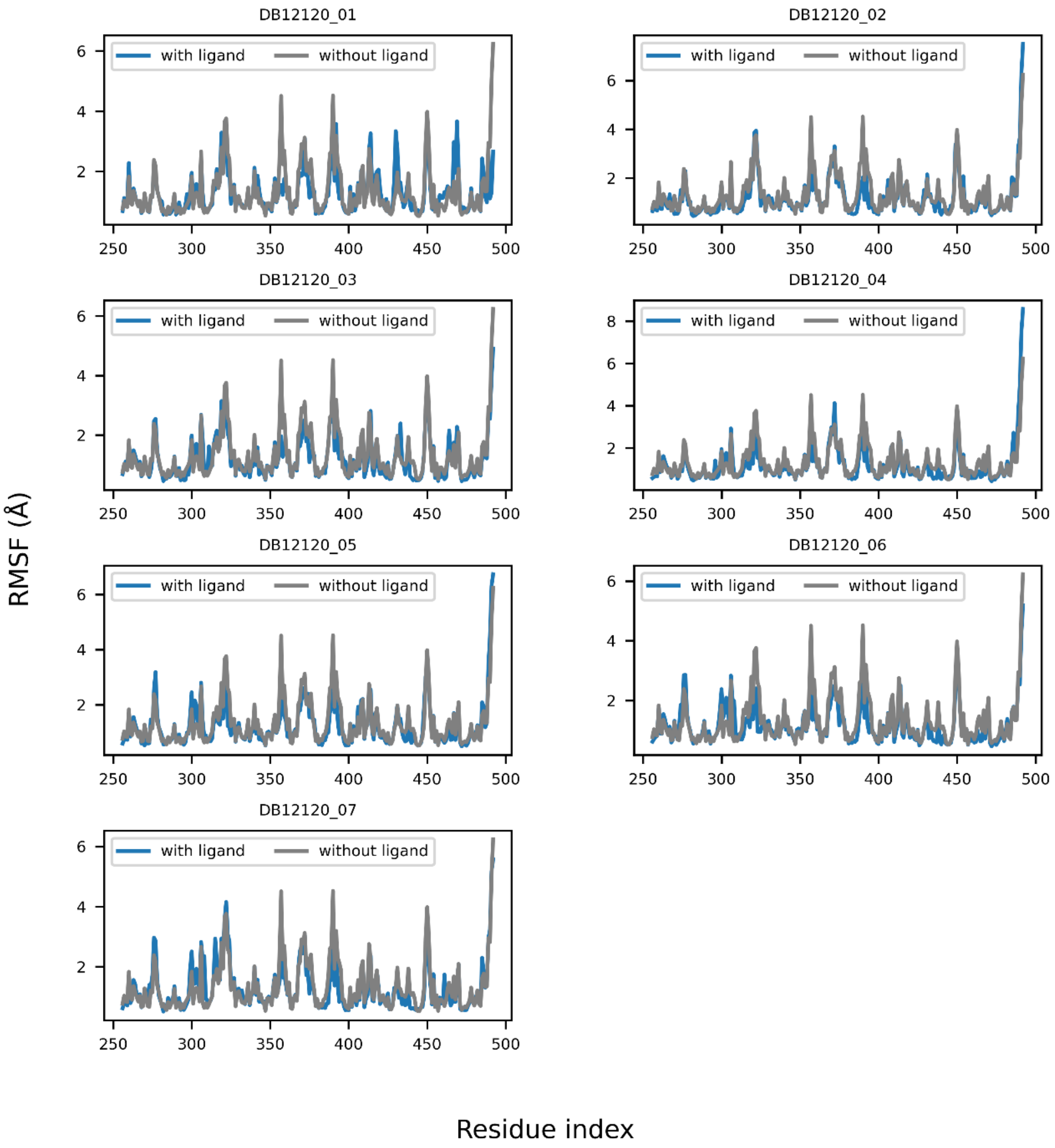

(Figure S5 continued)

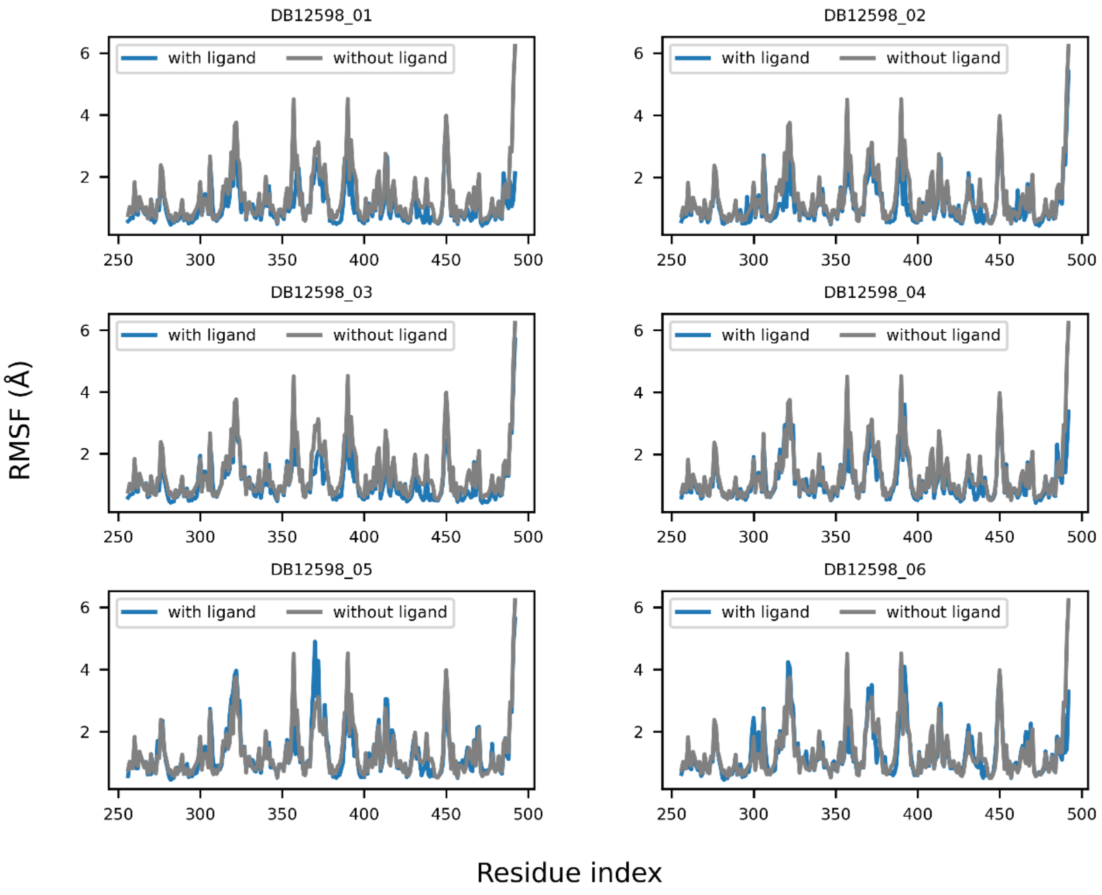

(Figure S5 continued)

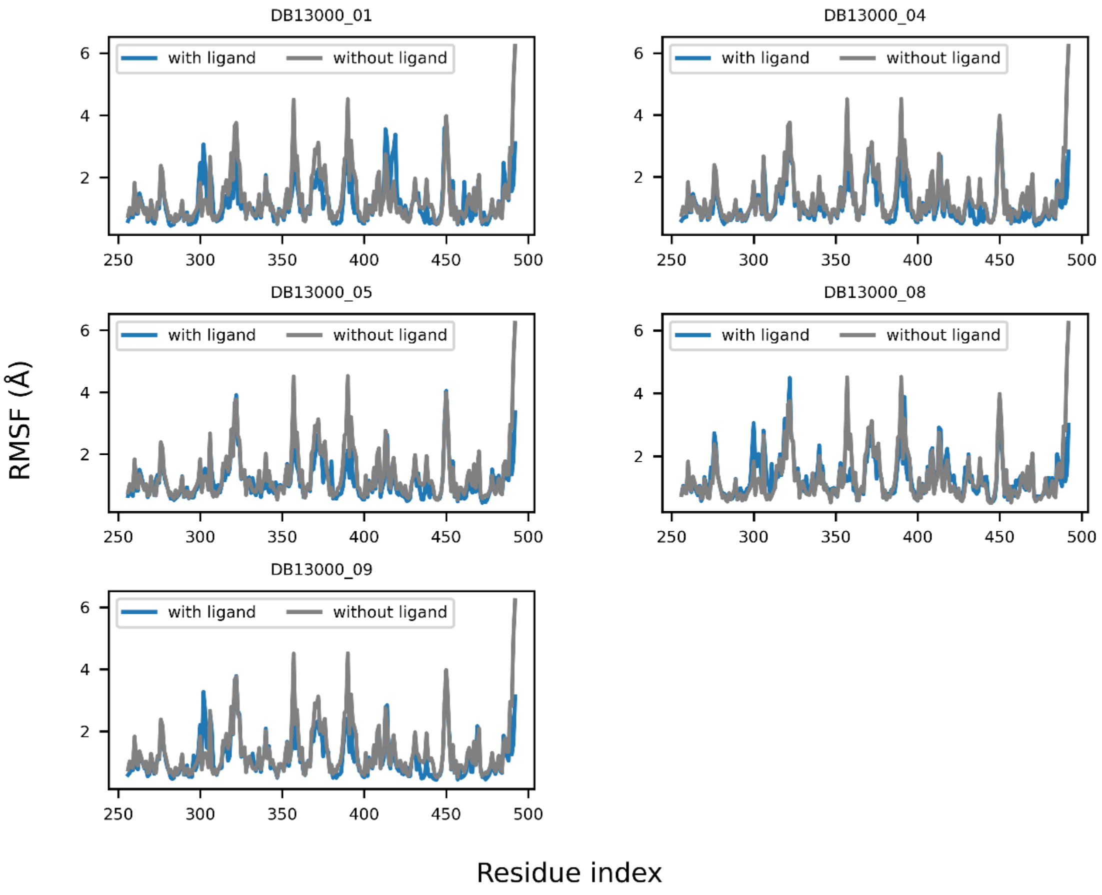

(Figure S5 continued)

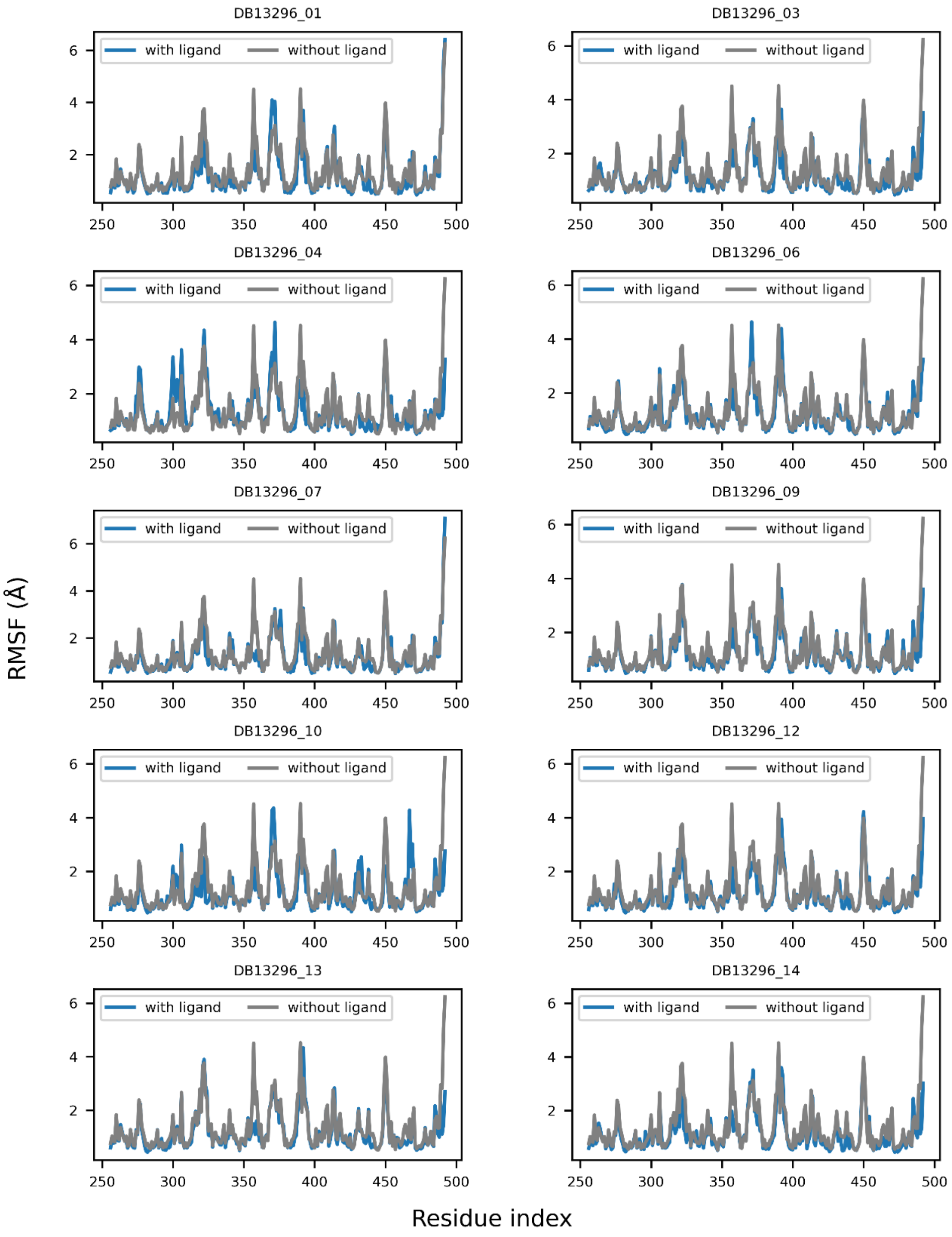

(Figure S5 continued)

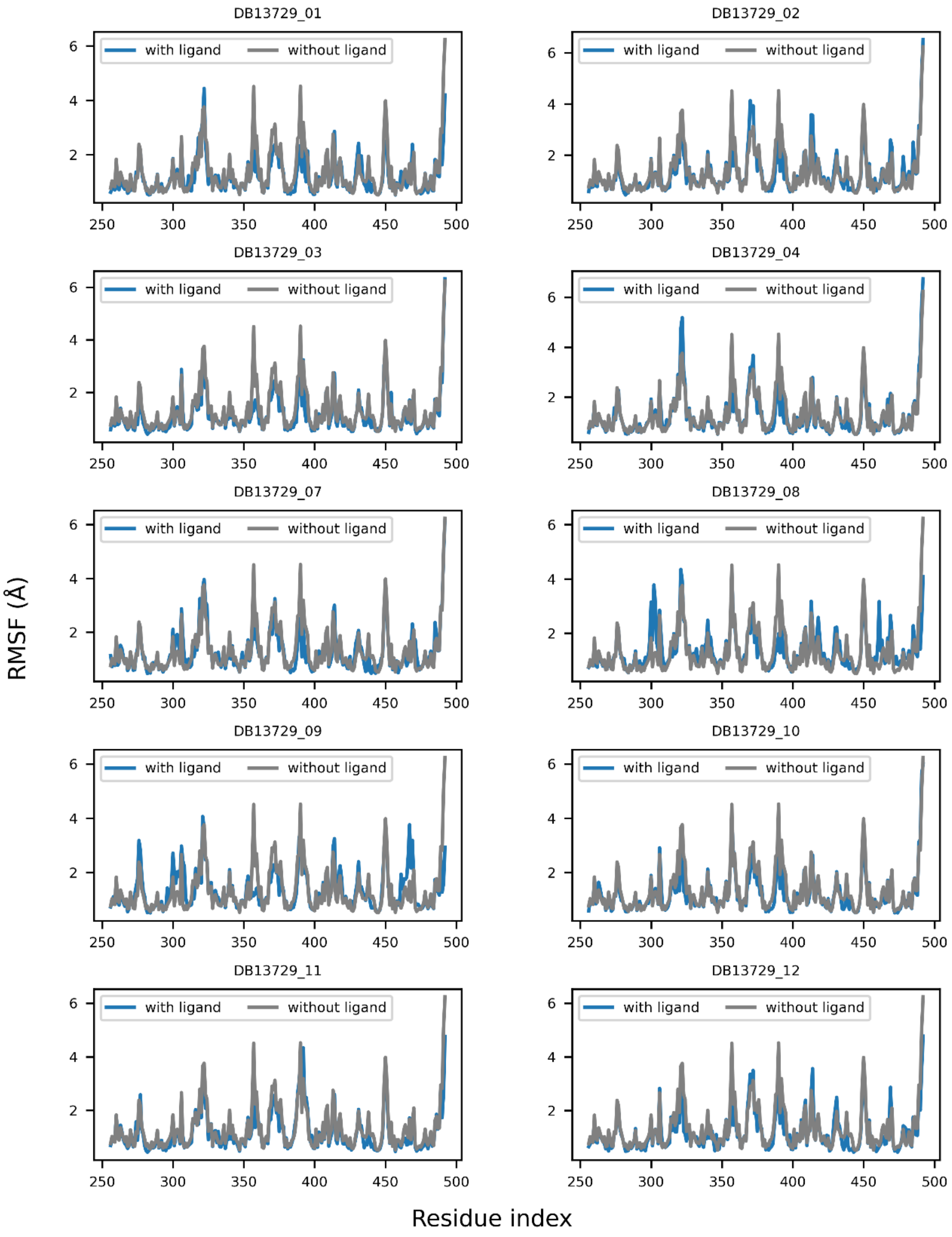

(Figure S5 continued)

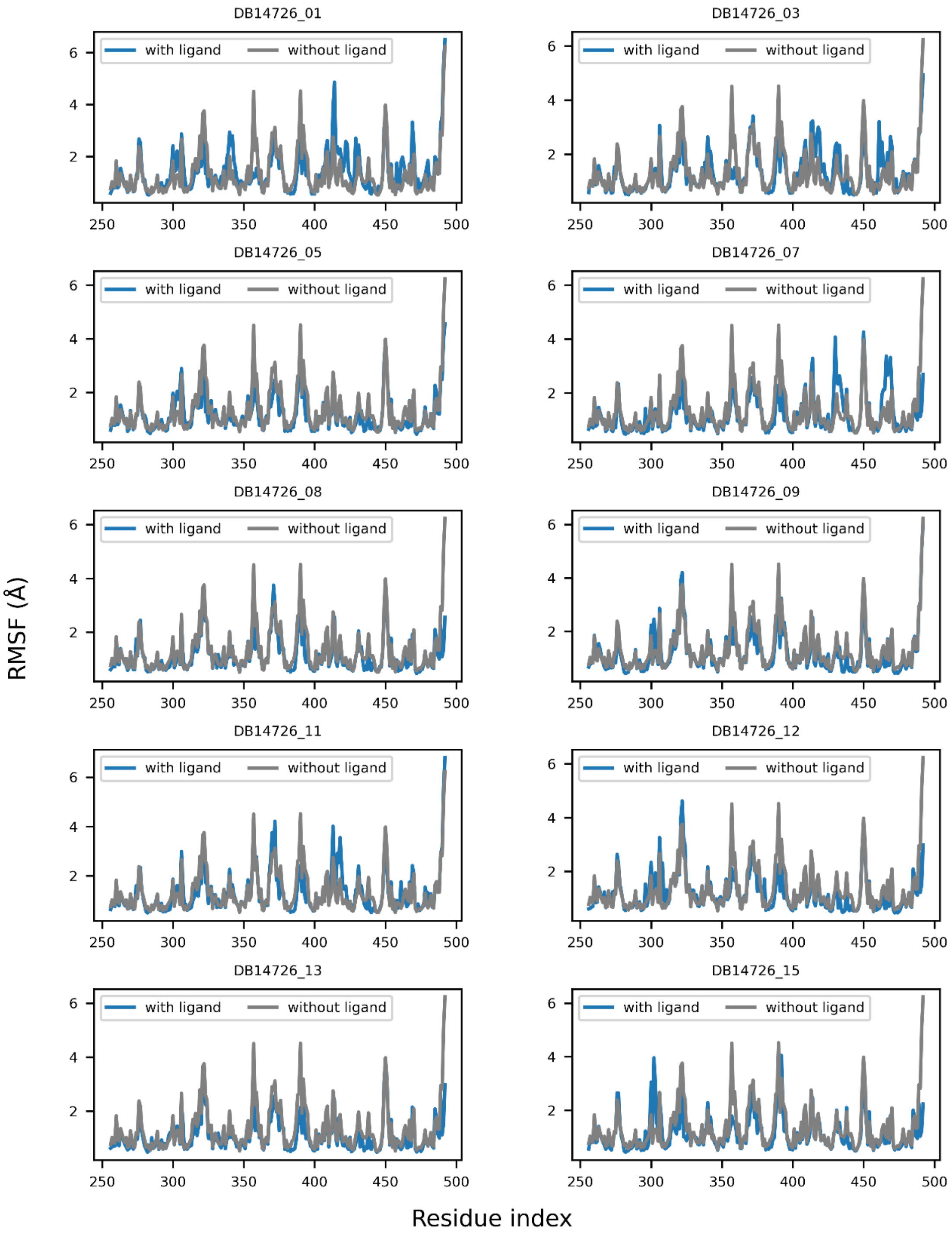

(Figure S5 continued)

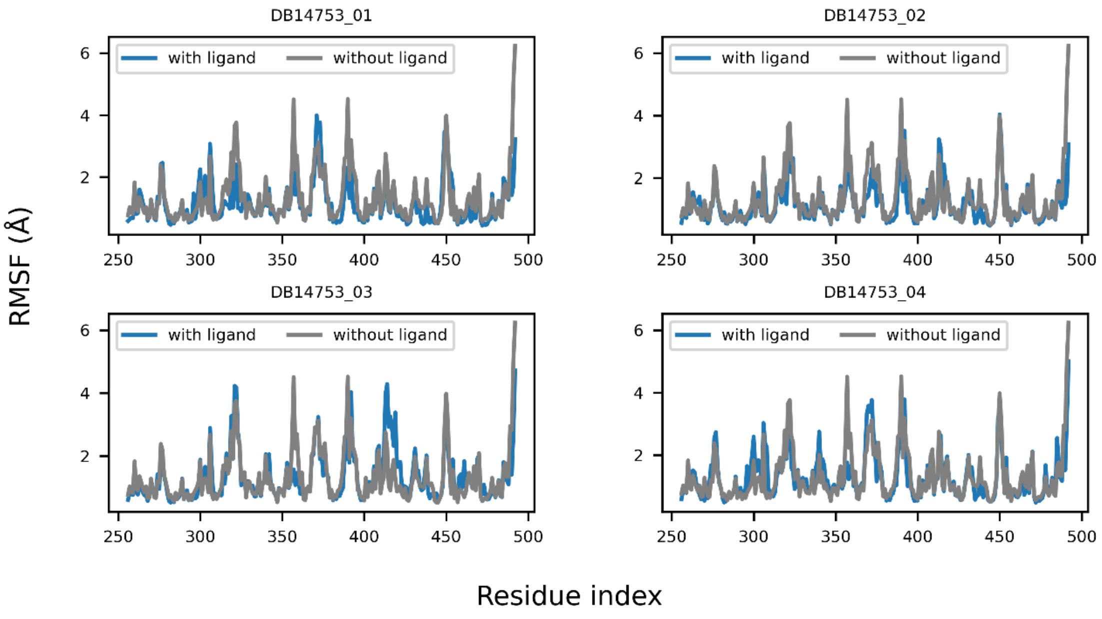

(Figure S5 continued)

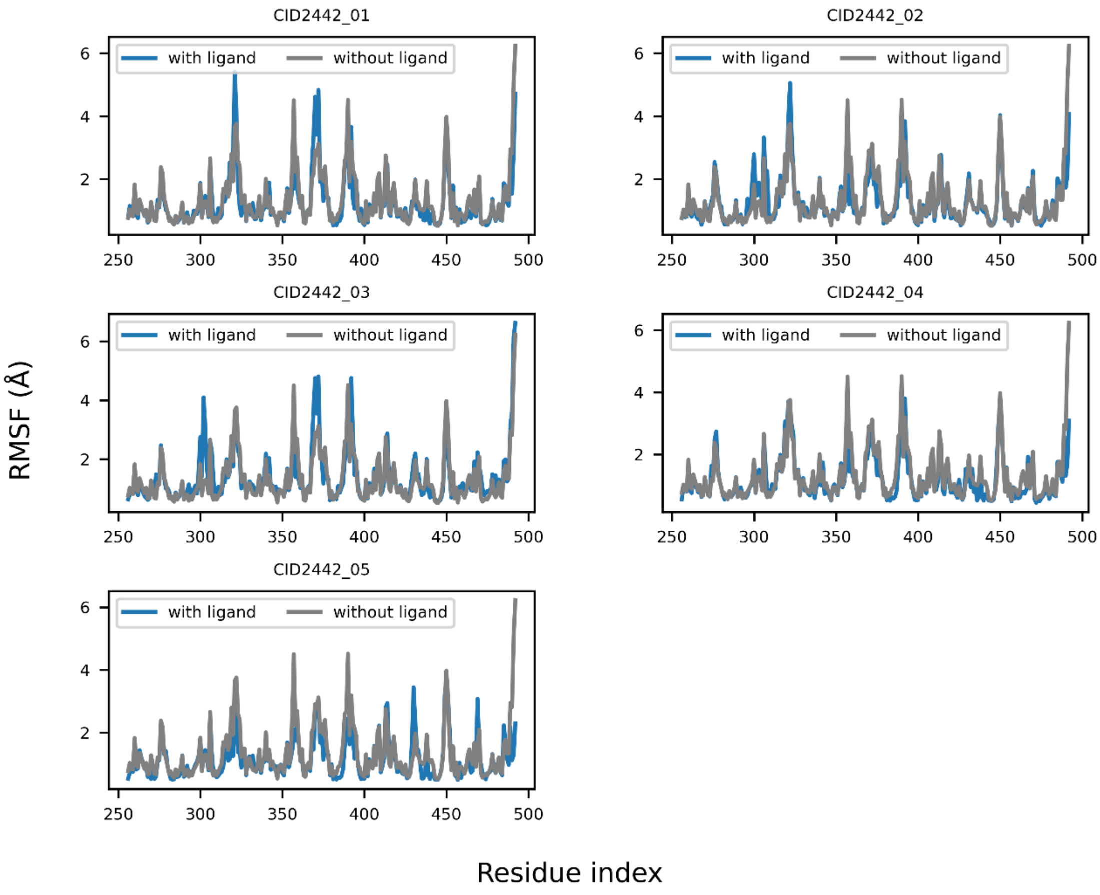

(Figure S5 continued)

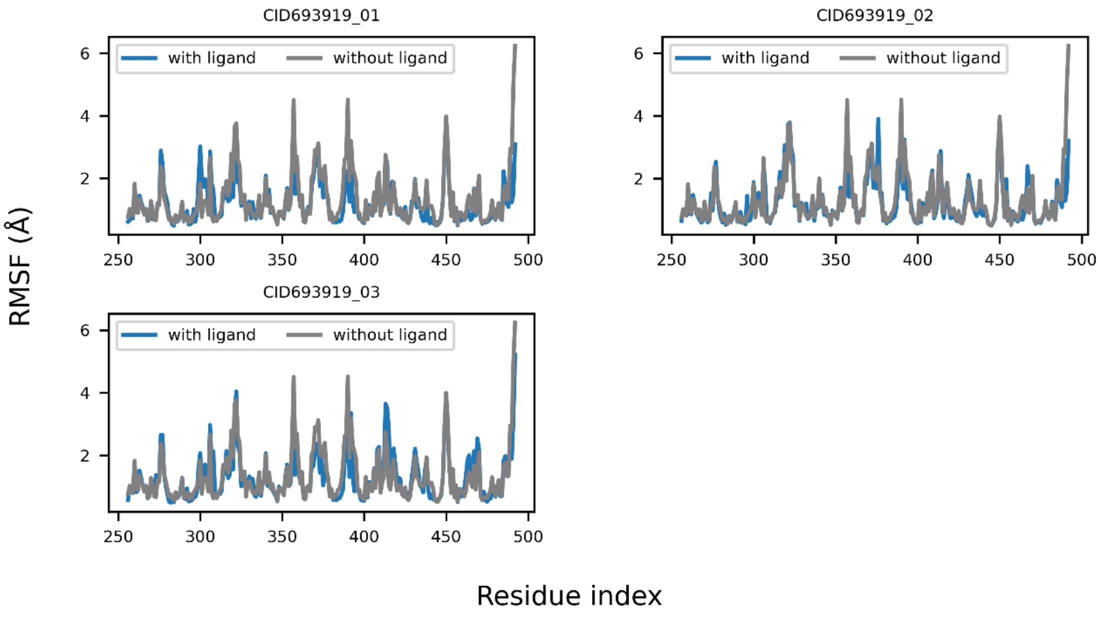

(Figure S5 continued)

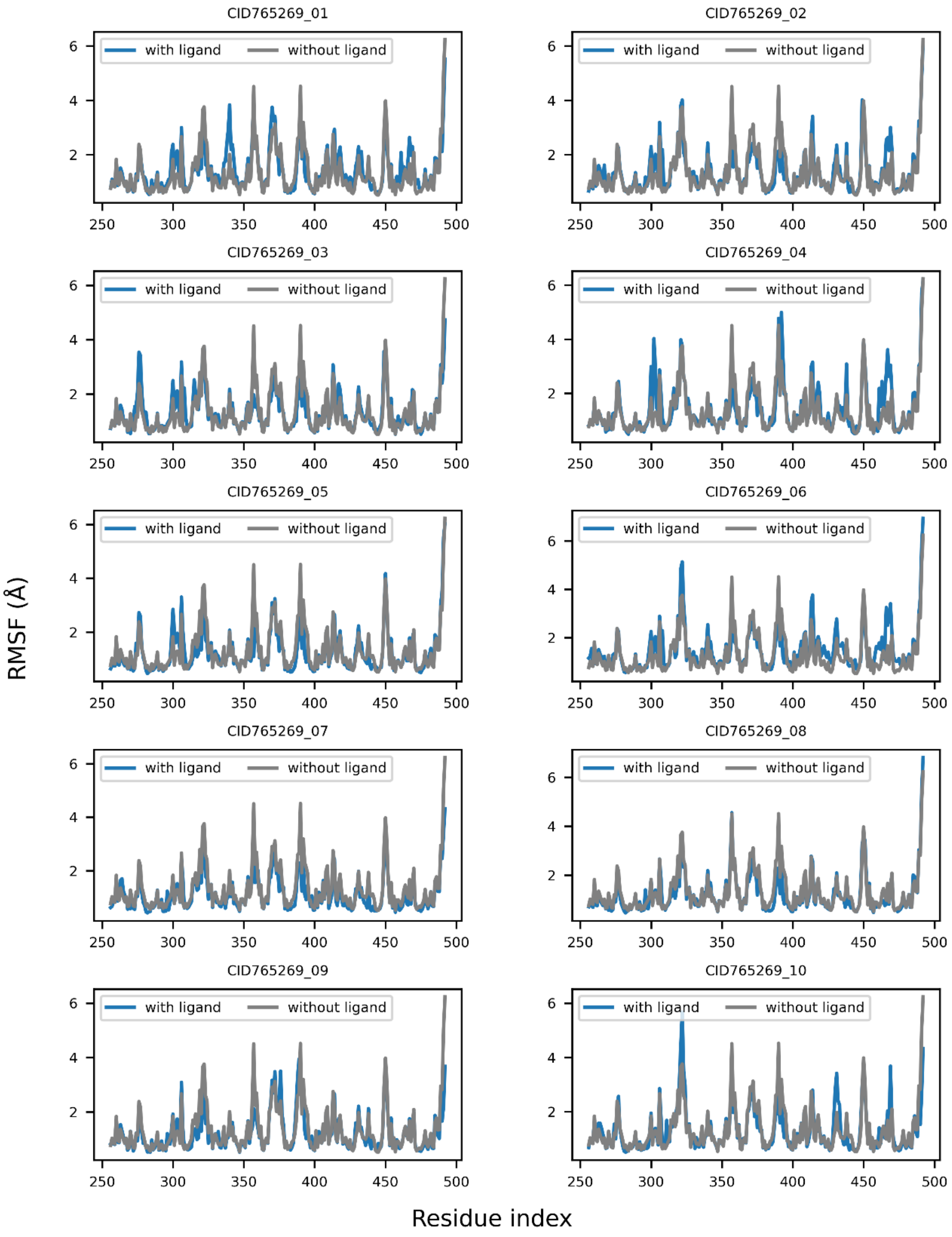

(Figure S5 continued)

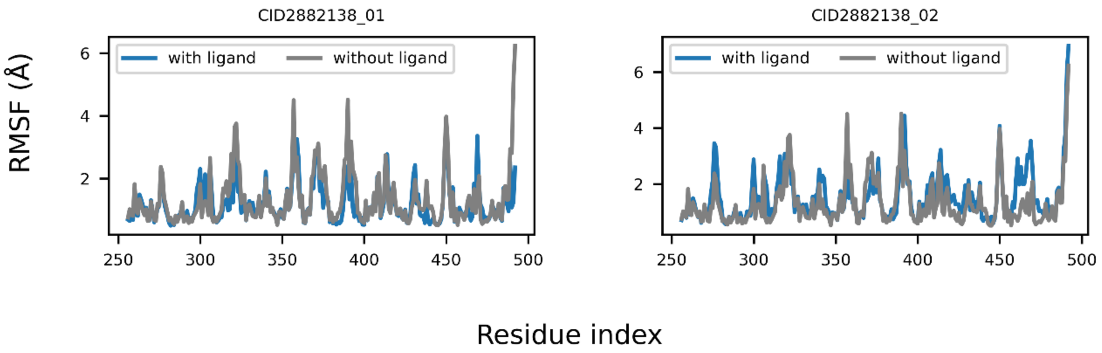

(Figure S5 continued)

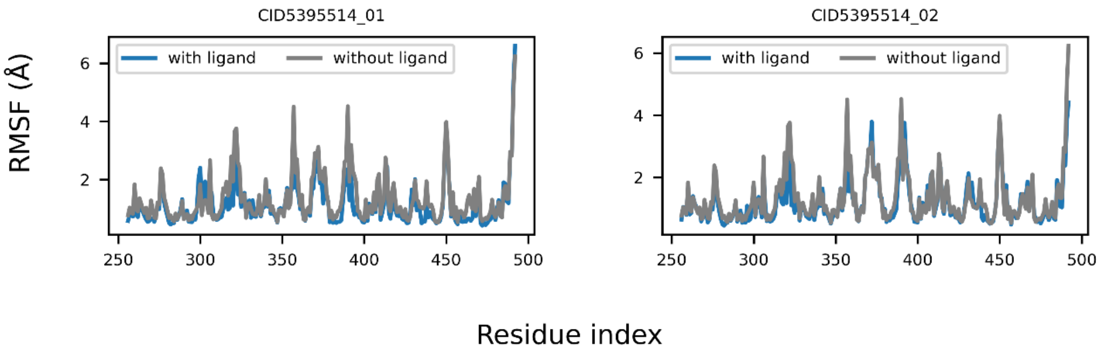

(Figure S5 continued)

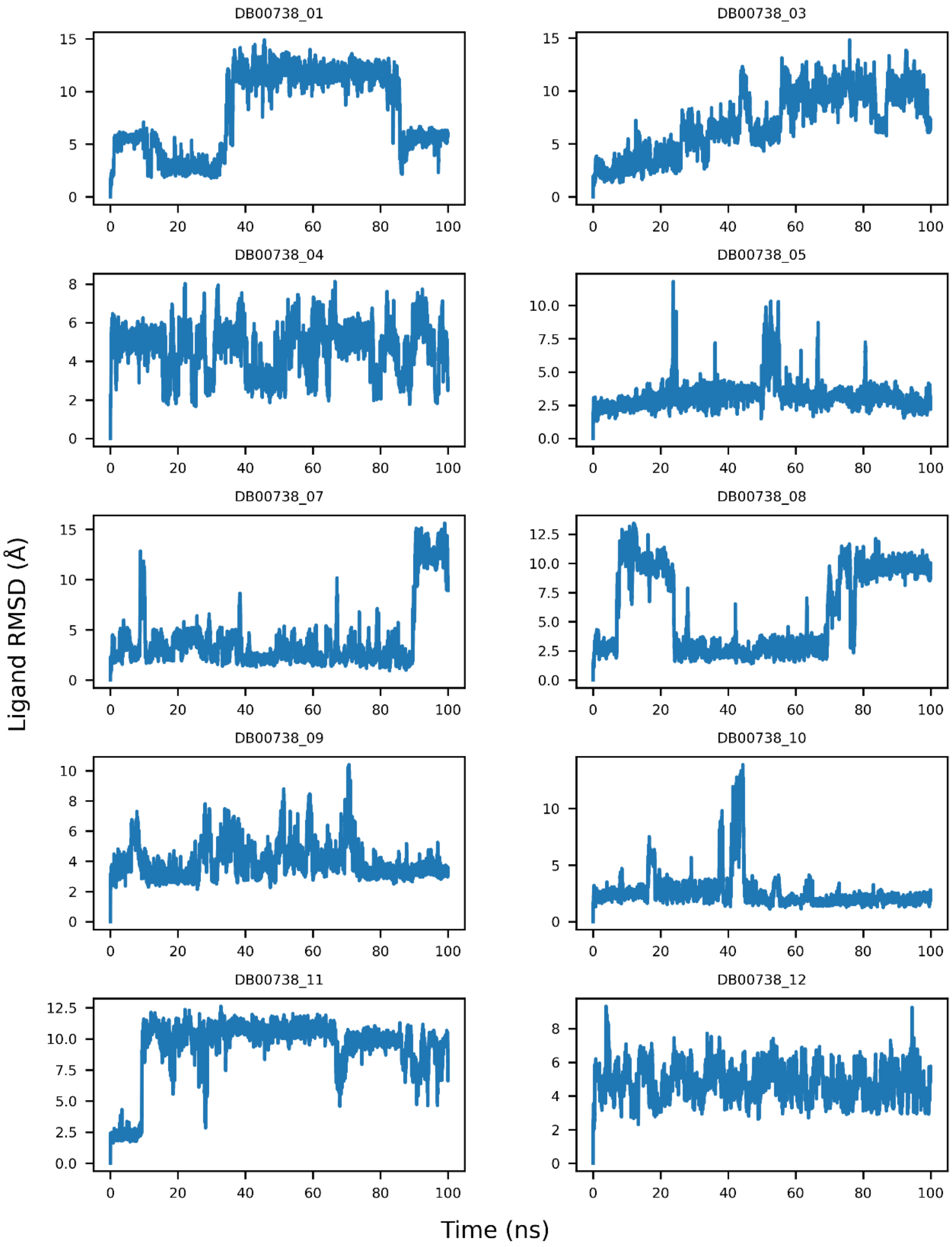

Figure S6. Ligand RMSD calculated from 100 ns molecular dynamics simulations for each compound with different poses.

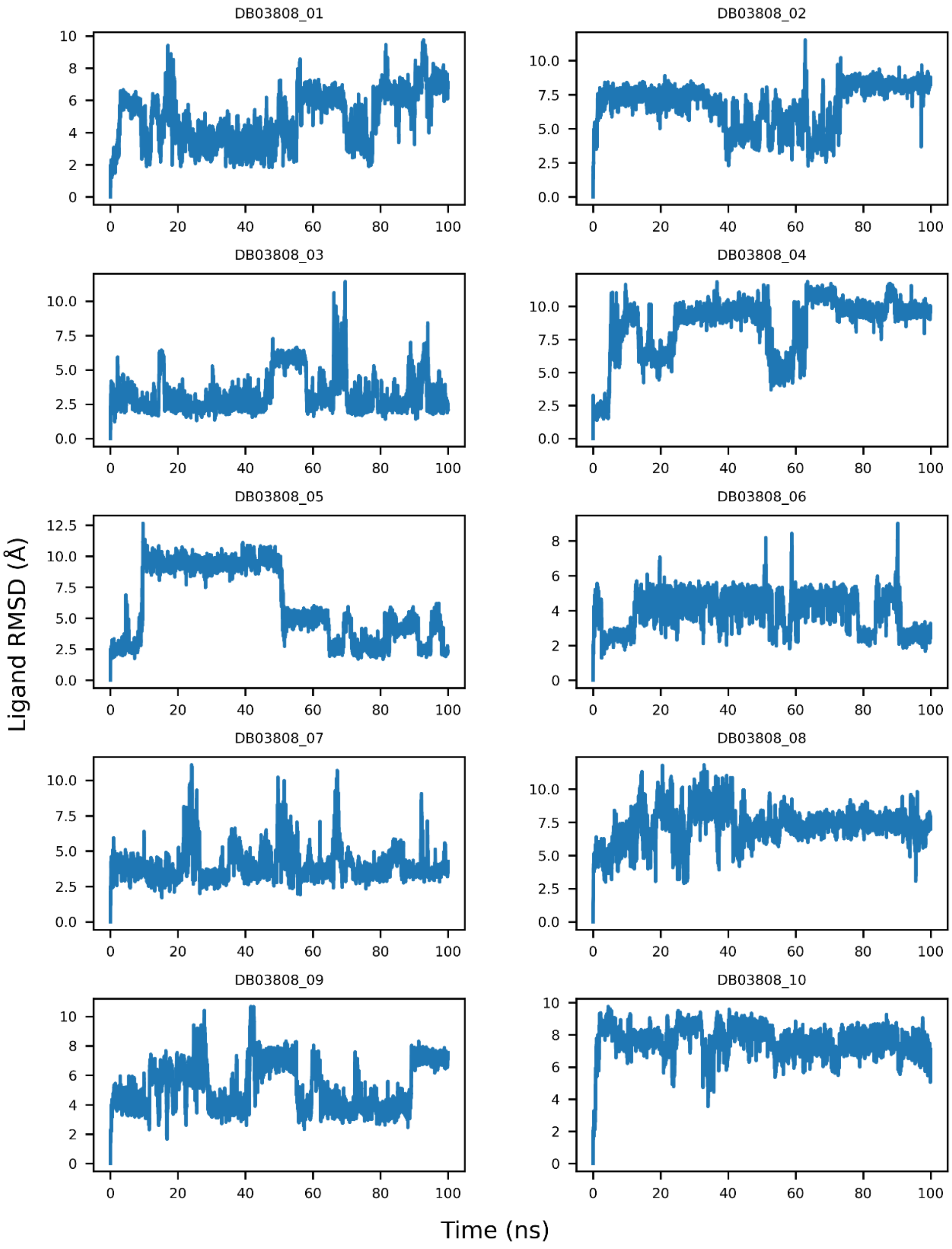

(Figure S6 continued)

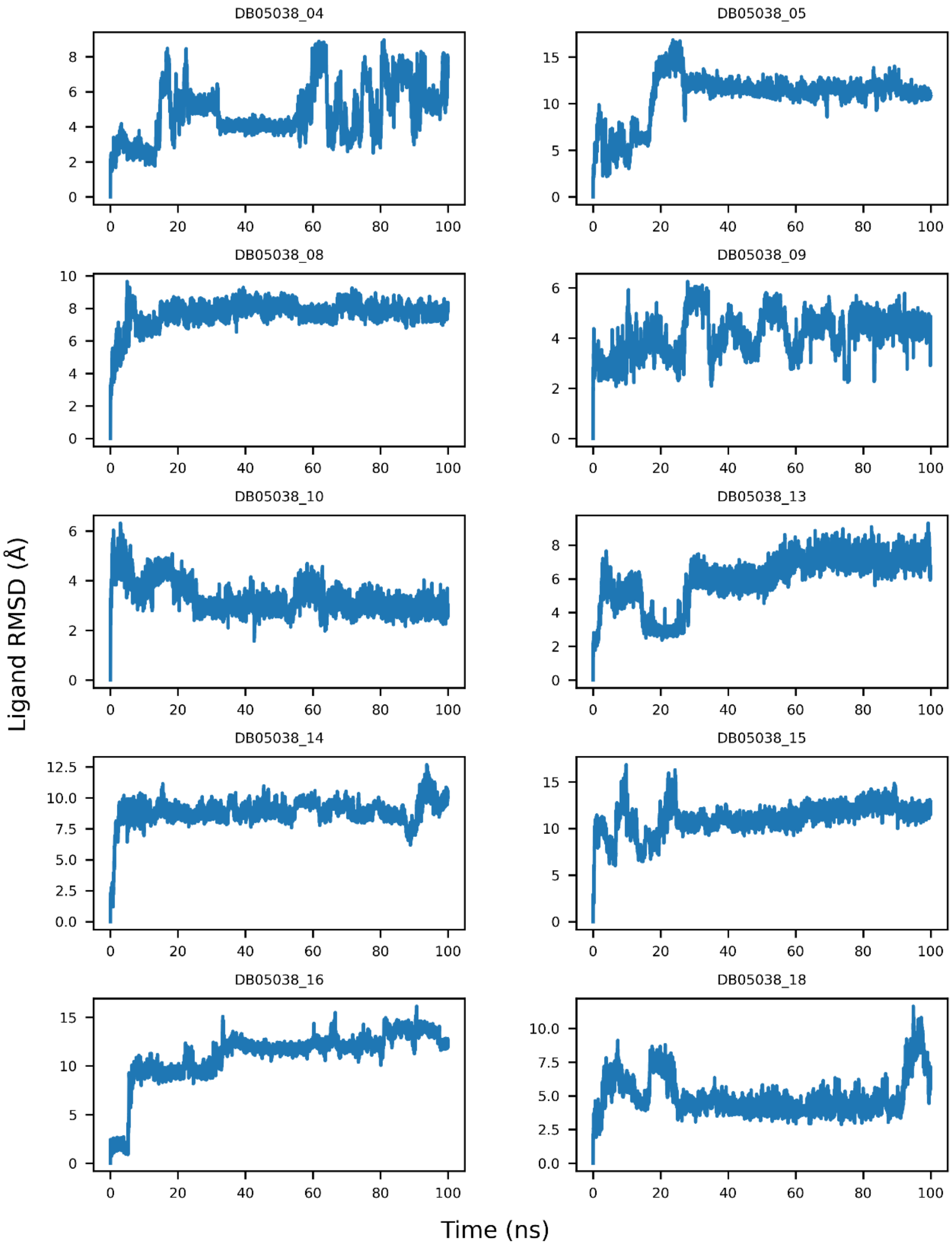

(Figure S6 continued)

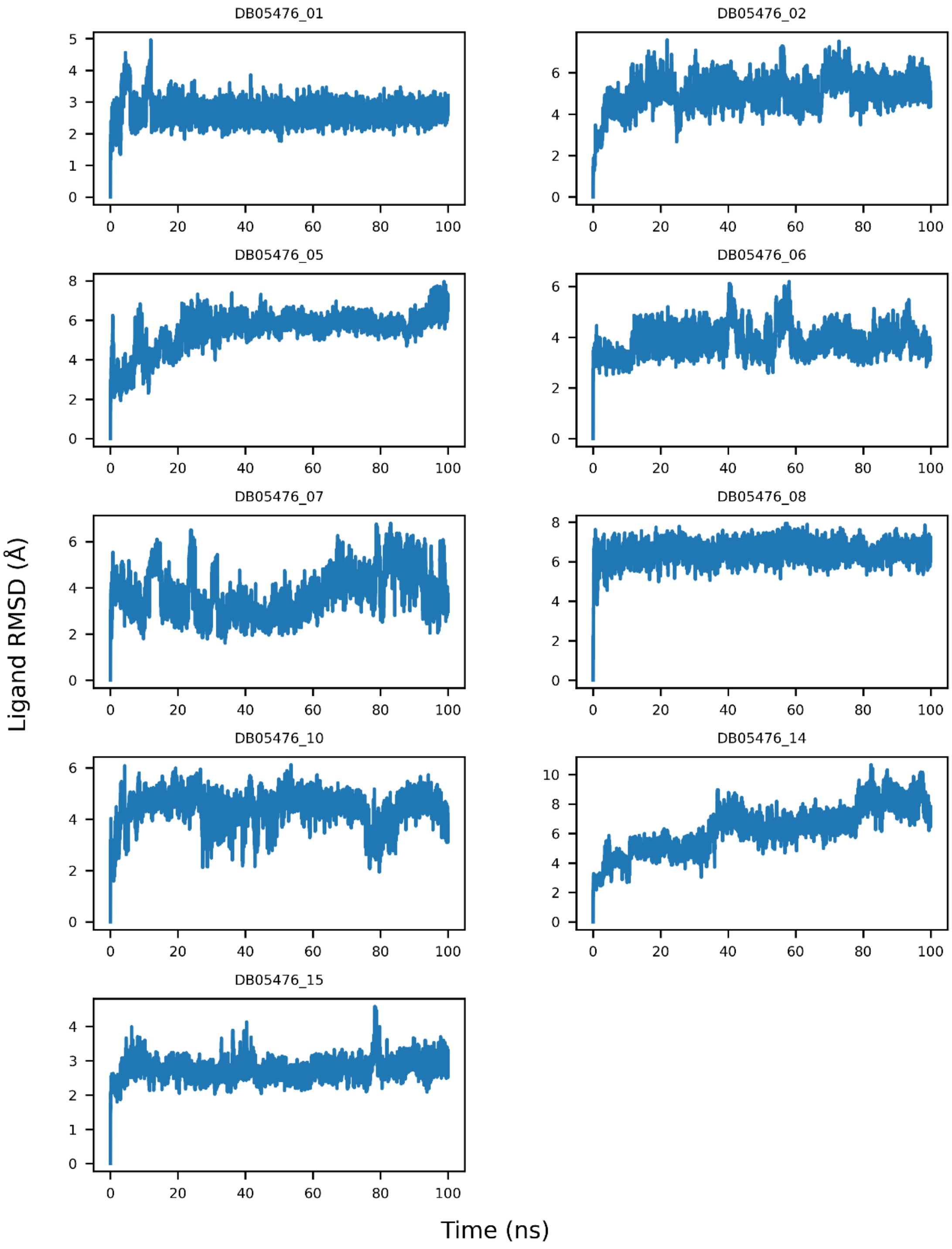

(Figure S6 continued)

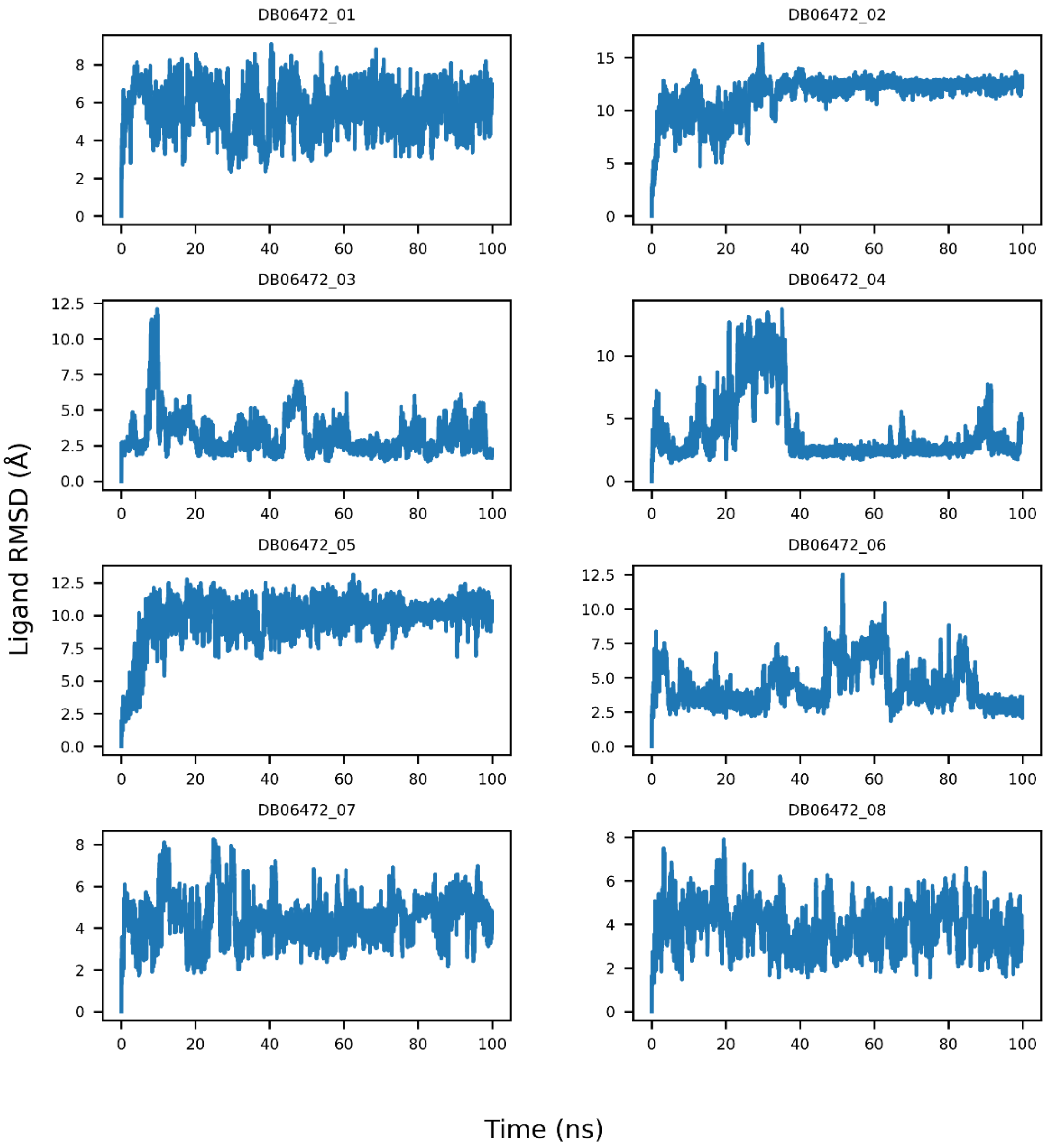

(Figure S6 continued)

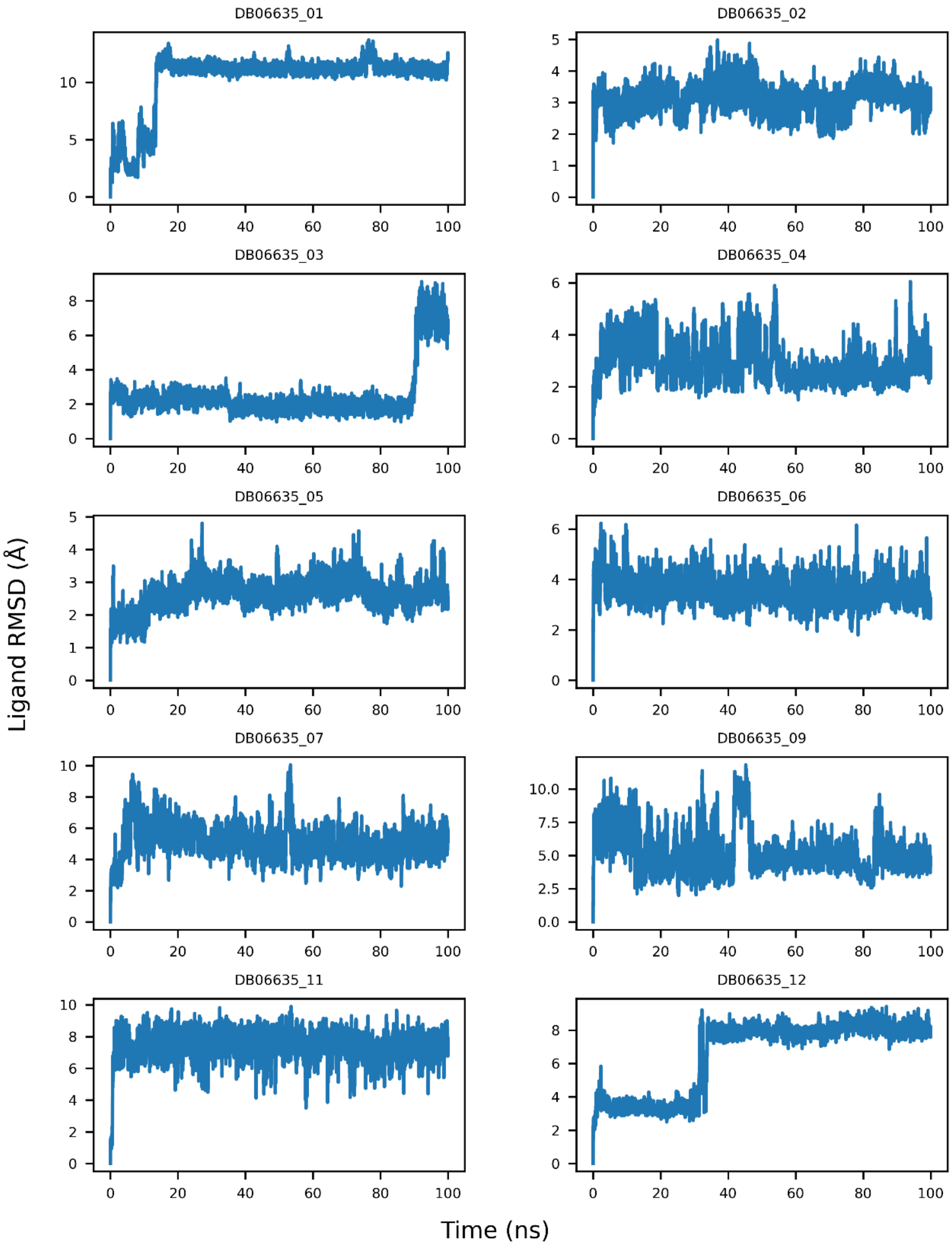

(Figure S6 continued)

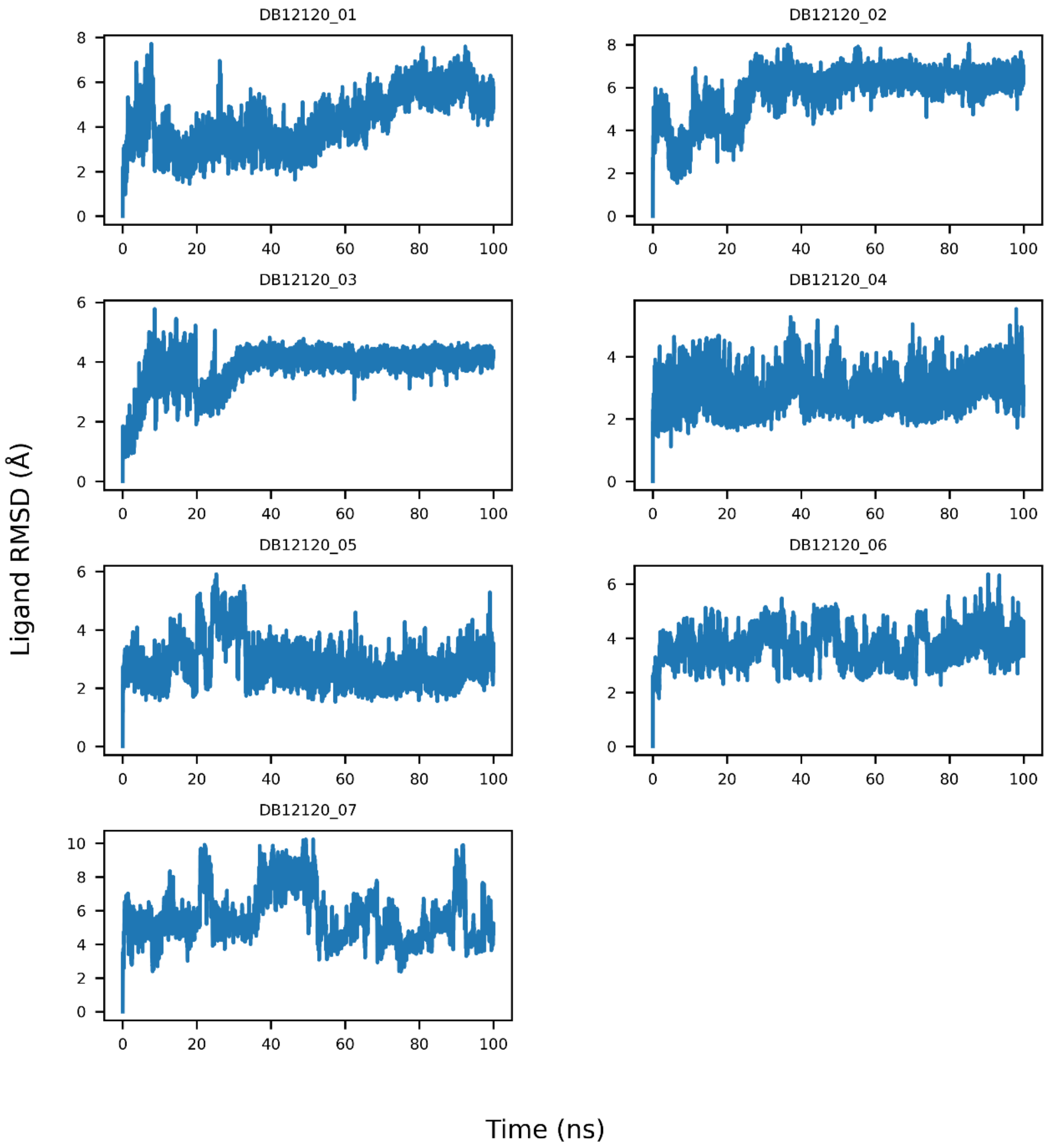

(Figure S6 continued)

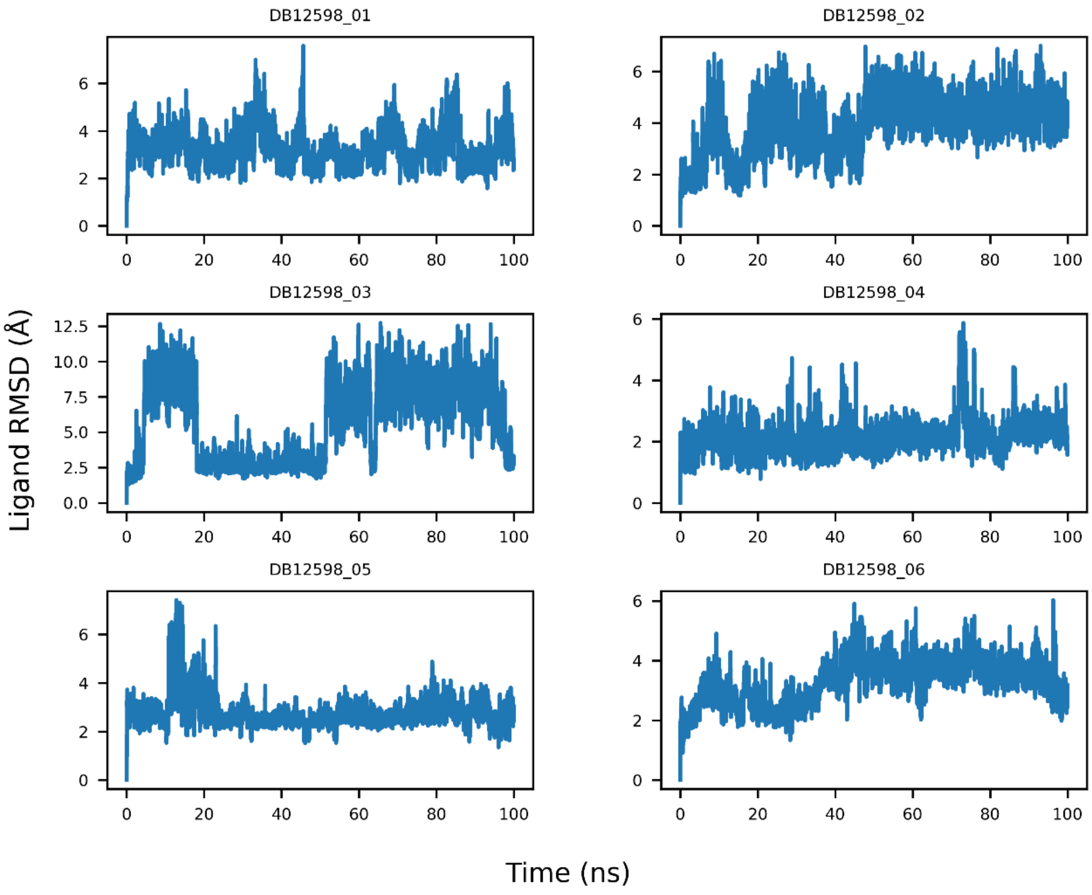

(Figure S6 continued)

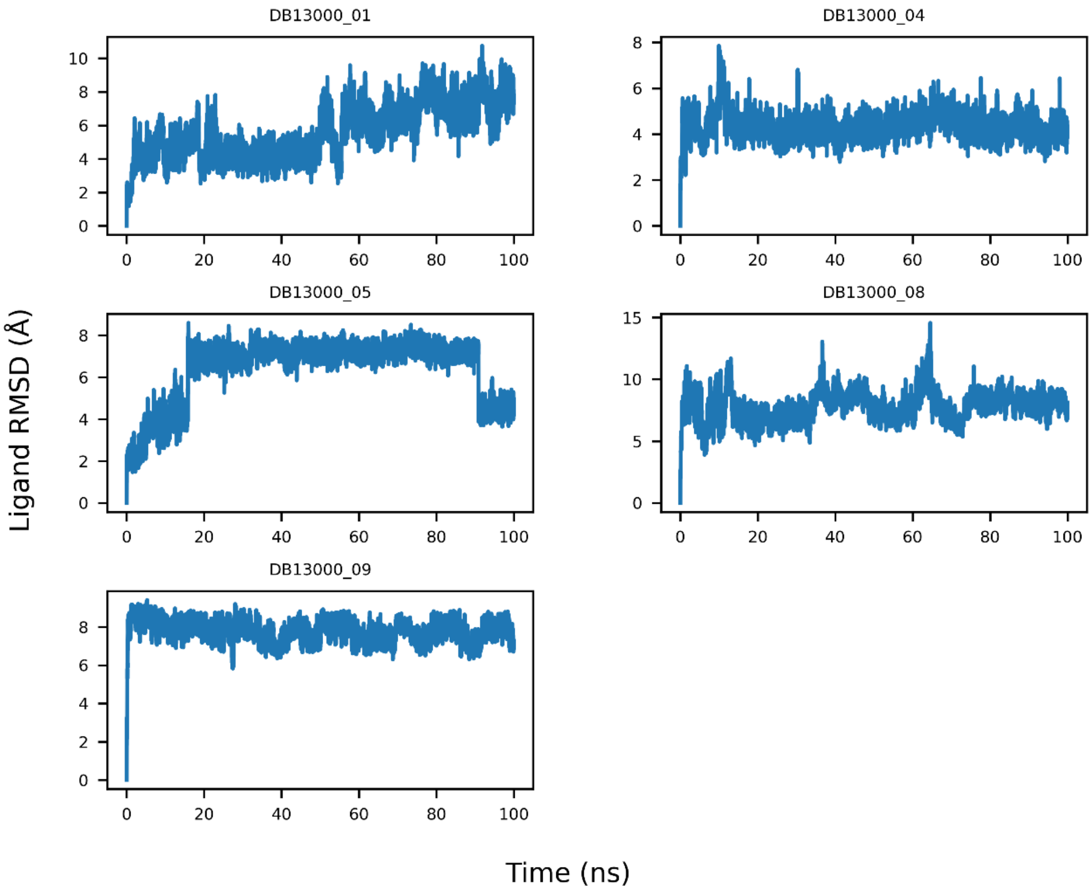

(Figure S6 continued)

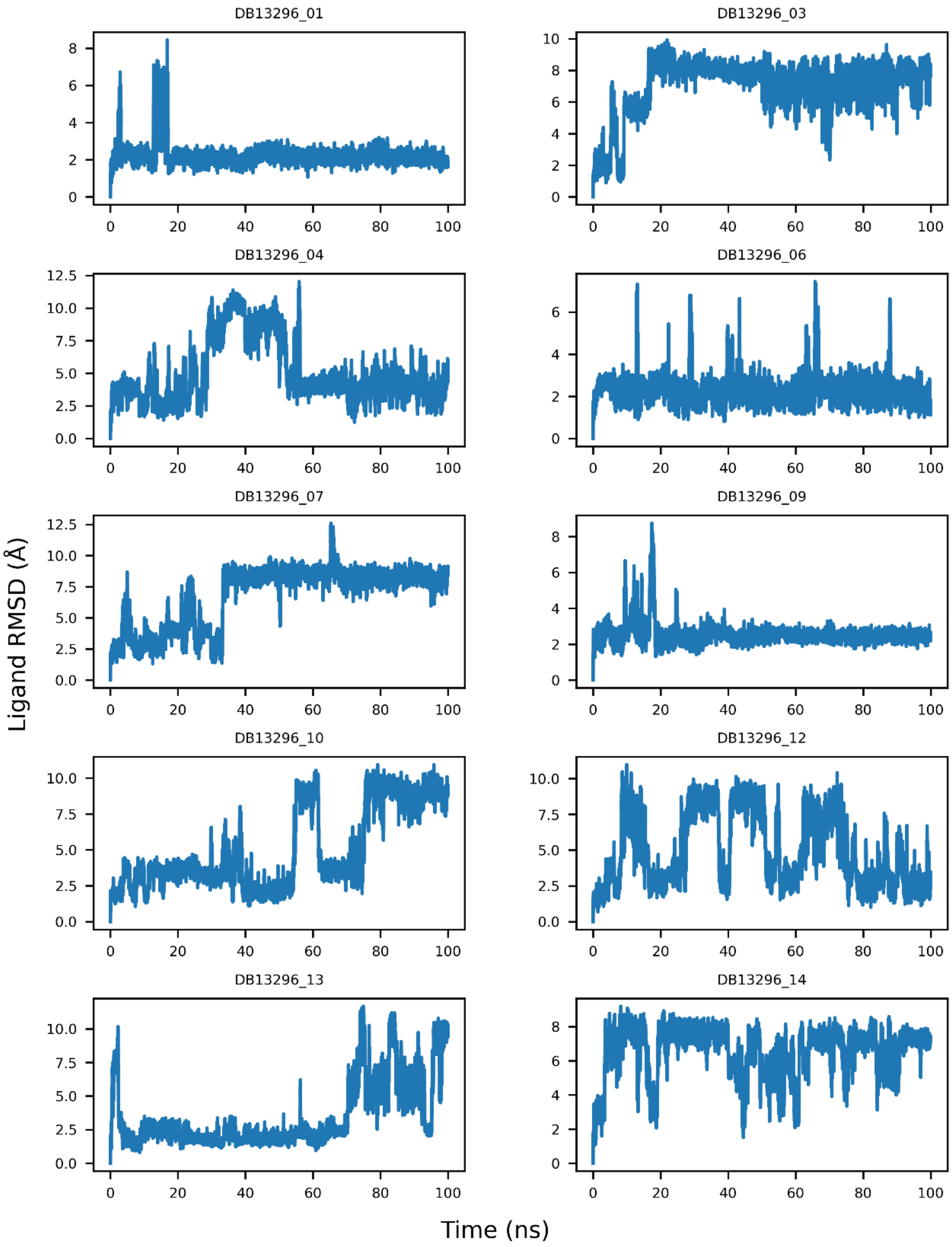

(Figure S6 continued)

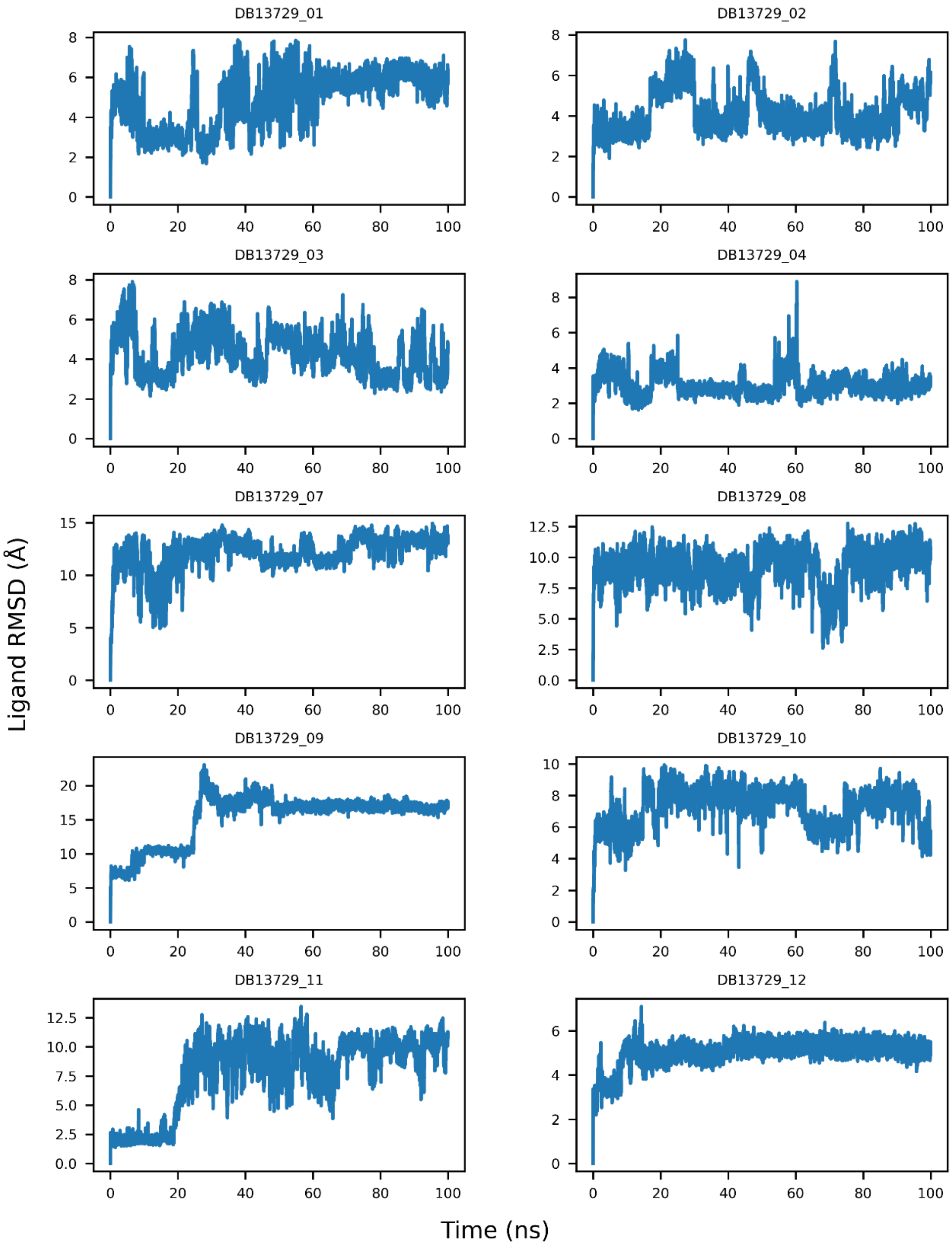

(Figure S6 continued)

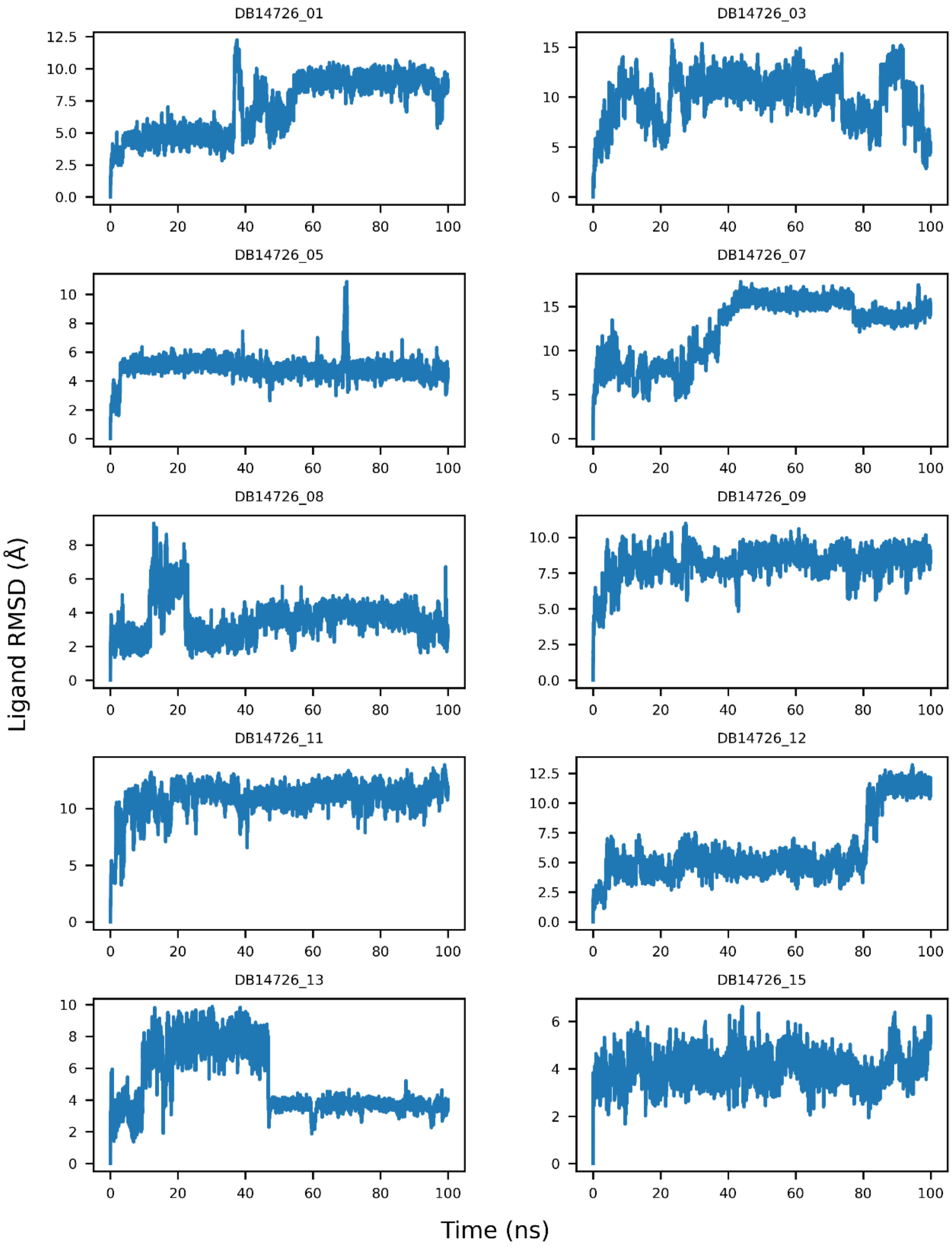

(Figure S6 continued)

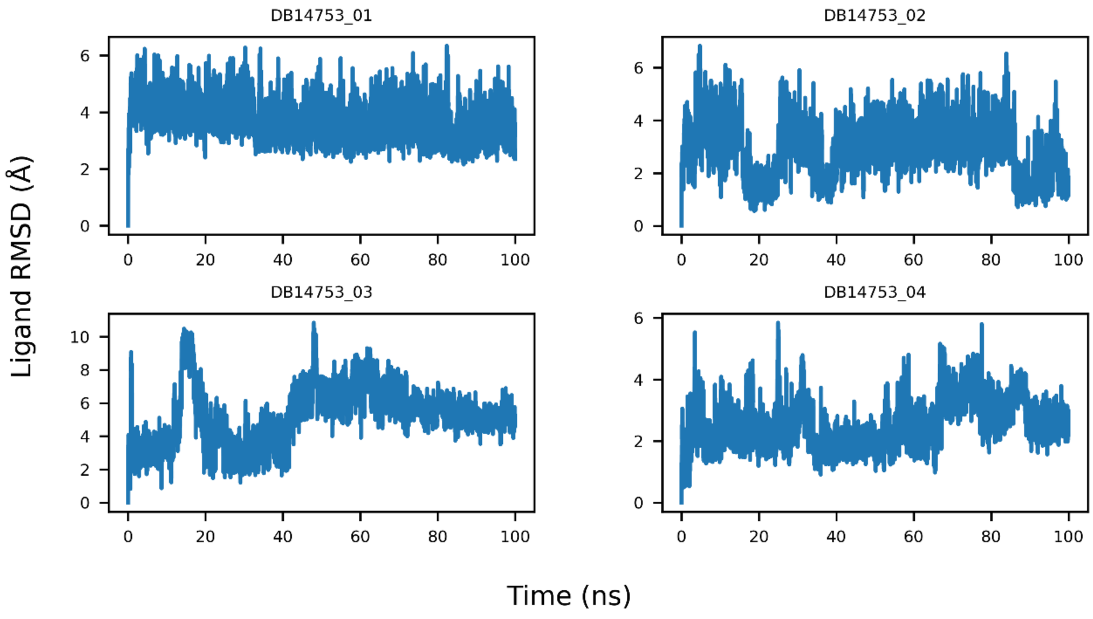

(Figure S6 continued)

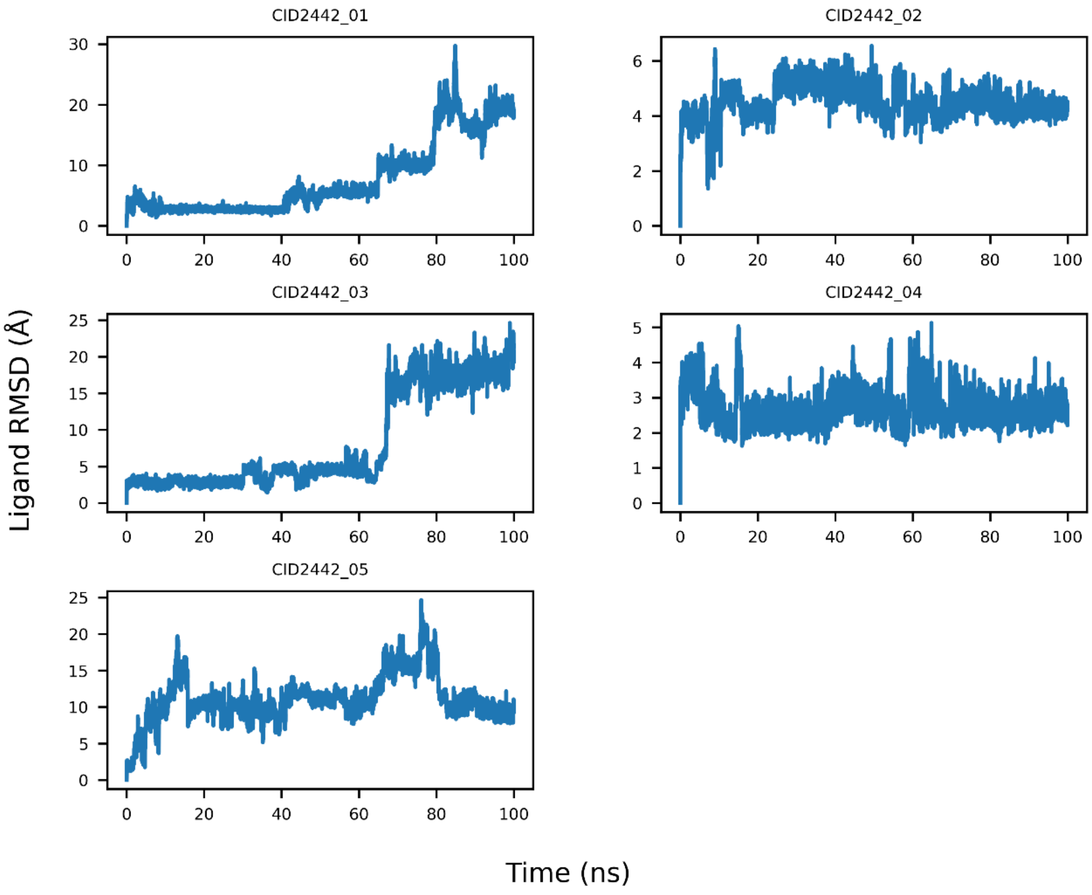

(Figure S6 continued)

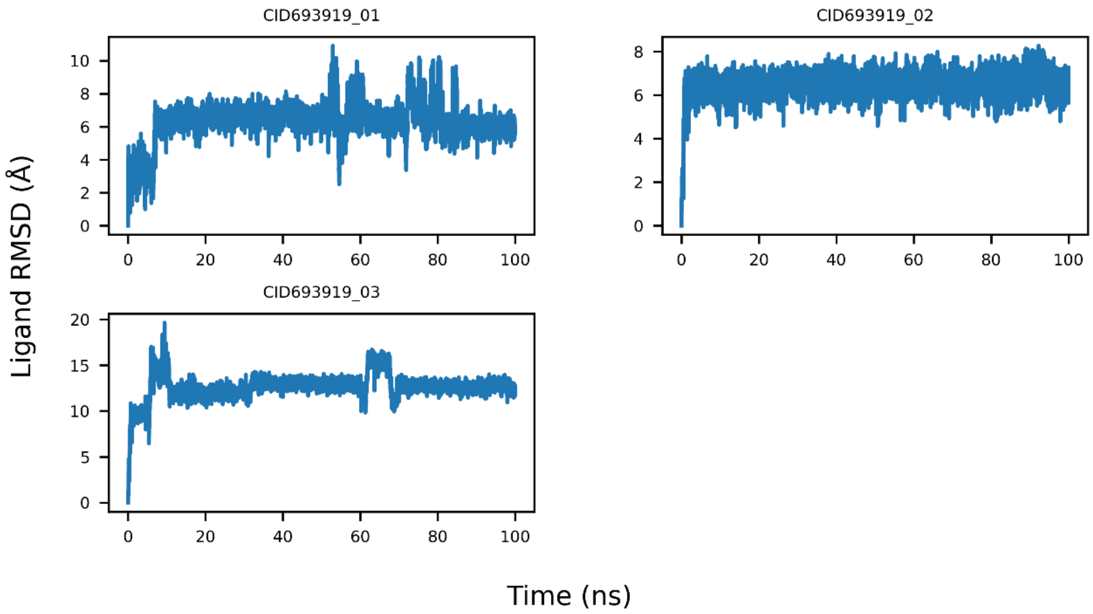

(Figure S6 continued)

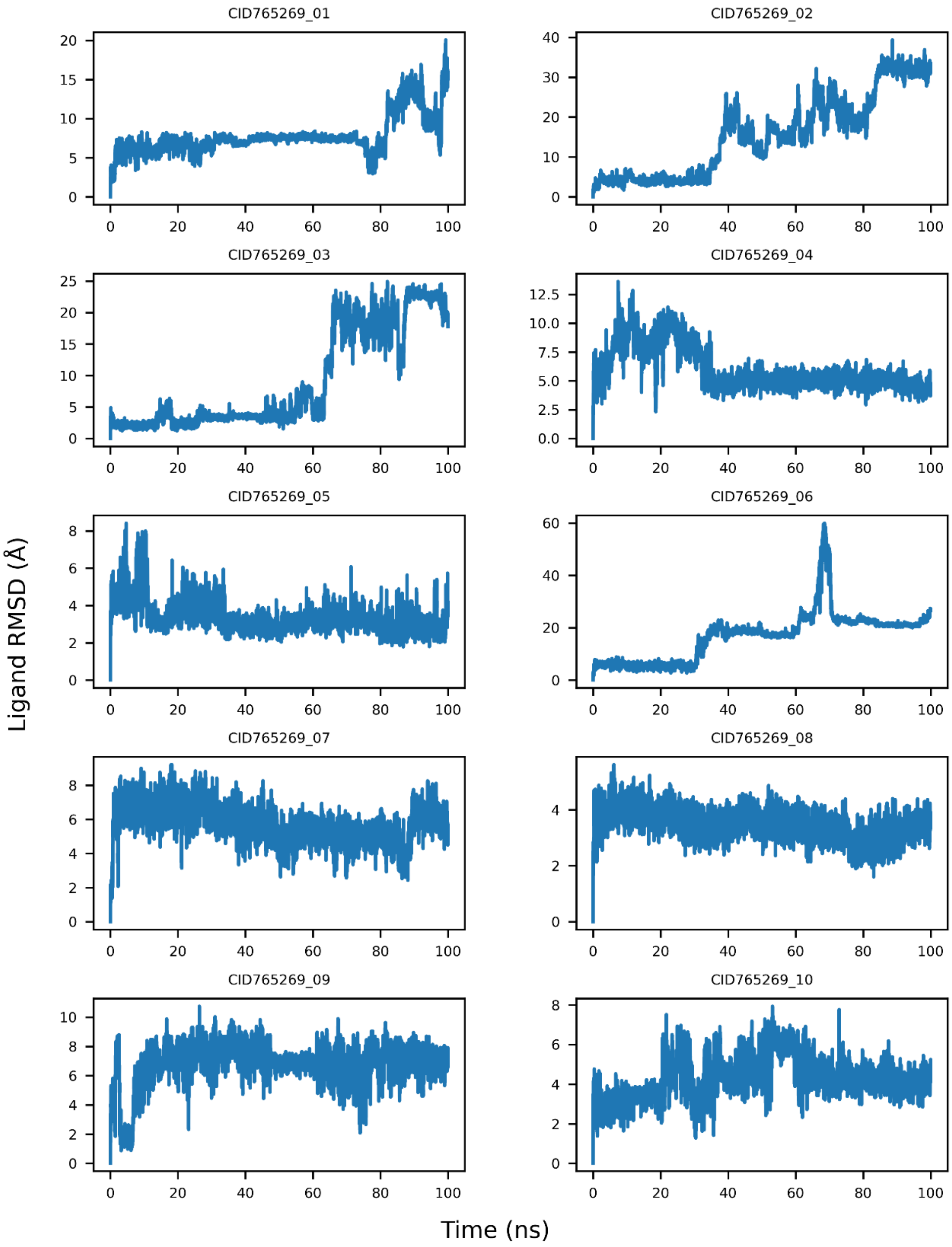

(Figure S6 continued)

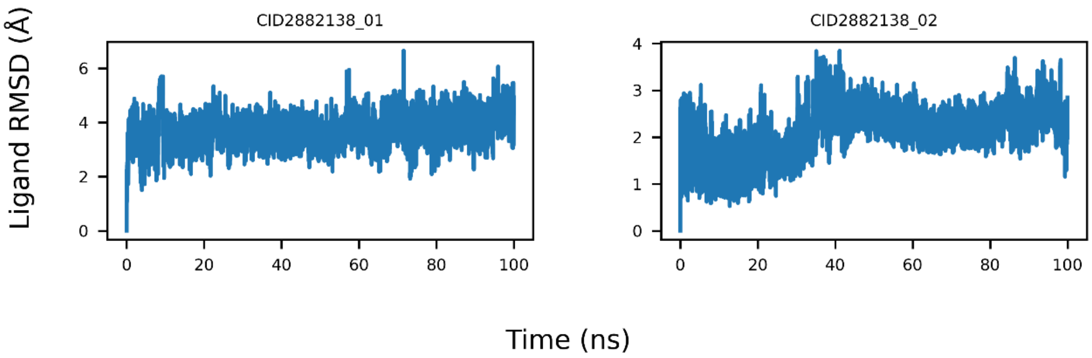

(Figure S6 continued)

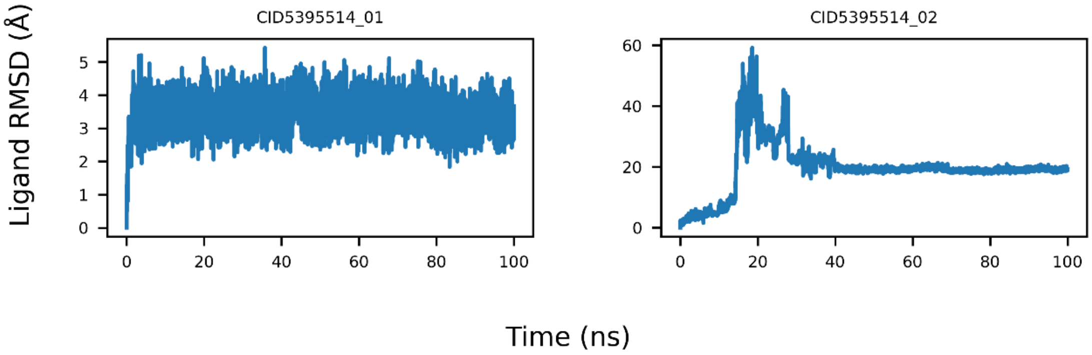

(Figure S6 continued)

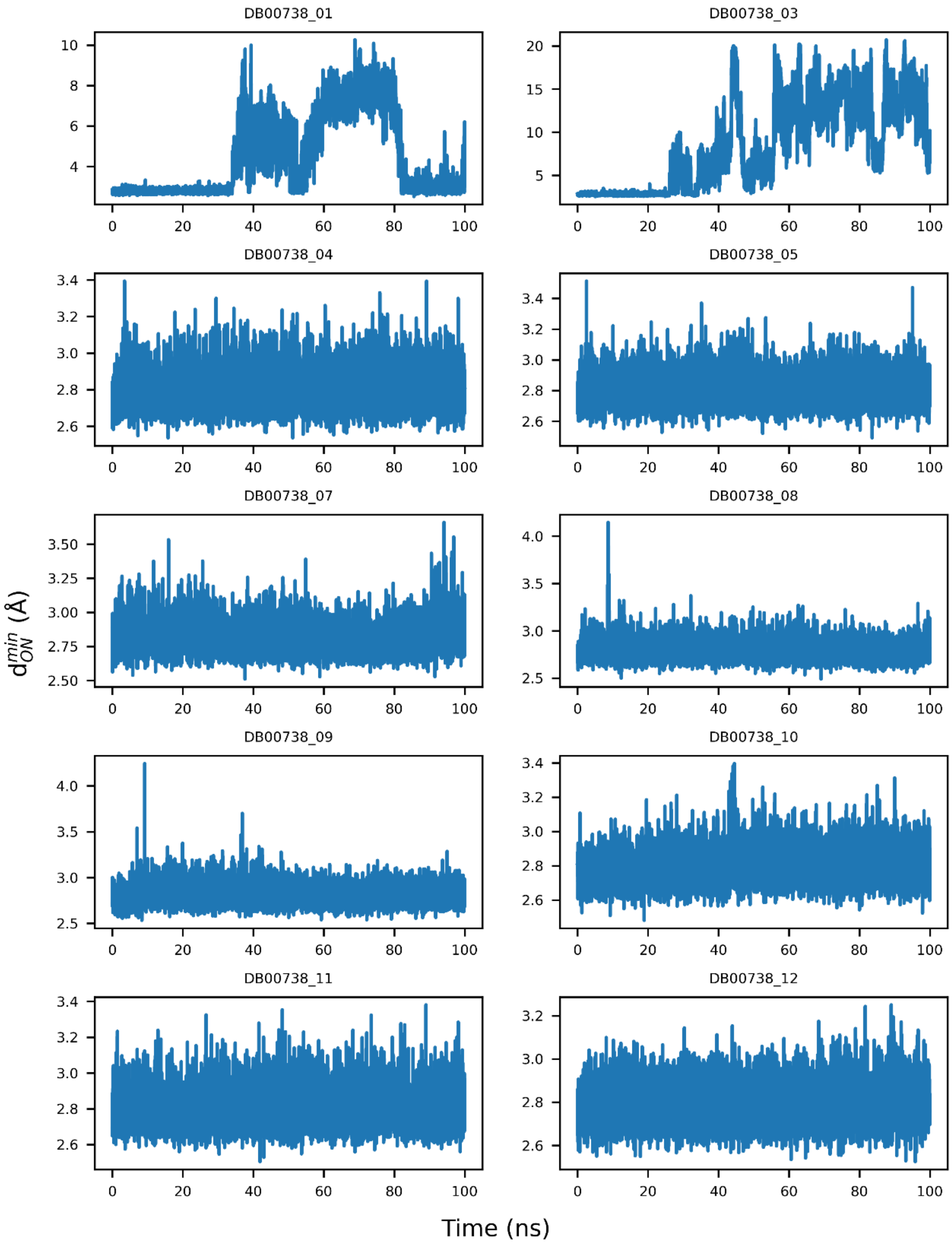

Figure S7. The d_{ON}^{min} distance calculated from 100 ns molecular dynamics simulation for each compound with different poses.

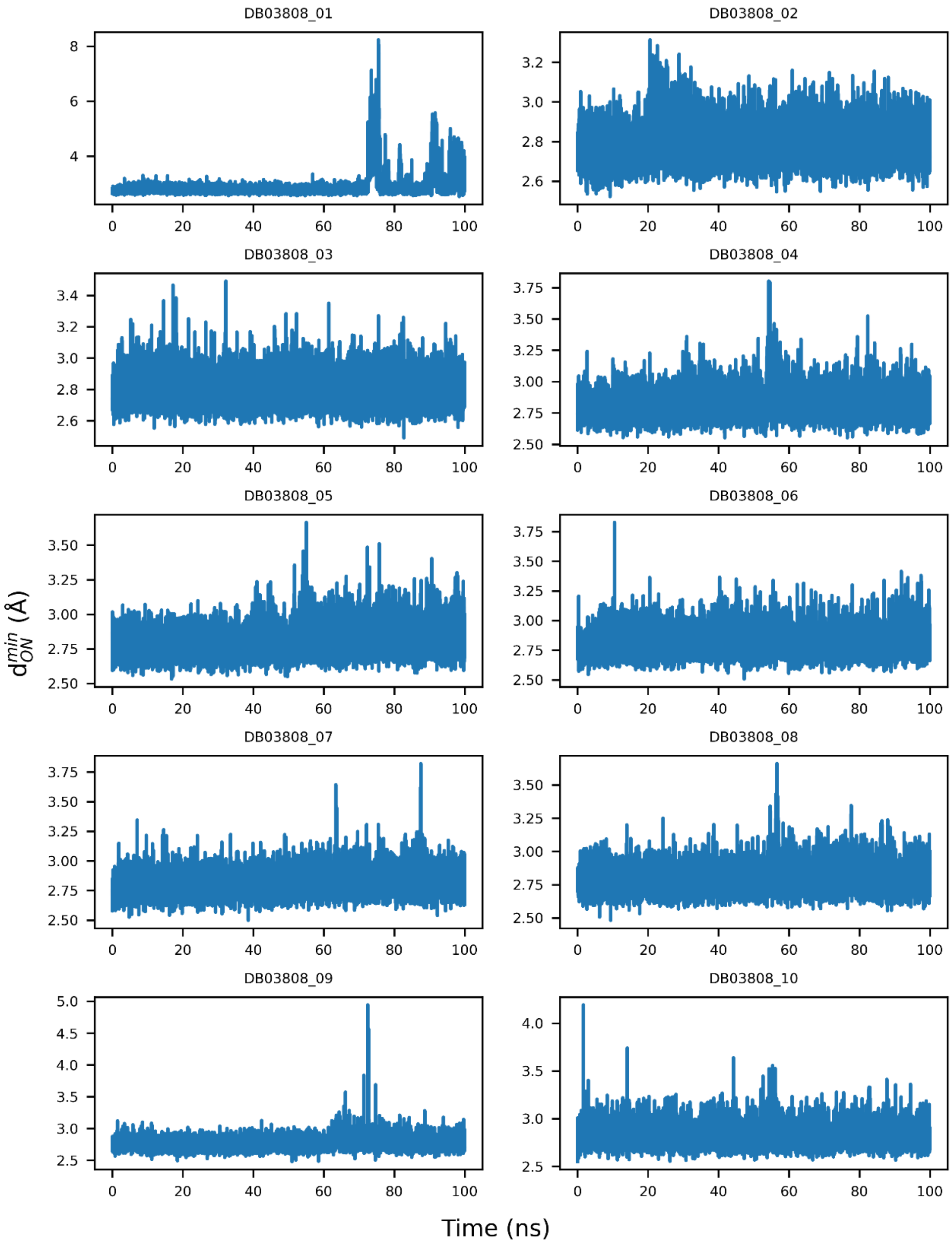

(Figure S7 continued)

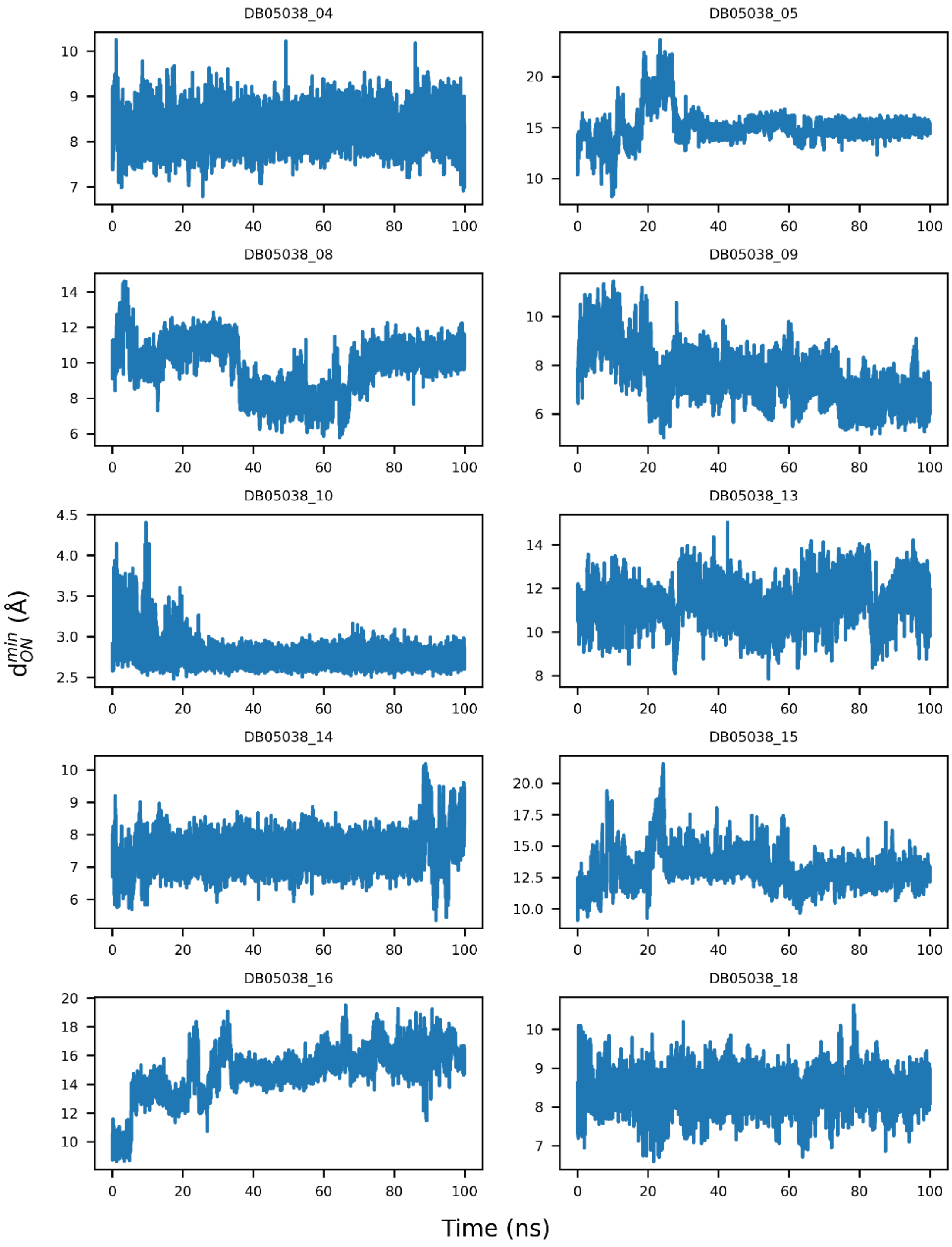

(Figure S7 continued)

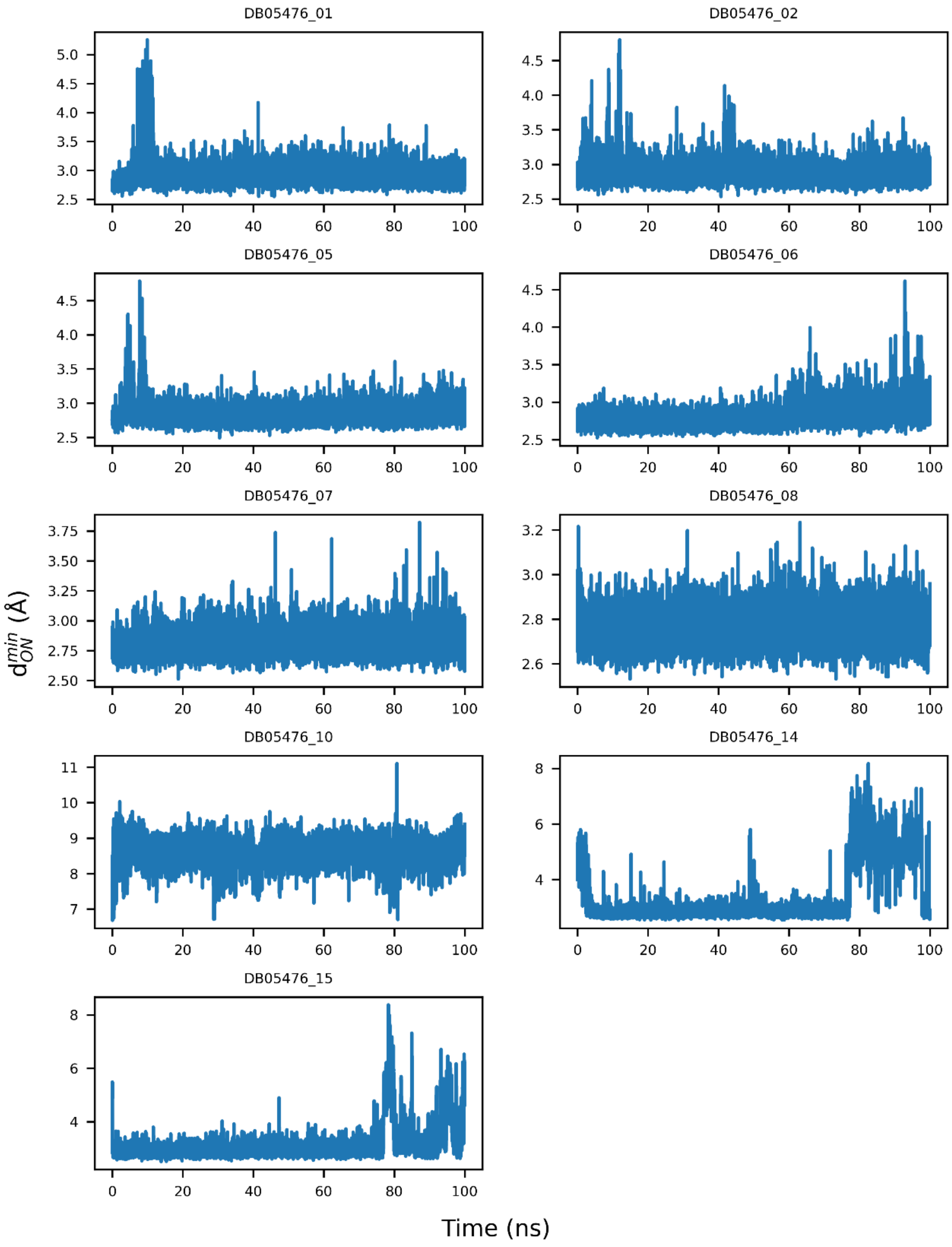

(Figure S7 continued)

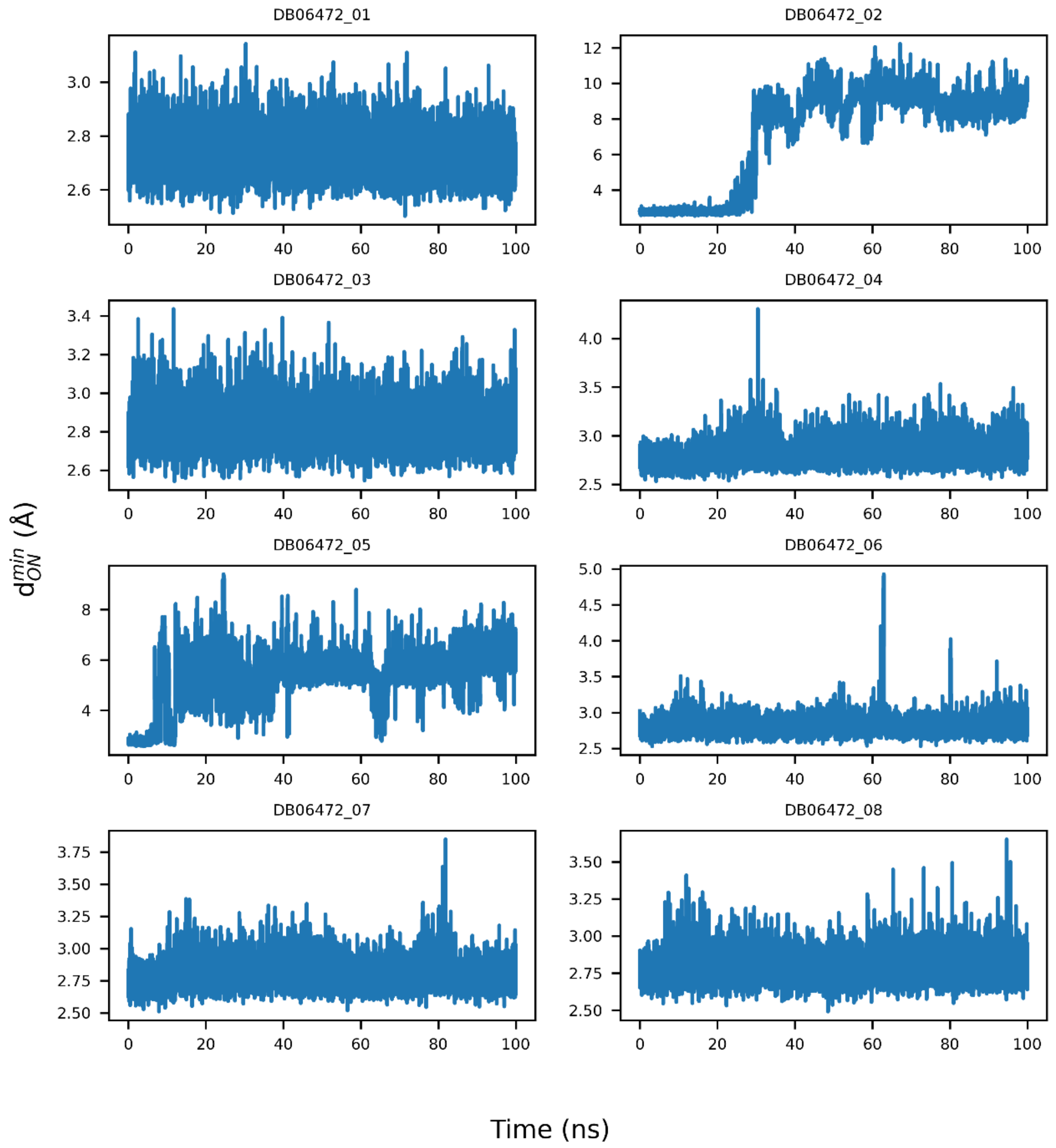

(Figure S7 continued)

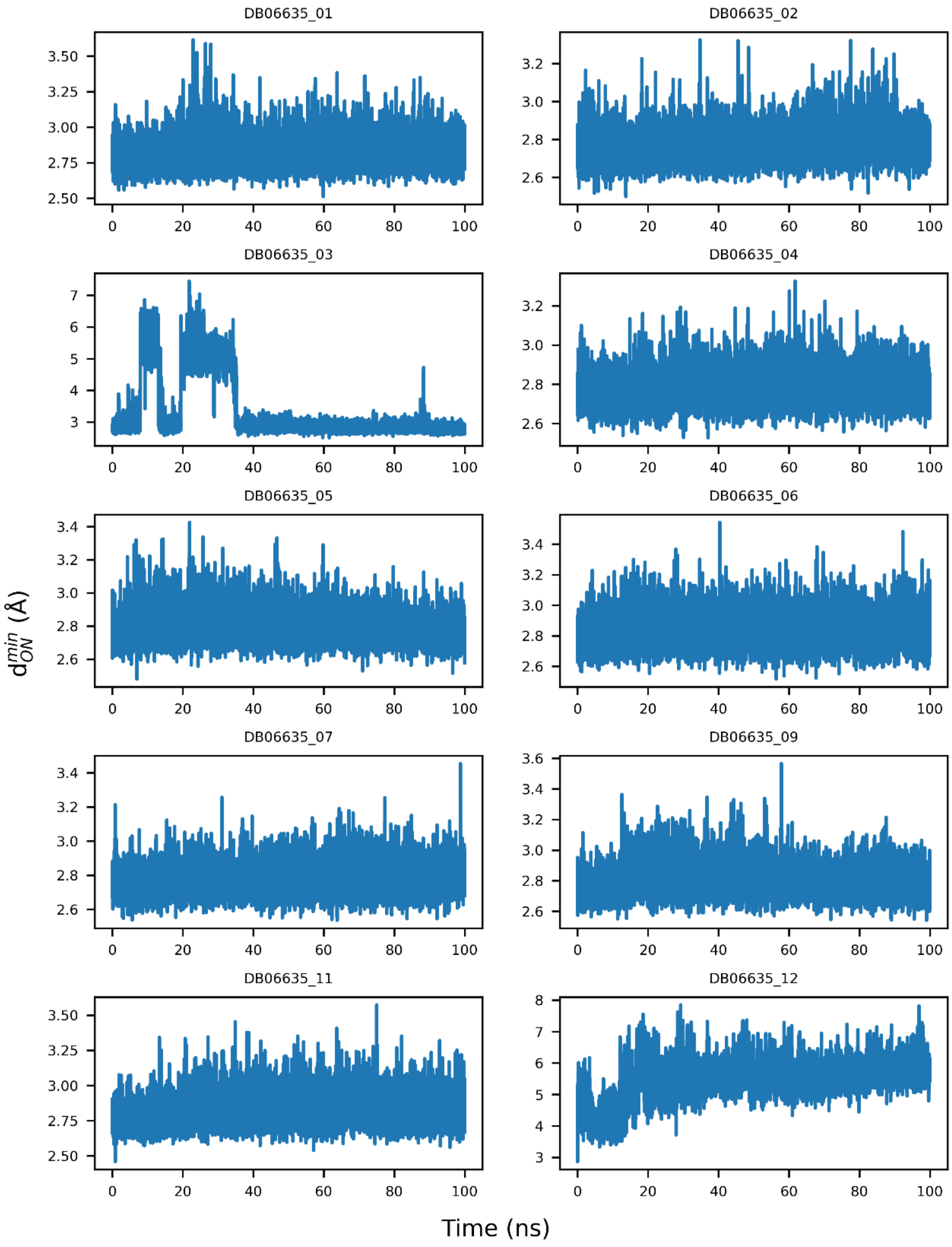

(Figure S7 continued)

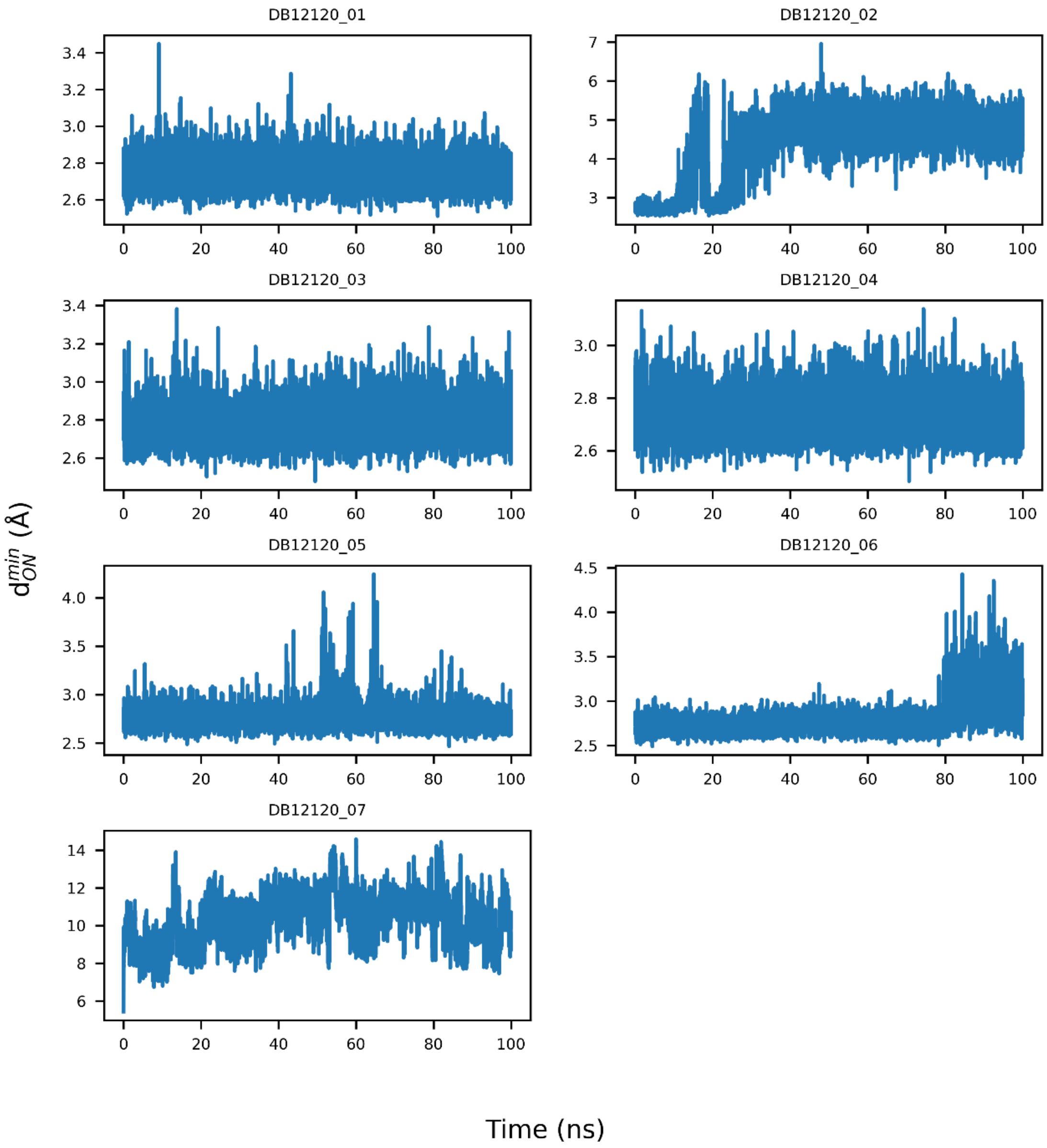

(Figure S7 continued)

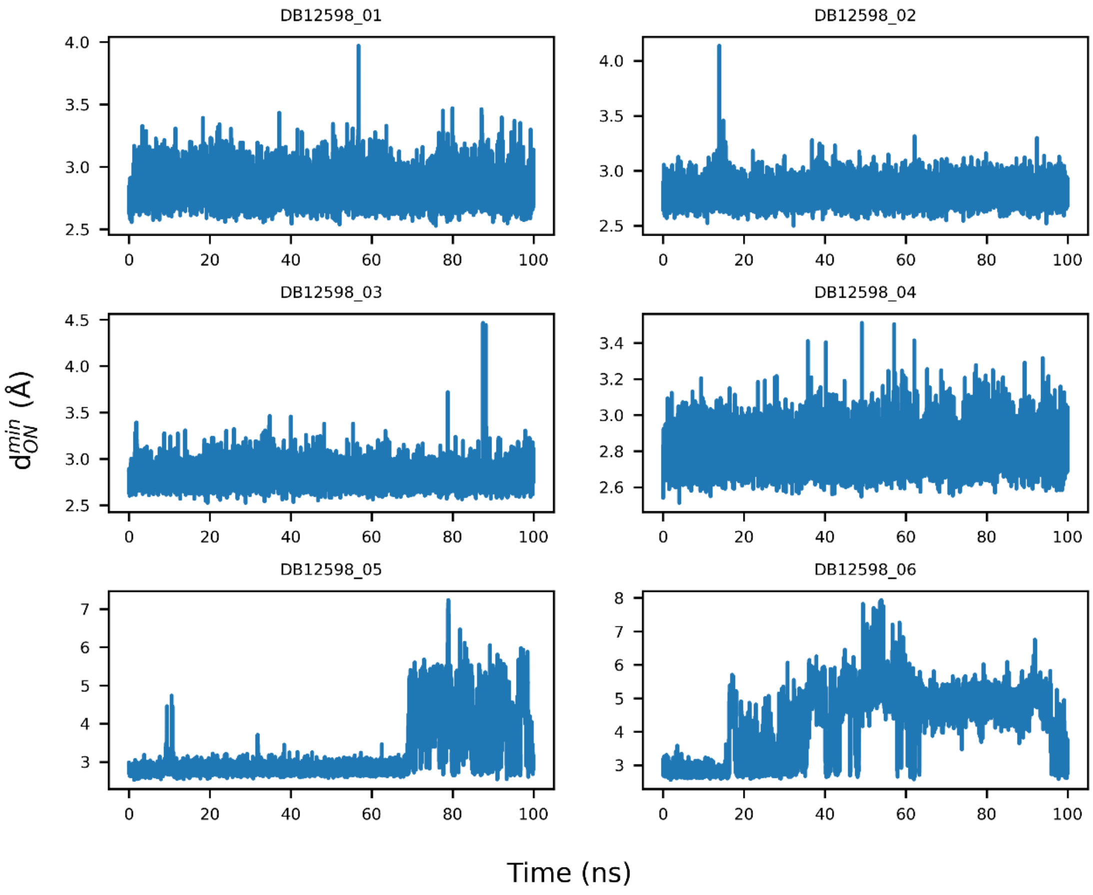

(Figure S7 continued)

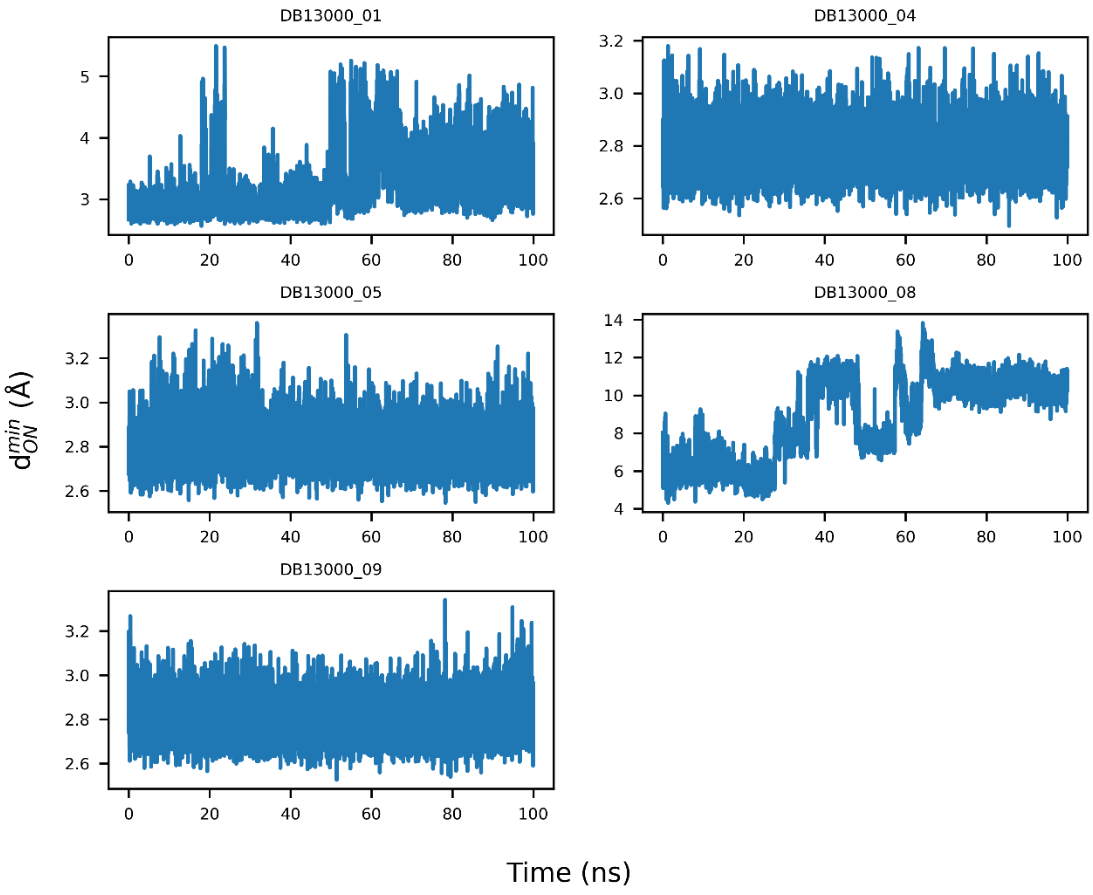

(Figure S7 continued)

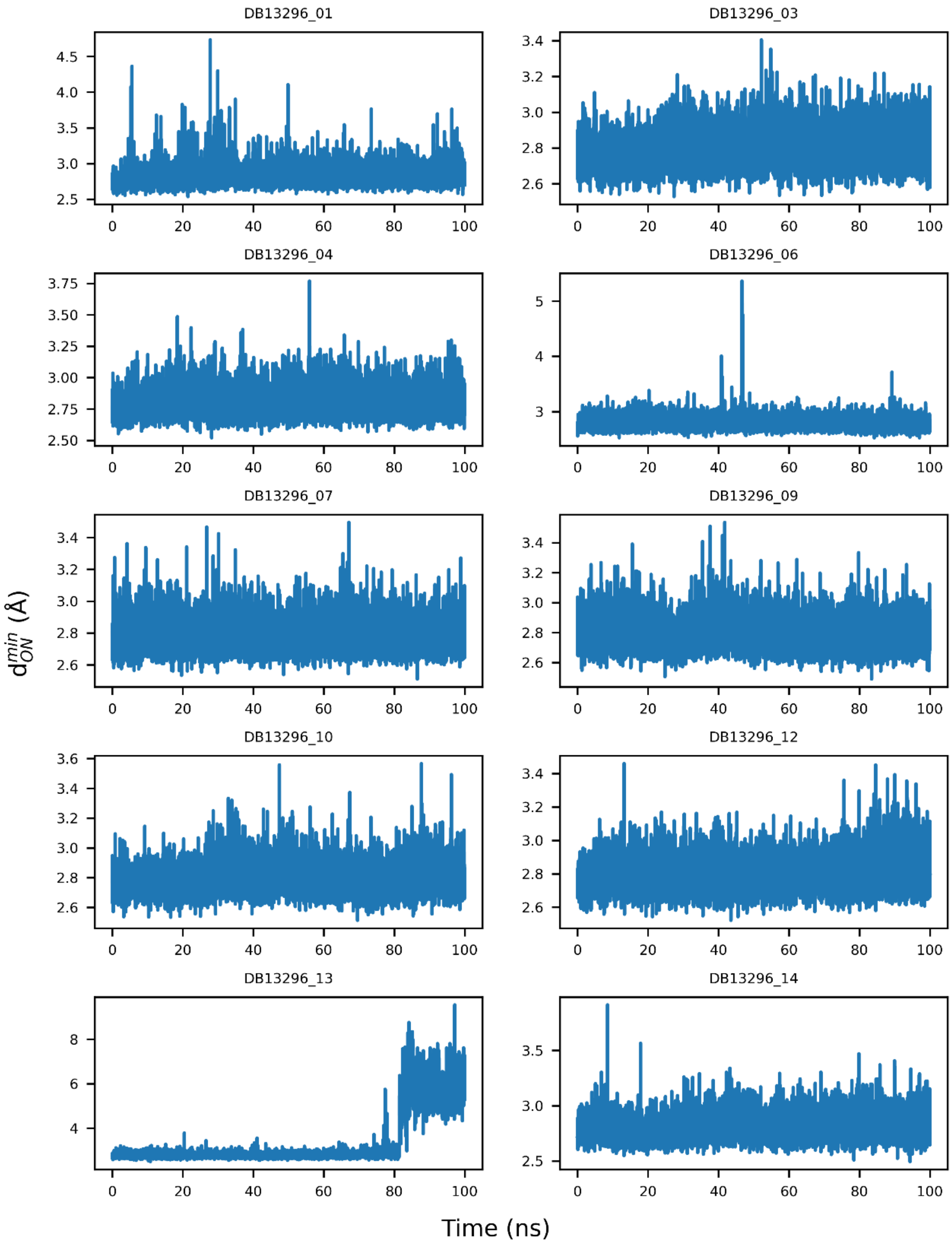

(Figure S7 continued)

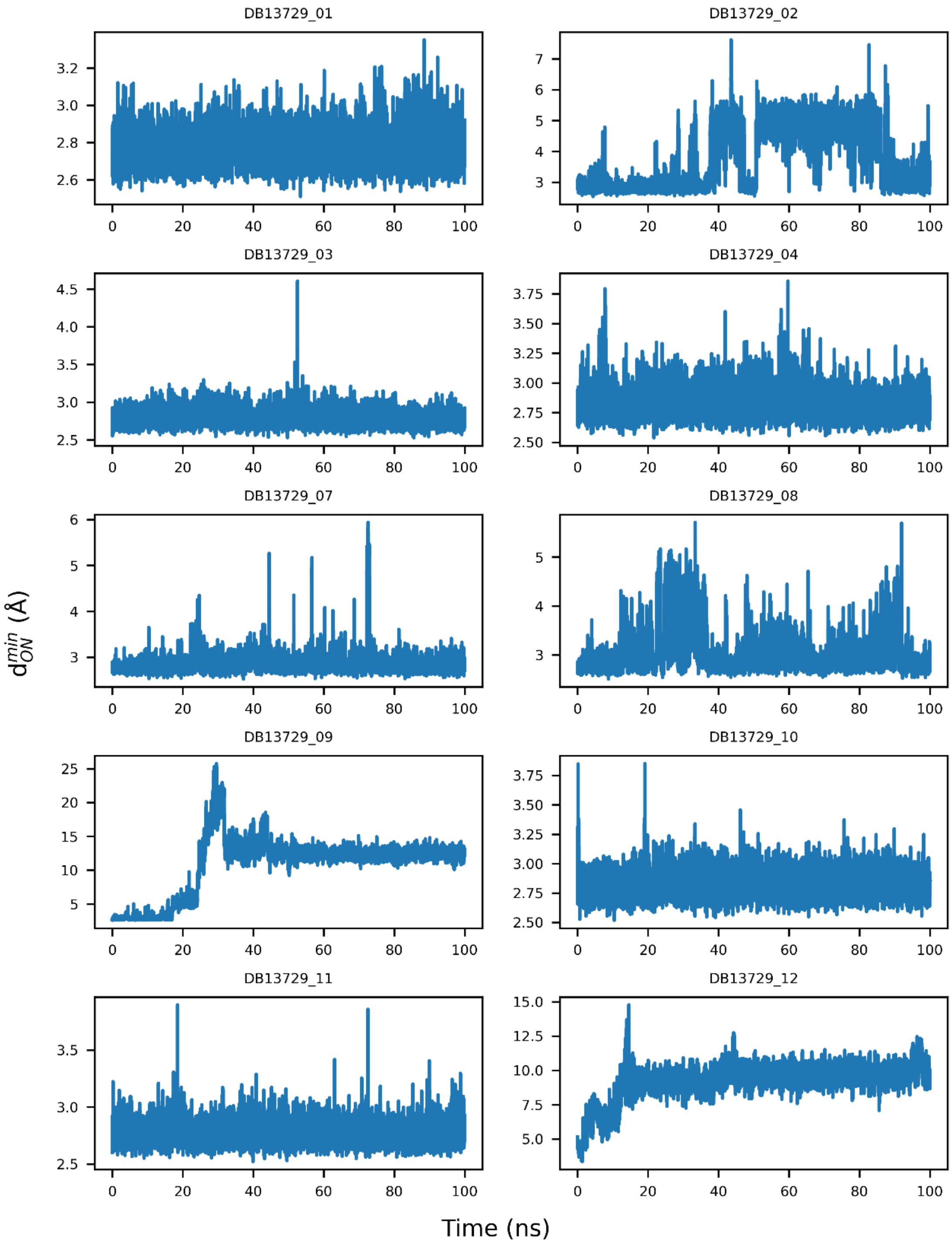

(Figure S7 continued)

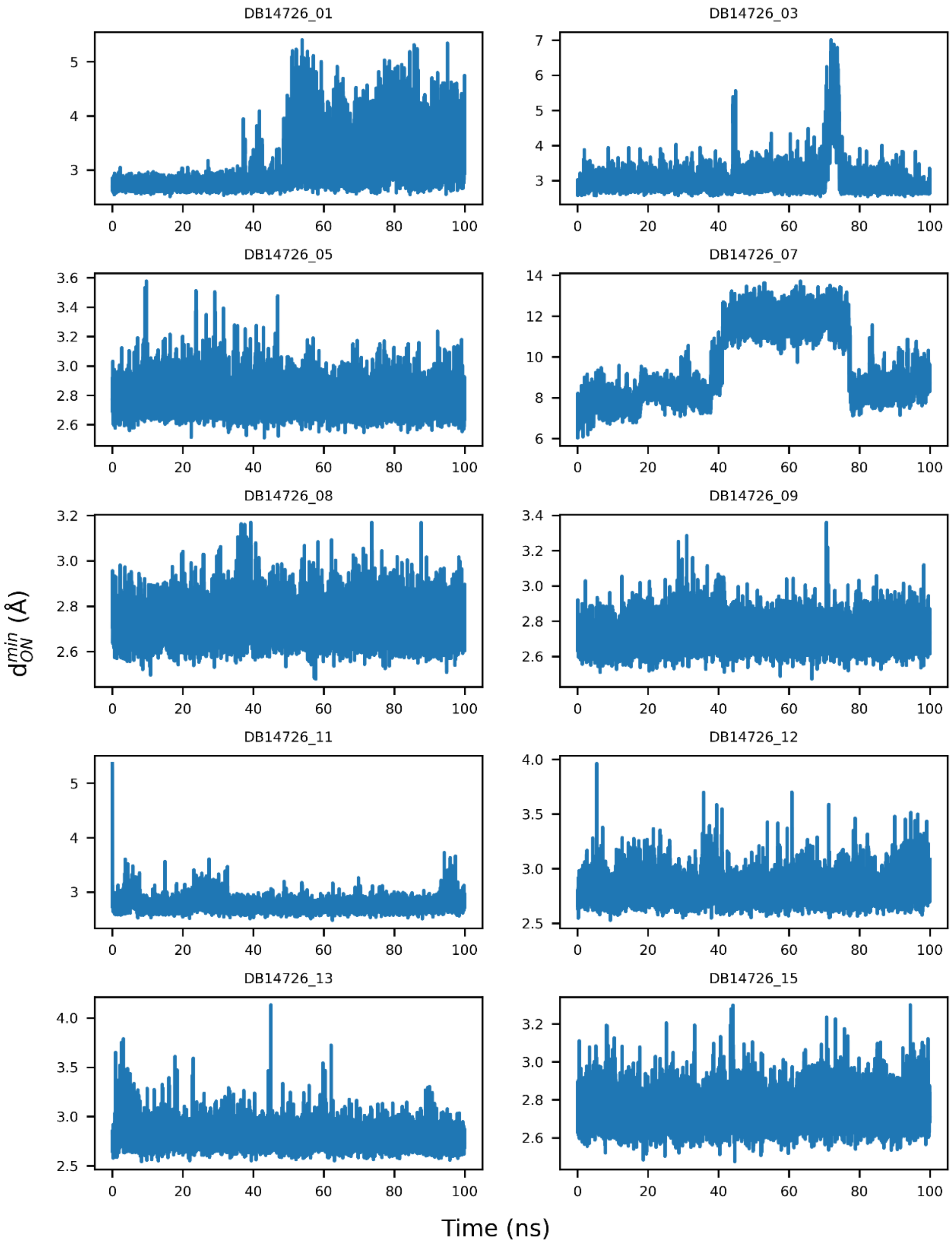

(Figure S7 continued)

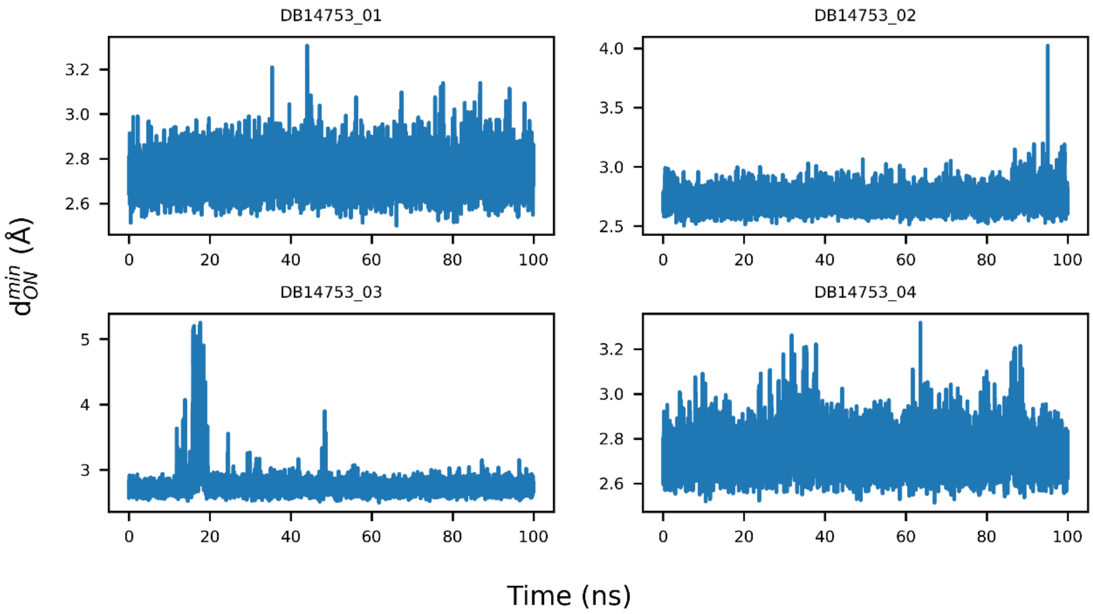

(Figure S7 continued)

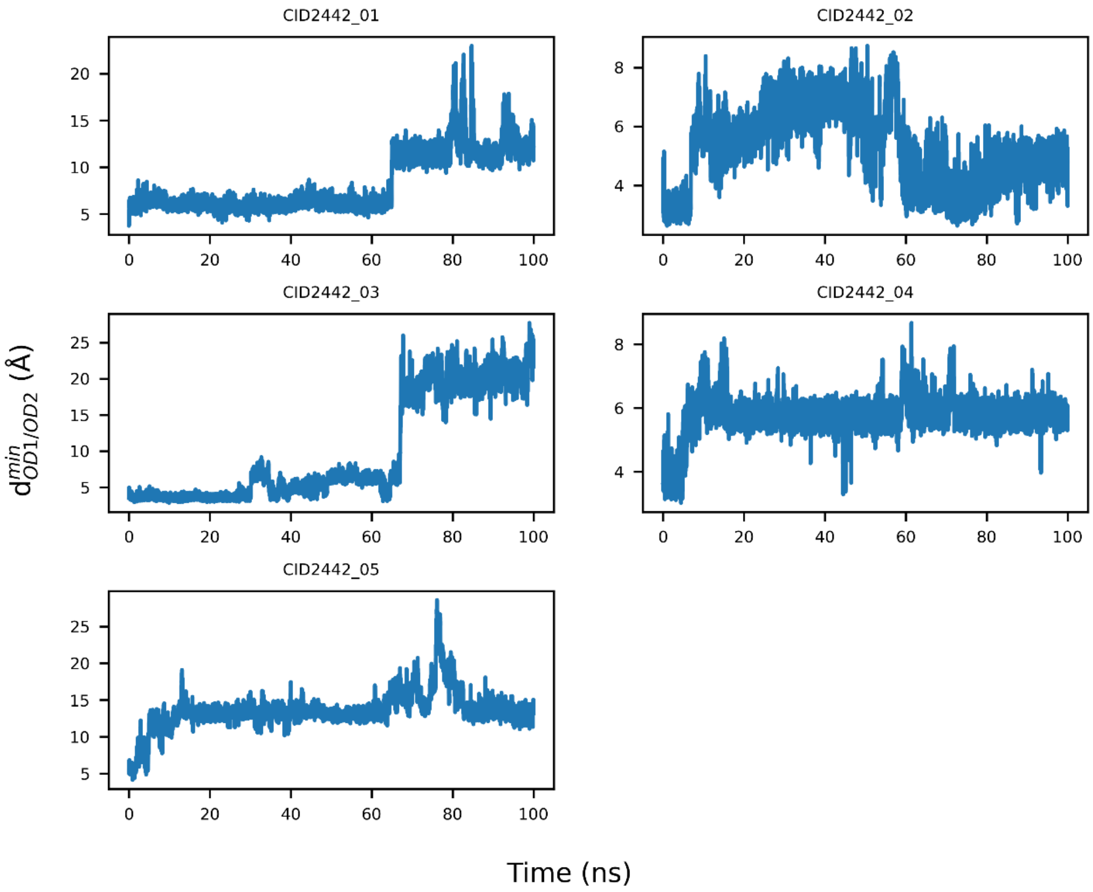

(Figure S7 continued)

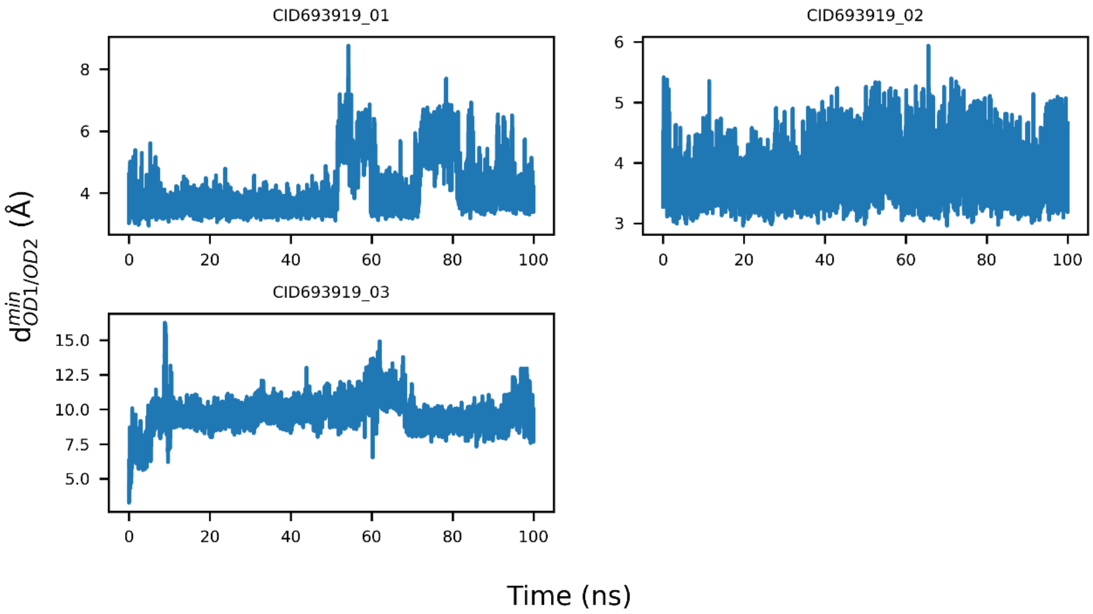

(Figure S7 continued)

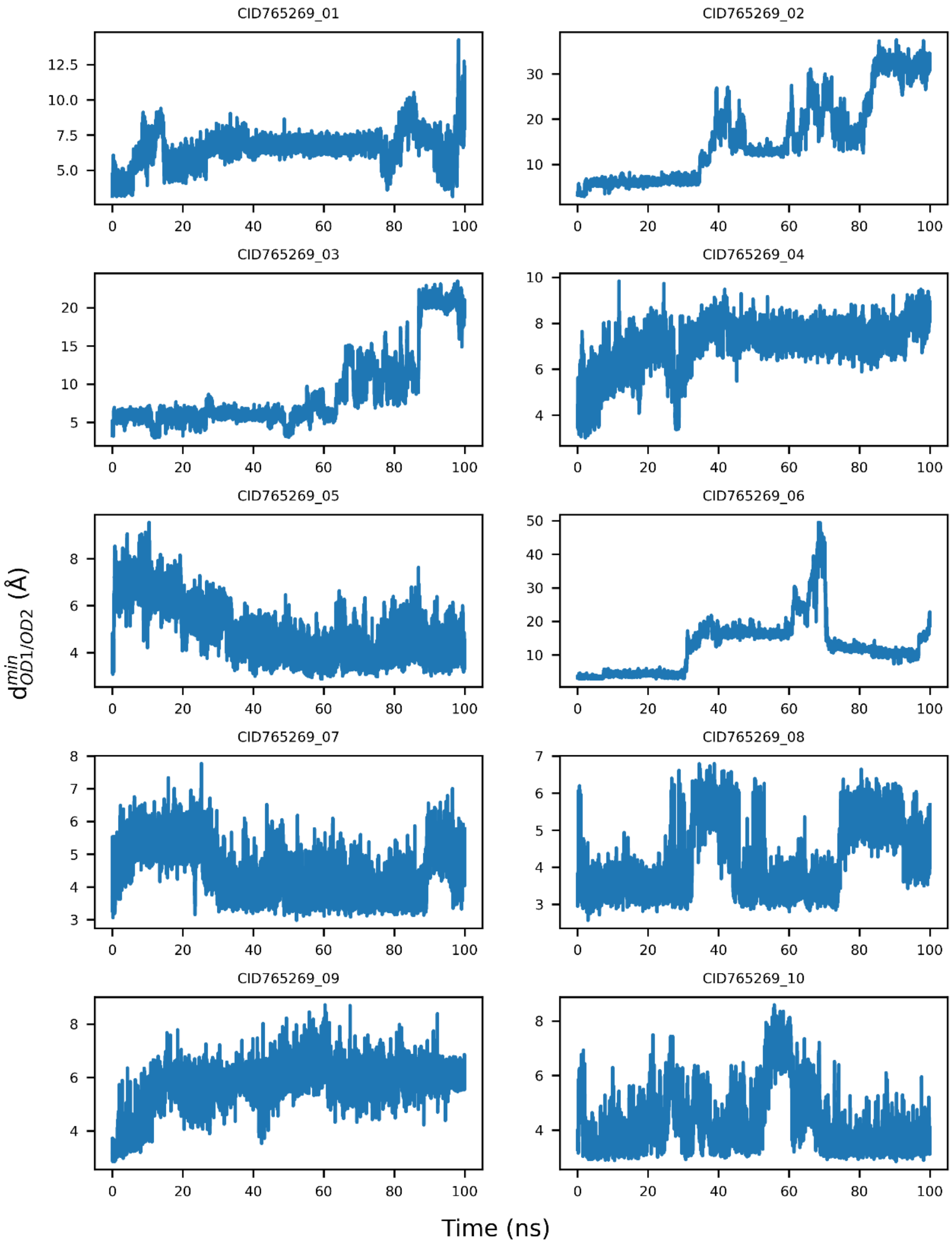

(Figure S7 continued)

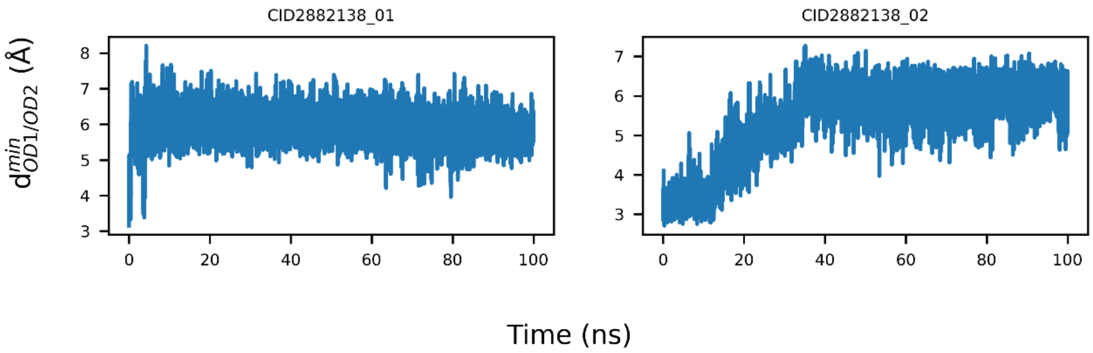

(Figure S7 continued)

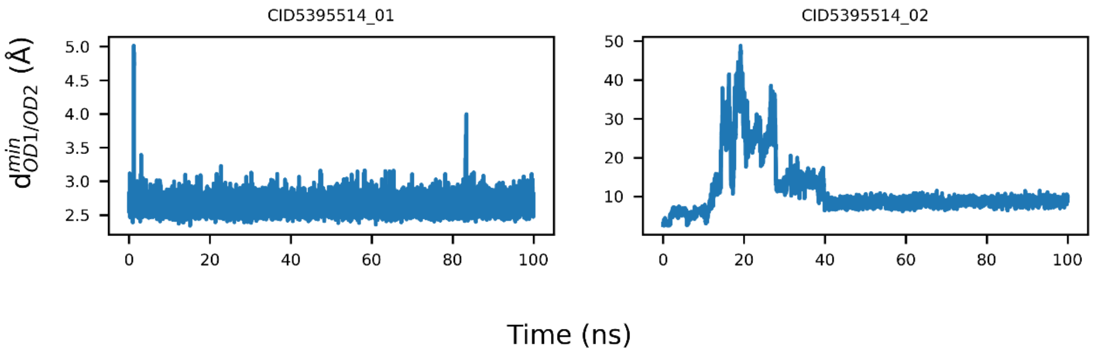

(Figure S7 continued)

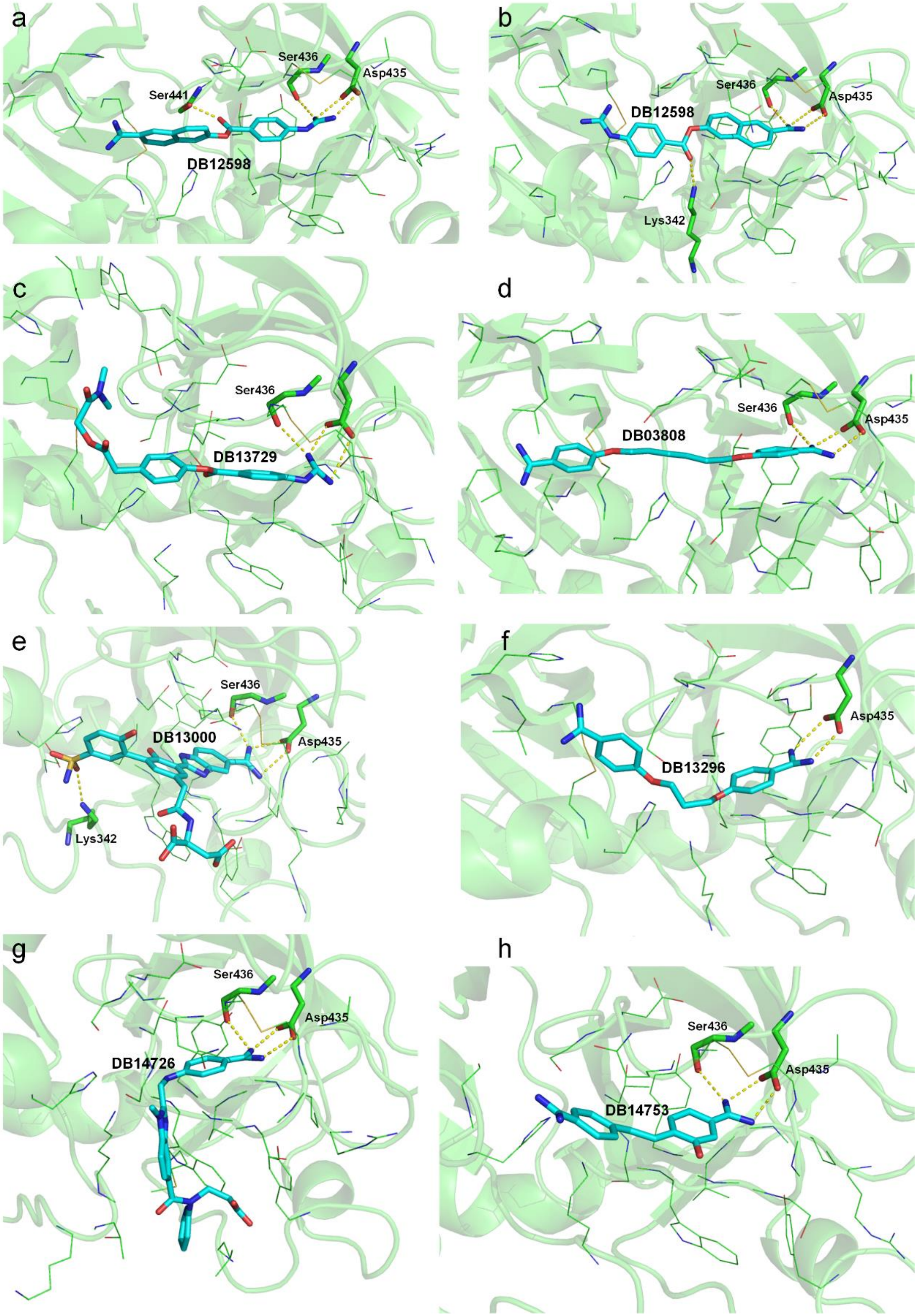

Figure S8. Salt-bridge and hydrogen-bonding interactions for other repurposed drugs at the end of the 100-ns MD simulations. Ligands and residues that are involved in hydrogen bonds or salt bridges (marked in yellow dashed lines) are shown in cyan and green sticks, respectively. Other residues are shown in lines.
